# Template‐Controlled Mechanochemical Dissociation of a Rotaxane

**DOI:** 10.1002/anie.202521995

**Published:** 2025-11-20

**Authors:** James Ormson, Tomás Nicolás‐García, Guillaume De Bo

**Affiliations:** ^1^ Department of Chemistry University of Manchester Oxford Road Manchester M13 9PL UK

**Keywords:** Dethreading, Force, Mechanophore, Rotaxane, Unstoppering

## Abstract

The mechanochemical dissociation of rotaxanes has been used in the design of materials with the ability to dissipate energy and of force‐controlled release systems. The former takes advantage of the dissociation by dethreading, a noncovalent process in which the macrocycle is forced over a stopper, while in the latter, the forceful contact between the macrocycle and stopper provokes the rupture of a covalent bond and release of the stoppering unit. As the dissociation pathway is dictated by the relative size of the macrocycle and the stopper, a rotaxane under tension can usually only undergo one of these two pathways. However, the ability to control the mechanochemical reactivity of a rotaxane in situ would allow the creation of multiresponsive materials. Here, we show how to selectively access unstoppering and dethreading pathways from the same rotaxane structure by respectively keeping or removing a single Pd atom to alter the size of the cavity of the macrocycle in a rotaxane built around a Pd^II^ template. We anticipate that these findings will have applications in the design of force‐responsive materials and devices in which the mechanical response can be tuned to suit specific uses.

## Graphical abstract

The presence or absence of the Pd template used in rotaxane formation selectively leads to two different mechanochemical dissociation pathways. The Pd maintains a smaller cavity size that favors dissociation by unstoppering. Removal of the Pd template atoms increases the size and flexibility of the macrocycle, which allows for disassembly by dethreading. The ability to selectively switch the mechanochemical behavior of rotaxanes could be used to control the mechanical properties of polymer materials.

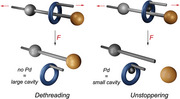

The ability of mechanical bonds to allow for the component parts to move with respect to each other has been harnessed for the development of responsive materials and molecular machines.^[^ ^1^ ^]^ When this behaviour is promoted by mechanical force this can lead to materials with the ability to dissipate mechanical energy.^[^ ^2^, ^3^, ^4^, ^5^, ^6^ ^]^ It was shown that the ability of a rotaxane to dethread at low force and behave as a sacrificial bond led to an increased toughness in rotaxane‐cross‐linked polymers.^[^ ^5^ ^]^ This ability has also been used to tune the photoluminescence properties of mechanochromic rotaxanes.^[^ ^7^, ^8^ ^]^ At high levels of force, we have shown that rotaxanes^[^ ^9^, ^10^ ^]^ and knots^[^ ^10^ ^]^ can both accelerate covalent bond scission within their architectures while catenanes^[^ ^11^ ^]^ are able to diffuse the applied stress. The accelerated scission of rotaxanes has led to the development of force‐controlled release devices^[^ ^13^, ^14^, ^15^, ^16^, ^17^, ^18^ ^]^ and highlights the potential of rotaxane‐containing materials to fail at high levels of force. Since the introduction of a Pd^II^ template rotaxane,^[^ ^19^ ^]^ this design has been widely used to develop polymers containing mechanical bonds.^[^ ^20^, ^21^, ^22^, ^23^, ^24^ ^]^ It was recently shown that metal coordination inside catenane‐based polymers can influence their mechanical performances compared to the corresponding demetalated material,^[^ ^22^, ^24^ ^]^ highlighting the ability to tune the mechanochemical response of a mechanical bond by the addition or removal of metal coordination. However, the change observed related only to the mobility of the catenated rings, not to their mechanochemical behaviour. As rotaxanes can break by unstoppering, whereby the elongation of the rotaxane promotes the scission of a covalent bond at the axle/stopper junction as the macrocycle pushes against the stopper,^[^ ^10^ ^]^ we hypothesised that the presence or removal of the Pd template used in the synthesis of the rotaxane, which affects the cavity size of the macrocycle, could influence its mechanical dissociation. Here we show how the presence or absence of a Pd atom inside the cavity of the macrocycle can switch the dissociation mechanism from unstoppering to dethreading respectively (Figure 1). The change in reactivity originates from the reduction in size of the macrocycle's cavity in the presence of a Pd atom. These results show how the mechanochemical reactivity of a rotaxane can be easily and selectively controlled with the addition of a single atom. We anticipate that such an approach will aid the future design of force‐responsive devices and materials based on rotaxanes and allow for the response to be tuned depending on the desired use, potentially in response to external stimuli.

**Figure 1:**
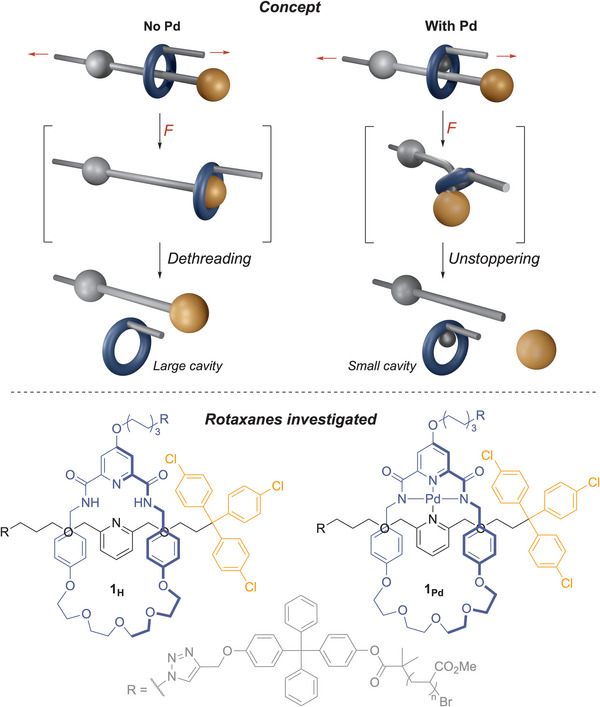
The presence or absence of the Pd template used to assemble rotaxanes **1_Pd_** and **1_H_** selectively switch their mechanochemical dissociation pathway from a covalent unstoppering process (**1_Pd_**) to a noncovalent dethreading process (**1_H_**), respectively.

Our design is based on a rotaxane assembled around a Pd^II^ template using an established strategy where a pseudo‐rotaxane is formed by the assembly of a square‐planar Pd^II^ complex from a macrocyclic tridentate pyridyl‐2,6‐dicarboxyamide ligand, and an axle containing a monodentate pyridine ligand (Figure 2).^[^ ^19^ ^]^ The axle is terminated on one side with a bulky tris(4‐chlorophenyl)methyl stopper, the active stopper used in this study, and on the other side with an azide, which is subsequently used to install the second stopper on the axle, as well as polymerisation initiators on both the axle and the macrocycle (Figure 2). This ensures that the rotaxane is placed at the centre of the polymer chain where mechanical activation is most likely to occur.^[^ ^25^ ^]^ The demetalated rotaxane is obtained by treatment with KCN prior to the polymerisation step (see Supporting Information for details). Chain‐centred macromolecular rotaxanes **1_H_** and **1_Pd_** (Figure 1) were obtained by single electron transfer living radical polymerisation (SET‐LRP)^[^ ^26^ ^]^ of methyl acrylate (Table 1). This design allowed us to regulate the size of the cavity of the macrocycle depending on the presence or not of the Pd template atom. In effect, the presence of the Pd reduces the pyridyl‐2,6‐dicarboxyamide/crown ether macrocycle from a 30‐ to a 26‐membered ring (Figure 2). In the absence of force, the tris(4‐chlorophenyl)methyl stopper is much larger than the cavity of either macrocycle, making dethreading impossible in force‐free conditions. Upon mechanical activation, the smaller Pd macrocycle should be pulled towards the terminal stopper upon dissociation of the pyridine‐Pd bond, which occurs at low force (*F_EFEI_* ∼ 1.5 nN, see Supporting Information). Further pulling should put the macrocycle in contact with the stopper until bond scission occurs. Indeed, if the cavity is too small to allow the macrocycle to pass over the stopper (dethreading), the elongation of the rotaxane will induce a high level of tensile and torsional deformation in the axle that ultimately leads to the rupture of a covalent bond at the axle/stopper junction (Figure 1).^[^ ^10^ ^]^ Removing the Pd template opens up the cavity and increases the flexibility of the macrocycle to allow for its mechanical dethreading (Figure 1).

**Figure 2:**
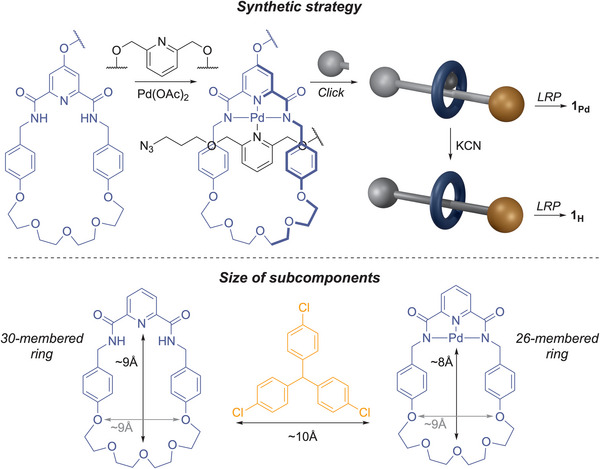
Synthetic strategy for the template synthesis of **1_Pd_** and **1_H_**. The relative size of subcomponents shows the effect of the Pd template on the size of the macrocycle's cavity. The selected nucleus–nucleus distances shown were taken from rotaxane models in the early stage of the MD simulations (Figure 4).

**Table 1:** Activation parameters for polymers **1_Pd_** and **1_H_**.

			Dissociation pathway^a)^		
Rotaxane	Pre‐sonic. *M*_n_ (kDa)/Ð	Post‐sonic. *M*_n_ (kDa)/Ð^a)^	Unstop. (%)	Dethread. (%)	N/R (%)
**1**_Pd_	146/1.20	66.0/1.27	77	0	23
**1**_H_	129/1.17	58.9/1.44	0	78	22

^a)^ Average value from three parallel experiments.

The mechanical activation of rotaxanes **1_Pd_** and **1_H_** was performed in MeCN at 5–10 °C, using high‐intensity ultrasound (20 kHz, 10.4 W cm^−2^, 1s ON/1s OFF, 60 min). The progress of the reaction was monitored by SEC and the dissociation pathway was determined by ^1^H NMR spectroscopy of the polymer after sonication and of the axle fragments which were isolated by washing the polymer residue with methanol (Figure 3). For **1_Pd_**, the mechanical bond dissociates in a conversion of 77% (average of three runs), which was determined by comparing the integration of the macrocycle aromatic protons H_a_, which shift downfield in its non‐interlocked form due to a loss of the π–π interactions present in the rotaxane. Some of the macrocycles (20%) also show the loss of the Pd metal from the tridentate ligand, as indicated by the presence of a second set of aromatic peaks (H_a’_), which was found to occur as a side effect of sonication rather than from the mechanical activation itself, the absence of signals corresponding to reference **6** shows that there was no dethreading and all demetallation occurs after unstoppering (see section 5, Supporting Information). In other words, the Pd complexation of the macrocycle survives the contact with the stopper, thereby ensuring the selectivity of the unstoppering dissociation as a premature loss of the Pd atom would lead to dethreading (see below). The unstoppering pathway was confirmed with the presence of dichlorobenzophenone **4** (H_d_), directly after sonication, and trichlorotrityl alcohol **3** (H_e_) in the methanol wash phase, two products resulting from the generation of the corresponding trityl radical (see Scheme S3) upon scission of the terminal C─C bond (red bond in Figure 3a).^[^ ^27^, ^28^ ^]^ Intriguingly, the absence of signals belonging to the ethylene spacer between the axle pyridine and the stopper (H_b‐c_), the axle pyridine (H_h_), and the methylene protons either side of the axle pyridine (H_i‐j_) after sonication, suggests the elimination of the pyridine fragment after the first mechanochemical bond scission—a phenomenon we have observed previously.^[^ ^10^ ^]^ Nevertheless, protons H_f_ and H_g_ can still be observed after sonication showing that the side of the axle closest to the polymer remains intact after activation. The picture is much simpler for demetalated rotaxane **1_H_** (Figure 3b), which disassembles by dethreading in a conversion of 78% (average of three runs). The selectivity of the process is confirmed by the restoration of the signals pertaining to the intact axle and macrocycle in the post‐sonication ^1^H NMR spectrum (Figure 3bii).

**Figure 3:**
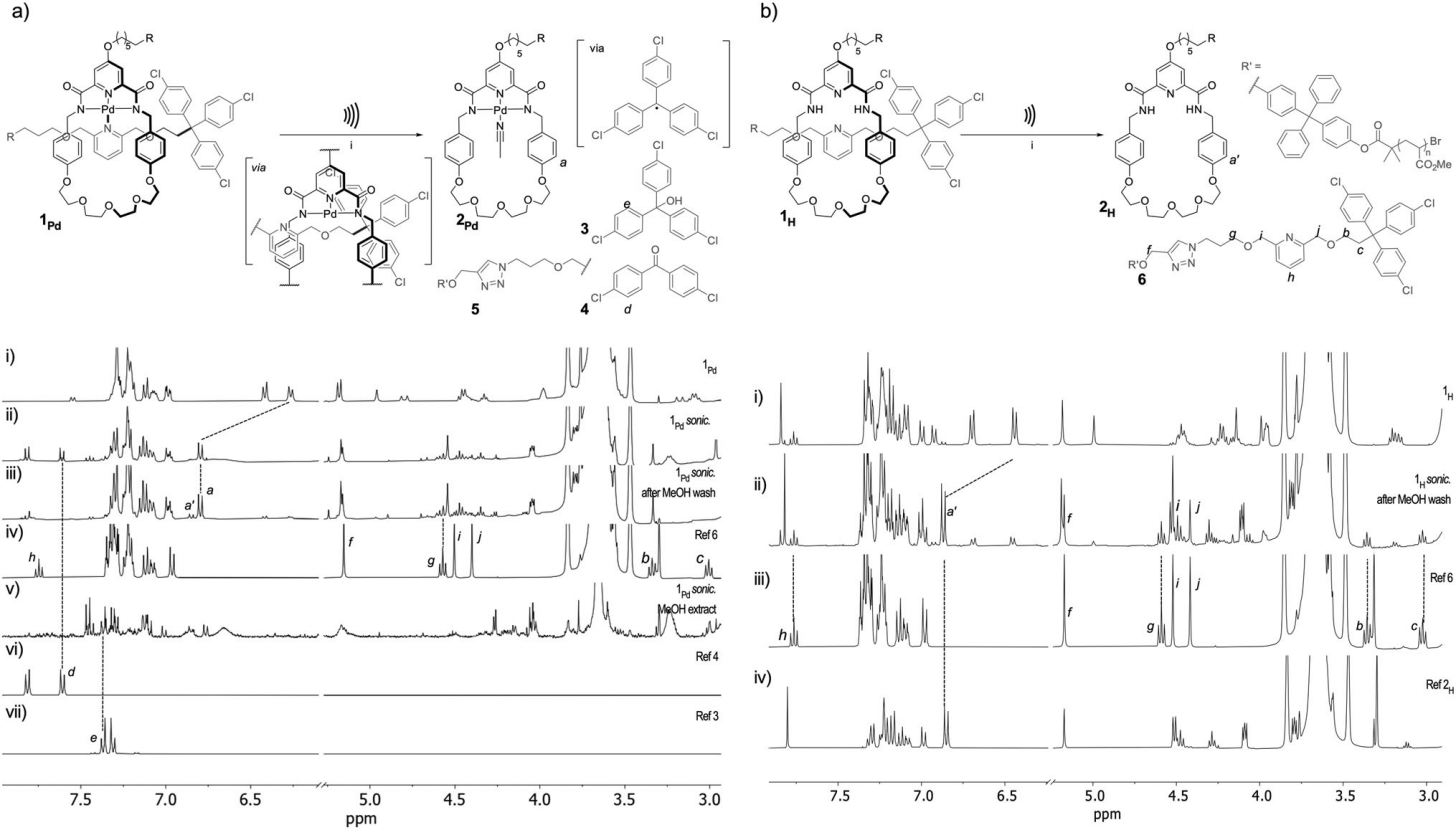
Mechanochemical activation of rotaxanes. a) Activation of **1_Pd_** leads to unstoppering. Partial ^1^H NMR (400 MHz, acetone‐*d*_6_, 298 K) spectra before (i) and after (ii) sonication. With MeOH‐washed polymer (iii) compared to axle **6** (iv) and methanol phase (v) compared to references of benzophenone **4** (vi) and trityl alcohol **3** (vii). b) Activation of **1_H_** leads to dethreading. Partial ^1^H NMR (400 MHz, acetone‐*d*_6_, 298 K) of rotaxane polymer before (i) and after sonication and MeOH wash (ii) compared to axle **6** (iii) and macrocycle **2_H_** (iv) references. Scissile bonds are indicated in red.

The selectivity of these two dissociation pathways associated with the absence or presence of the Pd template is remarkable, as other mechanochemical reactions could potentially compete with the unstoppering and dethreading observed. For example, the mechanical demetallation of **1_Pd_** into **1_H_** could be followed by a dethreading disassembly. Similarly, one could have expected the dethreading of **1_H_** to compete with an unstoppering pathway as the trityl stopper presents a very large steric obstacle, with its three projecting aromatics, compared to the size of the macrocycle (Figure 2). In fact, static computational methods (CoGEF^[^ ^29^, ^30^ ^]^ and EFEI^[^ ^30^ ^]^) failed to predict the dethreading behaviour of **1_H_** (see section 6, Supporting Information), and rather predicted the unstoppering of both **1_H_** and **1_Pd_** models at *F_max_* ∼ 4.2 nN at the scissile bond shown in Figure 3a. The initial scission of the Pd pyridine bond was found at ∼ 1.5 nN by EFEI. This suggests that the macrocycle of **1_H_** is probably unable to pass over the stopper at once and that thermal and/or dynamic effects are involved in this process.

To investigate these effects, we performed molecular dynamics simulations (EFEI, GFN2‐xTB, vac)^[^ ^17^, ^31^, ^32^ ^]^ at two levels of force (3.0 and 3.5 nN) on rotaxane models **1_Pd_** ′ and **1_H_** ′ (Figure 4 and Videos S1–S4). In **1_Pd_** ′, the unstoppering behaviour was the only observed dissociation pathway, accounting for 60% and 100% of the 20 trajectories at 3.0 and 3.5 nN, respectively (Table 2). Covalent bond scission either occurred at the axle/stopper junction (bond *a*) or at the adjacent bond *b* (*a*/*b* = 1/3 at 3.0 nN, see section 6, Supporting Information for details). A representative trajectory is shown in Figure 4a and Videos S1, S2. The unstoppering behaviour can be visualised by tracking the position of the marked O and Cl atoms (in blue and yellow in Figure 4), on the macrocycle and stopper respectively, in relation to a fixed reference point (red sphere, Figure 4) in the MD space. As force is exerted on the rotaxane (after an equilibration plateau ending at the 1000 fs mark), the O and Cl positions converged as the macrocycle approached the stopper, a motion made possible by the rupture of the Pd‐N_pyr_ interaction, which resulted in a small increase and subsequent plateau at ∼150 kJ mol^−1^ in the energy profile. The forceful contact between the macrocycle and the stopper eventually led to the unstoppering process, characterised by a sharp peak in the energy profile (771 kJ mol^−1^) and the synchronised motion of the marked atoms from the reference point (Figure 4a) as both the macrocycle and stopper moved away from the axle. In the case of demetalated rotaxane **1_H_** ′, dethreading was observed in 45% of the trajectories and unstoppering in 25% at 3.0 nN (Table 2), with the remaining trajectories exhibiting no reactivity. Tracking the same marked atoms (Figure 4b), the dethreading can be visualised by the atom positions crossing over as the macrocycle passes over the stopper, which results in a shallower energy profile peaking at 639 kJ mol^−1^. The lower energy of the dethreading pathway accounts for why this is the only observed pathway experimentally. In fact, unstoppering is more prevalent at higher forces (Table 2). As hypothesised above, not only does the presence of the template Pd atom reduce the size of the macrocycle (Figure 2), but it also hampers its deformability. Indeed, unlike **1_Pd_** ′, the macrocycle in **1_H_** ′ was able to stretch its cavity to a length of ∼13 Å, which is substantially longer than the Cl–Cl distance in the stopper (Figure 4c). Although the cross section of the stopper is still larger than the macrocycle's (as the width of the macrocycle remains narrow throughout the elongation process, see Figure 4c), this deformation nevertheless allowed one arm of the stopper to pass through the cavity first (Figure 4bi,ii and Videos S3, S4). After this, the macrocycle easily slipped over the two remaining aromatic groups (Figure 4bii,iii and Videos S3, S4). This stepwise dethreading can be seen in the energy profile (Figure 4b), which peaked as the first arm of the stopper passes through the cavity of the macrocycle, and then plateaued and decreased gain as the macrocycle slipped over the remaining aromatic units. The fact that the dethreading process relies on the initial rotation of the stopper to allow for passage of the central aromatic group through the macrocycle cavity (see Videos S3, S4) explains the failure of static calculation methods to predict dethreading and the need to take molecular motions into account.

**Figure 4:**
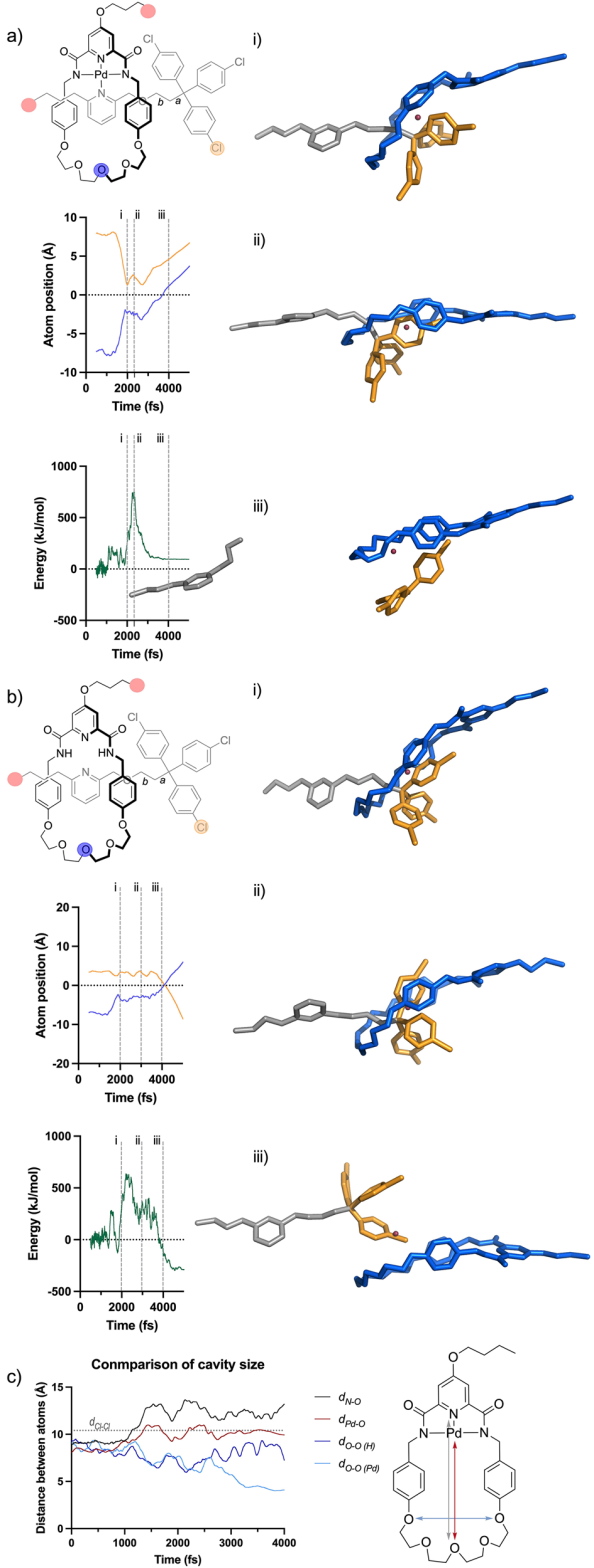
Computational investigation of rotaxane dissociation pathways. Representative MD (EFEI@3.00 nN, GFN2‐xTB) trajectories of **1_Pd_'** (a) and **1_H_'** (b). Comparison of macrocycle cavity dimensions in the trajectories depicted, the dashed line indicates the width of the stopper c). Pulling points are indicated with pink discs. Red spheres in (a) and b) indicate the reference point for atom position tracking.

**Table 2:** Summary of MD trajectories.

		Dissociation pathway		
Rotaxane	*F* (nN)	Unstop. (%)	Dethread. (%)	N.R (%)
1_Pd_^'^	3.0	60	0	40
	3.5	100	0	0
1_H_^'^	3.0	25	45	30
	3.5	80	20	0

In conclusion, we have demonstrated that two different dissociation pathways can be accessed selectively from the same core rotaxane architecture depending on the presence or absence of a single Pd atom used as a template in the formation of the rotaxane. The presence of the Pd template maintains a smaller cavity size in the macrocycle, which is conducive to rotaxane dissociation by unstoppering, whereby the terminal stopper is covalently cleaved from the axle by the pushing action of the macrocycle. The removal of the Pd template atom increases the size and flexibility of the macrocycle, which allows the rotaxane to disassemble by dethreading as the macrocycle can slip over the stopper without covalent bond scission. This small change allows for a lower force dethreading behaviour of the rotaxane, which is impossible when the cavity is occupied. The stepwise dethreading of the macrocycle over a stopper larger than its own cavity was explained using molecular dynamics calculations, highlighting the importance of considering molecular motions to accurately model the mechanochemical behaviour of rotaxanes. The ability to selectively switch the unstoppering behaviour on and off by occupying the macrocycle cavity could have uses in the development of force‐controlled release systems, adding an additional method to control the unstoppering process when desired. This behaviour may also find use in rotaxane‐based materials in which the ability to allow for dethreading, and therefore altering the mechanical toughness or photophysical properties, can be controlled.

## Supporting Information

The authors have cited additional references within the Supporting Information.^[^ ^33^, ^34^, ^35^, ^36^, ^37^ ^]^

## Author Contributions

J.O. performed the experimental work. J.O. and T.N.G. performed the MD calculations. G.D.B. directed the research. J.O. and G.D.B. wrote the manuscript.

## Conflict of Interests

The authors declare no conflict of interest.

## Supporting information



Supporting Information

Supporting Information

Supporting Information

Supporting Information

Supporting Information

## Data Availability

The data that support the findings of this study are available in the Supporting Information of this article.
