# Supplementary material for: Template‐Controlled Mechanochemical Dissociation of a Rotaxane
Source: Angew Chem Int Ed Engl. 2025 Nov 20;65(2):e21995. doi: 10.1002/anie.202521995 (PMC12790351; doi:10.1002/anie.202521995)
Supplement: Supplementary file 1 — Supporting Information [file ANIE-65-e21995-s005.pdf]

Supporting Information

Template-Controlled Mechanochemical Dissociation of a Rotaxane

*James Ormson, Tomás Nicolás-García, and Guillaume De Bo**

Department of Chemistry, University of Manchester, Oxford Road, Manchester, M13 9PL, UK

*E-mail: guillaume.debo@manchester.ac.uk

1 Table of contents

1 Table of contents 2
2 General Experimental Details 5
3 Synthesis of mechanophore, control and reference compounds 6
3.1 Synthesis of S14 6
3.1.1 Synthetic route to S14 6
3.1.2 Synthesis of S2 6
3.1.3 Synthesis of S3 7
3.1.4 Synthesis of S4 7
3.1.5 Synthesis of S5 8
3.1.6 Synthesis of S6 8
3.1.7 Synthesis of S8 8
3.1.8 Synthesis of S9 9
3.1.9 Synthesis of S10 9
3.1.10 Synthesis of S11 10
3.1.11 Synthesis of S12 10
3.1.12 Synthesis of S13 11
3.1.13 Synthesis of S14 11
3.2 Synthesis of S17 13
3.2.1 Synthetic strategy for S17 13
3.2.2 Synthesis of S15 14
3.2.3 Synthesis of S16 15
3.2.4 Synthesis of S17 16
3.3 Synthesis of control and references 16
3.3.1 Synthesis of S19 16
3.3.2 Synthesis of S20 17
3.3.3 Synthesis of S21 18
3.3.4 Synthesis of S22 18
4 Synthesis of polymers 20
4.1 Representative Procedure for SET-LRP of Methyl Acrylate Using Mechanophore Initiators 20
4.2 Synthesis of mechanophore and control polymers 20
4.2.1 Synthesis of 1_{Pd} 20
4.2.2 Synthesis of 1_{H} 21

4.2.3 Synthesis of S23 21
4.2.4 Synthesis of 2_H 22
4.2.5 Synthesis of 6 23
4.3 SEC Data for Synthesised Polymers 23
4.4 SEC traces for Synthesised Polymers 24
5 Mechanochemical activation via ultrasound 25
5.1 General procedure for sonication experiments 25
5.2 Representative sonication of 1_{Pd} 25
5.3 Representative sonication of 1_H 27
5.4 Representative sonication of S23 29
5.5 Summary of mechanophores activated by sonication 31
6 Computational modelling 32
6.1 General method 32
6.2 CoGEF of 1_{Pd} 33
6.3 CoGEF of 1_H 33
6.4 EFEI of 1_{Pd} and 1_H 35
6.5 Molecular Dynamics Simulations 36
6.5.1 Molecular Dynamics Simulations of 1_{Pd} 36
6.5.2 Molecular Dynamics Simulations of 1_H 45
7 NMR spectra 54
7.1 Small molecule NMR spectra 54
7.1.1 Spectra of S2 54
7.1.2 Spectra of S3 55
7.1.3 Spectra of S4 56
7.1.4 Spectra of S5 57
7.1.5 Spectra of S6 58
7.1.6 Spectra of S8 59
7.1.7 Spectra of S9 60
7.1.8 Spectra of S11 61
7.1.9 Spectra of S13 62
7.1.10 Spectra of S14 63
7.1.11 Synthesis of S15 64
7.1.12 Spectra of S16 65
7.1.13 Spectra of S17 66
7.1.14 Spectra of S19 67
7.1.15 Spectra of S20 68

7.1.16 Spectra of S21 69
7.1.17 Spectra of S22 70
7.2 Polymer NMR spectra 71
7.2.1 Spectra of 1_{Pd} 71
7.2.2 Spectra of 1_{H} 71
7.2.3 Spectra of S23 72
7.2.4 Spectra of 2_{H} 72
7.2.5 Spectra of 6 73
7.3 Post sonication NMR spectra 74
7.3.1 Post-sonication spectra of 1_{Pd} (run 1) 74
7.3.1 Post-sonication spectra of 1_{Pd} (run 2) 75
7.3.2 Post-sonication spectra of 1_{Pd} (run 3) 77
7.3.3 Post-sonication spectra of 1_{H} (run 1) 78
7.3.1 Post-sonication spectra of 1_{H} (run 2) 80
7.3.1 Post-sonication spectra of 1_{H} (run 3) 82
7.3.2 Post-sonication spectra of S23 (run 1) 83
7.3.1 Post-sonication spectra of S23 (run 2) 85
7.3.1 Post-sonication spectra of S24 (run 3) 86
8 Isotopic patterns of rotaxanes S14, S17, and S20 88
8.1 Isotopic distribution of S14 88
8.2 Isotopic distribution of S17 89
8.3 Isotopic pattern of S20 89
9 References 90

2 General Experimental Details

Unless otherwise stated, all reagents and solvents were purchased from commercial suppliers and used without further purification. Dry solvents were obtained by passing through an activated alumina column on a Phoenix SDS solvent drying system (JC Meyer Solvent Systems, CA, USA). Tert-butyl (4-hydroxybenzyl)carbamate,[1] 1-azido-6-bromohexane,[2] **S1**[3], **S7**[4] and **S19**[5] were prepared according to literature procedures.

Size exclusion chromatography (SEC) analyses were performed in DMAc solution containing 0.45 % wt/v LiCl (sample concentration of 1.00 mg mL^{-1}) at 50 °C using a GPC/SEC Agilent 1260 Infinity II with 2 × PL gel 10 µm mixed-C and a PL gel 500 Å column, and equipped with a differential refractive index (DRI) detector employing narrow polydispersity PMMA standards (Agilent Technologies) as a calibration reference. Samples were filtered through a Whatman Puradisc 4 mm syringe filter with 0.45 µm PTFE membrane before injection to equipment, and experiments were carried out with injection volume of 100 µL, flow rate of 0.800 mL min^{-1}. Results were analysed using toluene as internal marker using Agilent GPC/SEC Software Version 2.2.

Ultrasound experiments were performed using a Sonics VCX 500 ultrasonic processor equipped with a 13 mm diameter replaceable-tip probe. The distance between the titanium tip and the bottom of the Suslick cell was 2 cm. The ultrasonic intensity was calibrated using the method outlined by Hickenboth *et al.*[6] The Suslick cells were fabricated by the Department of Chemistry glass workshop at the University of Manchester.

Analytical TLC was performed on precoated silica gel plates (0.25 mm thick, 60 F254, Merck, Germany) and observed under UV light or stained with a potassium permanganate base solution. Preparative TLC was performed on precoated silica gel plates: 500 µm or 2000 µm, UNIPLATE GF, Analtech Inc., DE, USA. Flash column chromatography was performed with silica gel 60 (230-400 mesh) from Sigma-Aldrich.

^{1}H and ^{13}C NMR spectra were recorded on a Bruker Avance III 500 MHz Prodigy instrument or a Bruker Avance III 400 MHz Prodigy instrument. Chemical shifts are reported in parts per million (ppm) from high to low frequency and referenced to the residual solvent resonance. Coupling constants (J) are reported in Hertz (Hz) and splitting patterns are designated as follows: b = broad, s = singlet, d = doublet, t = triplet, q = quartet, p = pentet and m = multiplet. ^{1}H and ^{13}C assignments were made using 1D or 2D NMR methods (HSQC, HMBC, COSY). Mass spectra were obtained through the Mass Spectrometry services in the Department of Chemistry at the University of Manchester.

Abbreviations: CoGEF: constrained geometries simulate external force; DCM: dichloromethane; DMSO: dimethylsulfoxide; ESI: electrospray ionization; HRMS: high resolution mass spectrometry; MA: methyl acrylate; MS: mass spectrometry; Me_6TREN: tris[2-(dimethylamino)ethyl]amine; PE: petroleum ether; THF: tetrahydrofuran; TLC: thin layer chromatography; DMAc: Dimethylacetamide; DMF: Dimethylformamide; EDTA: Ethylenediaminetetraacetic acid; PMMA: Poly(methyl methacrylate); PMA: Poly(methyl acrylate).

3 Synthesis of mechanophore, control and reference compounds

3.1 Synthesis of S14

3.1.1 Synthetic route to S14

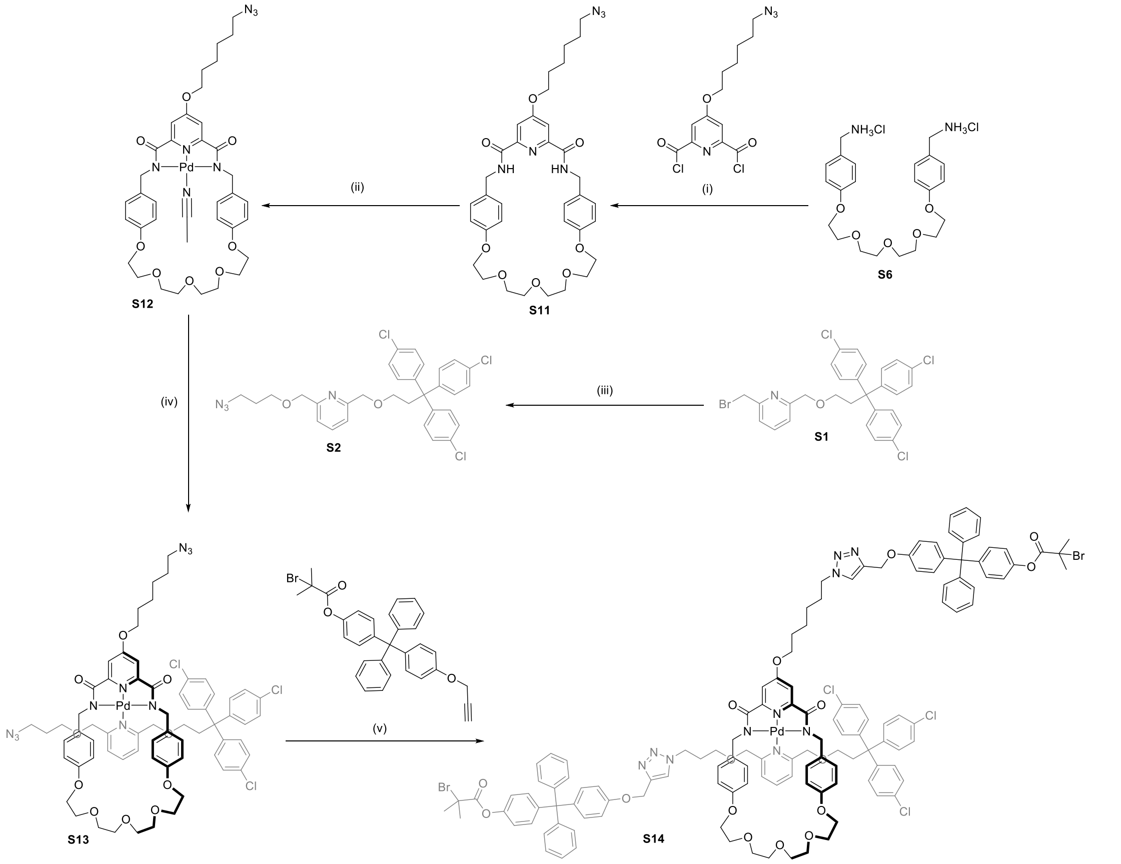

Scheme S1. Synthetic route to **S15**. Conditions: (i) NEt_3, DCM, 0 °C-r.t. (ii) $Pd(OAc)_2$, MeCN, r.t. (iii) 3-azido-1-propanol, NaH, THF, 0 °C-r.t. (iv) DCM:MeCN (10:1), r.t. (v) $CuSO_4$, sodium ascorbate, THF:H_2O (3:1), r.t.

3.1.2 Synthesis of S2

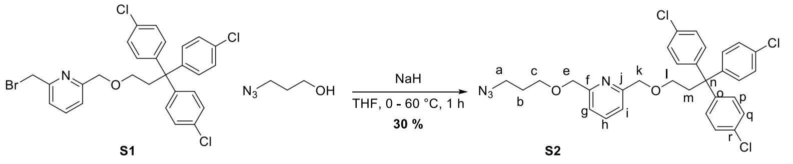

To a 0 °C solution of 3-azido-1-propanol (0.044 g, 0.437 mmol) in THF (1.5 mL) was added NaH (60 % wt in mineral oil, 0.020 g, 0.500 mmol, 1.1 eq.) the suspension was stirred at 0 °C for 30 min before **S1** (0.252 g, 0.437 mmol, 1 eq.) in THF (0.500 mL) was added drop wise. The suspension was warmed to room temperature then stirred at 60 °C for 1 h. The suspension was filtered and concentrated, and the crude purified by SiO_2 column chromatography, eluting with 10-20 % EtOAc in PE to yield **S2** as a white solid (0.079 g, 30 %).

^{1}H NMR (500 MHz, CDCl$_3$) δ: 7.68 (t, *J* = 7.7 Hz, 1H, H$_h$), 7.32 (d, *J* = 7.7 Hz, 1H, H$_g$), 7.23 (d, *J* = 8.7 Hz, 4H, H$_p$), 7.14 (d, *J* = 8.7 Hz, 5H, H$_{q,i}$), 4.59 (s, 2H, H$_e$), 4.44 (s, 2H, H$_k$), 3.63 (t, *J* = 6.0 Hz, 2H, H$_c$), 3.44 (t, *J* = 6.0 Hz, 2H, H$_a$), 3.31 (t, *J* = 7.2 Hz, 2H, H$_l$), 2.92 (t, *J* = 7.2 Hz, 2H, H$_m$), 1.94 (p, *J* = 6.8 Hz, 2H, H$_b$).

^{13}C NMR (126 MHz, CDCl$_3$) δ: 158.00 (C$_f$), 157.66 (C$_j$), 144.73 (C$_o$), 137.38 (C$_h$), 132.51 (C$_r$), 130.21 (C$_q$), 128.47 (C$_p$), 120.20 (C$_i$), 120.14 (C$_g$), 74.07 (C$_k$), 73.99 (C$_e$), 68.36 (C$_l$), 67.74 (C$_c$), 54.24 (C$_n$), 48.59 (C$_a$), 39.94 (C$_m$), 29.31 (H$_b$).

HRMS ESI (+): [M+H]$^+$ calculated for $C_{31}H_{30}O_2N_4Cl_3$ = 595.1429, found: 595.1416

3.1.3 Synthesis of S3

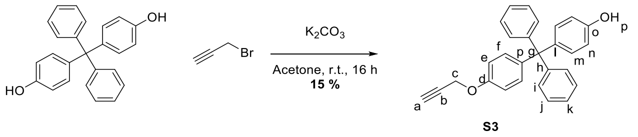

To a suspension of 4,4'-dihydroxytetraphenylmethane (0.500 g, 1.42 mmol) and potassium carbonate (0.382 g, 2.84 mmol, 2 eq.) in acetone (7 mL) was added propargyl bromide (80 % wt. in toluene, 0.300 mL, 2.70 mmol, 1.9 eq.) and the mixture stirred at room temperature overnight. The suspension was filtered, concentrated and purified by SiO_2 column chromatography, eluting with 5:1 PE:EtOAc yielding **S3** (0.081 g, 15 %).

^{1}H NMR (500 MHz, CDCl$_3$) δ: 7.35 – 7.28 (m, 4H, C$_j$), 7.28 – 7.21 (m, 6H, H$_k$ and H$_i$), 7.18 (d, *J* = 9.0 Hz, 2H, H$_e$), 7.11 (d, *J* = 8.8 Hz, 2H, H$_n$), 6.92 (d, *J* = 9.0 Hz, 2H, H$_f$), 6.77 (d, *J* = 8.8 Hz, 2H, H$_m$), 5.00 (s, 1H, H$_p$), 4.73 (d, *J* = 2.4 Hz, 2H, H$_c$), 2.59 (t, *J* = 2.4 Hz, 1H, H$_a$).

^{13}C NMR (126 MHz, CDCl$_3$) δ: 155.68 (C$_d$), 153.58 (C$_o$), 147.25 (C$_h$), 140.26 (C$_p$), 139.44 (C$_l$), 132.47 (C$_m$), 132.28 (C$_f$), 131.17 (C$_i$), 127.54 (C$_{j,}$ or C$_k$), 126.00 (C$_j$ or C$_k$), 114.36 (C$_n$), 113.72 (C$_e$), 78.81 (C$_b$), 75.64 (C$_a$), 63.76 (C$_g$), 55.93 (C$_c$).

HRMS ESI (-): [M-H]$^-$ Calculated for $C_{28}H_{21}O_2^-$ = 389.15415, found: 389.1556

3.1.4 Synthesis of S4

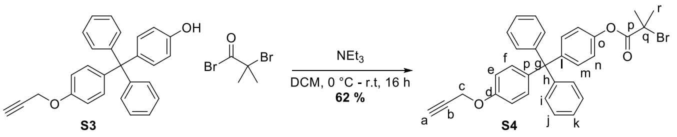

To a 0 °C solution of **S3** (0.081 g, 0.207 mmol) and NEt_3 (0.050 mL, 0.037 g, 0.360 mmol, 1.7 eq.) in DCM (1 mL) was added 2-bromoisobutyryl bromide (0.030 mL, 0.059 g, 0.242 mmol, 1.2 eq.) dropwise and the solution stirred at room temperature overnight. The solvent was removed and the crude purified by SiO_2 column chromatography, eluting with 5 % EtOAc in PE yielding **S4** as a white solid (0.069 g, 62 %).

^{1}H NMR (500 MHz, CDCl$_3$) δ: 7.30 – 7.15 (m, 12H, H$_{m,h,i,j,k}$), 7.12 (d, *J* = 8.9 H$_z$, H$_f$), 7.03 (d, *J* = 8.8 Hz, 2H, H$_n$), 6.86 (d, *J* = 8.9 Hz, 2H, H$_e$), 4.67 (d, *J* = 2.4 Hz, 2H, H$_c$), 2.52 (t, *J* = 2.4 Hz, 1H, H$_a$), 2.06 (s, 6H, H$_r$).

^{13}C NMR (126 MHz, CDCl$_3$) δ: 170.38 (C$_p$), 155.85 (C$_d$), 148.84 (C$_o$), 146.79 (C$_h$), 145.06 (C$_p$), 139.77 (C$_l$), 132.28 (C$_{f,m}$), 131.16 (C$_k$), 127.70 (C$_j$), 126.20 (C$_i$), 119.98 (C$_n$), 113.87 (C$_e$), 78.77 (C$_b$), 75.65 (C$_a$), 64.11 (C$_g$), 55.95 (C$_c$), 55.54 (C$_p$), 30.77 (C$_r$).

HRMS ESI (+): [M+Na]$^+$ Calculated for $C_{32}H_{27}BrO_3Na$ = 561.104126, found: 561.1060

3.1.5 Synthesis of S5

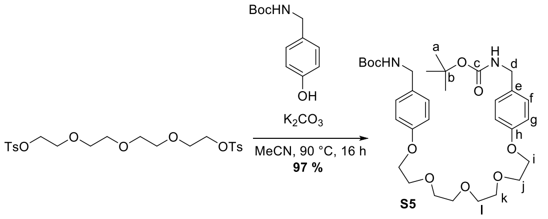

A suspension of tetraethelyene glycol ditosylate (0.940 g, 1.87 mmol), potassium carbonate (0.520 g, 3.76 mmol, 2 eq.) and tert-butyl (4-hydroxybenzyl)carbamate (0.840 g, 3.76 mmol, 2 eq.) in MeCN (10 mL) was stirred at 90 °C overnight. The solvent was removed and the residue re-dissolved in EtOAc, washed once with H_2O and 3 times with 1 M NaOH, dried over $MgSO_4$, filtered and concentrated to yield **S5** as a white solid which was used without further purification (1.092 g, 97 %).

^{1}H NMR (400 MHz, CDCl$_3$) δ: 7.17 (d, *J* = 8.3 Hz, 4H, H_f), 6.85 (d, *J* = 8.3 Hz, 4H, H_g), 4.80 (bs, 2H, NH), 4.22 (d, *J* = 5.3 Hz, 4H, H_d), 4.09 (t, *J* = 5.1, 4H, H_i), 3.84 (t, *J* = 5.1, 4H, H_j), 3.76 – 3.62 (m, 8H, $H_{k\text{-}l}$), 1.45 (s, 18H, H_a).

^{13}C NMR (101 MHz, CDCl$_3$) δ: 158.24 (C_h), 155.99 (C_c), 131.35 (C_e), 128.92 (C_f), 114.84 (C_g), 79.53 (C_b), (70.96, 70.81) ($C_{k\text{-}l}$), 69.86 (C_j), 67.61 (C_i), 44.29 (C_d), 28.55 (C_a).

HRMS ESI (+): $[M+H]^+$ Calculated for $C_{32}H_{49}O_9N_2$ =, 605.3433 found: 605.3438

3.1.6 Synthesis of S6

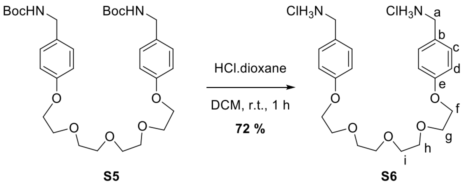

To a solution of **S5** (1.092 g, 1.81 mmol) in DCM (9 mL) was added HCl (4 N in dioxane, 9 mL, 36.11 mmol, 20 eq.) and the solution stirred at room temperature for 1 h. The solvent was removed and **S6** dried on high vac and used without further purification (0.620 g, 72 %).

^{1}H NMR (400 MHz, MeOD) δ: 7.38 (d, *J* = 8.7 Hz, 4H, H_c), 7.00 (d, *J* = 8.7 Hz, 4H, H_d), 4.16 – 4.09 (m, 4H, H_f), 4.04 (s, 4H, H_a), 3.86 – 3.80 (m, 4H, H_g), 3.72 – 3.67 (m, 8H, $H_{h\text{-}i}$).

^{13}C NMR (101 MHz, MeOD) δ: 160.99 (C_e), 131.63 (C_b), 126.53 (C_c), 116.16 (C_d), (71.74, 71.58)($C_{h\text{-}i}$), 70.75 (C_g), 68.71 (C_f), 43.89 (C_a).

HRMS ESI (+): $[M+Na]^+$ Calculated for $C_{22}H_{32}N_2O_5Na$ =, 427.2209 found: 427.2224

3.1.7 Synthesis of S8

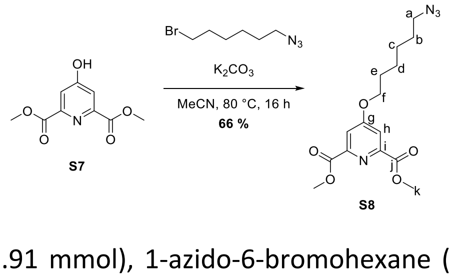

A suspension of **S7** (0.404 g, 1.91 mmol), 1-azido-6-bromohexane (1.46 g, 1.91 mmol, 1 eq.), and potassium carbonate (0.527 g, 3.8 mmol, 2 eq.) in MeCN (4 mL) was stirred at 80 °C overnight. The

mixture was cooled to room temperature, poured onto H_2O, extracted 3 times with EtOAc, washed with 5 % wt LiCl and brine, dried over $MgSO_4$, filtered, and concentrated. The crude was purified by SiO_2 column chromatography eluting with 40 % EtOAc in PE to yield **S8** as a white solid (0.423 g, 66 %).

^{1}H NMR (400 MHz, $CDCl_3$) δ: 7.79 (s, 2H, H_h), 4.14 (t, *J* = 6.3 Hz, 2H, H_f), 4.00 (s, 6H, H_k), 3.29 (t, *J* = 6.8 Hz, 2H, H_a), 1.86 (p, *J* = 6.3 Hz, 2H, H_e), 1.64 (p, *J* = 6.8 Hz 2H, H_b), 1.57 – 1.41 (m, 4H, $H_{c\text{-}d}$).

^{13}C NMR (101 MHz, $CDCl_3$) δ: 167.18 (C_g), 165.34 (C_j), 149.89 (C_i), 114.65 (C_h), 68.96 (C_f), 53.38 (C_k), 51.44 (C_a), 28.89 C($_e$), 28.75 (C_b), (26.53, 25.62)($C_{c\text{-}d}$).

HRMS ESI (+): $[M+Na]^+$ Calculated for $C_{15}H_{21}O_5N_4$ = 337.1506, found: 337.1495

3.1.8 Synthesis of S9

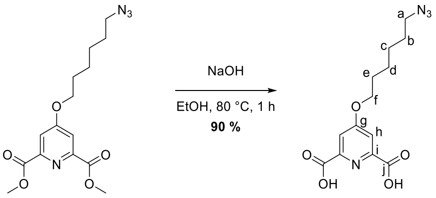

S8 S9

A solution of **S8** (1.644 g, 4.89 mmol) in EtOH (24 mL) and NaOH (1 M in H_2O, 15 mL, 15 mmol, 3 eq.) was stirred at 80 °C for 1 h. The mixture was cooled to room temperature, concentrated and the crude re-dissolved in H_2O, acidified with 1 M HCl and extracted 3 times with EtOAC. The organics were dried over $MgSO_4$, filtered, and concentrated to yield **S9** as a white solid (1.361 g, 90 %).

^{1}H NMR (400 MHz, DMSO-d_6) δ: 7.69 (s, 2H, H_h), 4.21 (t, *J* = 6.4 Hz, 2H, H_f), 3.32 (t, *J* = 6.8 Hz, 2H, H_a), 1.76 (p, *J* = 604 Hz, 2H, H_e), 1.55 (p, *J* = 6.8 Hz, 2H, H_b), 1.49 – 1.34 (m, 4H, $H_{c,d}$).

^{13}C NMR (101 MHz, DMSO-d_6) δ: 166.77 (C_g), 165.34 (C_j), 149.74 (C_i), 113.57 (C_h), 68.65 (C_f), 50.58 (C_a), 28.17 (C_e), 28.05 (C_b), (25.80, 24.84)($C_{c\text{-}d}$).

HRMS ESI (+): $[M-H]^-$ Calculated for $C_{13}H_{15}N_4O_5$ = 307.1042, found: 307.1064

3.1.9 Synthesis of S10

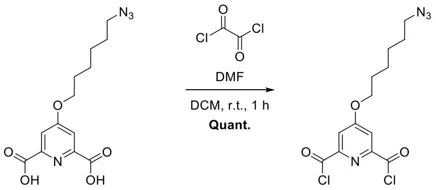

S9 S10

To a room temperature suspension of **S9** (0.090 g, 0.0292 mmol) in DCM was added oxalyl chloride (0.080 mL, 0.946 mmol, 3.2 eq.) and 1 drop of DMF. The suspension was stirred at room temperature for 1 h, or until complete dissolution, then concentrated to dryness. **S10** was dried on high vacuum for 1 h and used directly for macrocycle formations (0.100 g, quant.).

3.1.10 Synthesis of S11

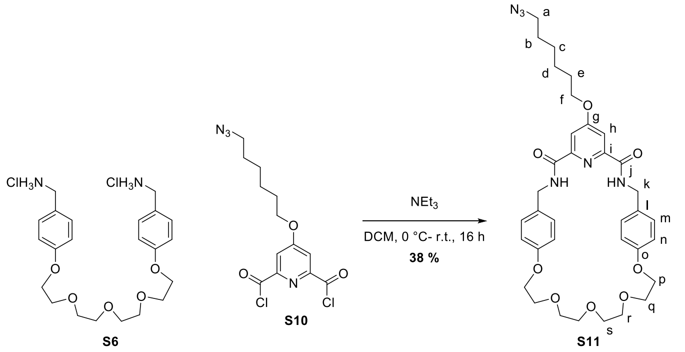

To a 0 °C solution of **S6** (0.118 g, 0.246 mmol) and NEt_3 (0.140 mL, 0.102 g, 1.01 mmol, 4.1 eq.) in DCM (100 mL), was added a solution of **S10** (0.085 g, 0.246 mmol, 1 eq.) in DCM (20 mL) over 1 h. The reaction was stirred at room temperature overnight, concentrated and the crude purified by SiO_2 column chromatography, eluting with 20 % MeCN in DCM to yield **S11** as a white solid (0.064 g, 38 %).

^{1}H NMR (400 MHz, MeCN-d$_3$) δ: 8.87 (t, *J* = 6.4 Hz, 2H, NH), 7.63 (s, 2H, H_h), 7.12 (d, *J* = 8.7 Hz, 4H, H_m), 6.72 (d, *J* = 8.6 Hz, 4H, H_n), 4.51 (d, *J* = 6.4 Hz, 4H, H_k), 4.12 (t, *J* = 6.5 Hz, 2H, H_f), 4.00 – 3.94 (m, 4H, H_p), 3.75 – 3.69 (m, 4H, H_q), 3.64 – 3.52 (m, 8H, $H_{r,s}$), 3.29 (t, *J* = 6.9 Hz, 2H, H_a), 1.77 (p, *J* = 6.5 Hz, 2H, H_e), 1.59 (p, *J* = 7.0 Hz, 2H, H_b), 1.51 – 1.34 (m, 4H, $H_{c,d}$)

^{13}C NMR (101 MHz, MeCN-d$_3$) δ: 168.78 (C_g), 164.19 (C_j), 158.85 (C_o), 151.79 (C_i), 132.30 (C_l), 129.69 (C_n), 115.06 (C_m), 111.64 (C_h), 71.33 ($C_{r,s}$), 70.23 (C_q), 69.68 (C_f), 68.35 (C_p), 52.02 (C_a), 42.66 (C_k), 29.36 (C_e), 27.03 (C_b), 26.08 ($C_{c,d}$).

HRMS ESI (+): $[M+Na]^+$ Calculated for $C_{35}H_{44}N_6O_8Na$ = 699.3118, found: 699.3127

3.1.11 Synthesis of S12

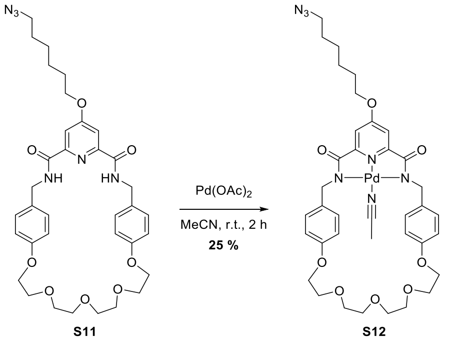

To a room temperature solution of **S11** (0.099 g, 0.146 mmol) in MeCN (3.7 mL) was added $Pd(OAc)_2$ (0.036 g, 0.160 mmol, 1.1 eq.) and the resulting suspension stirred for 2 h at room temperature. The precipitate was filtered and dried under high vacuum, **S12** was directly used in the next step without further purification (0.030 g, 25 %).

3.1.12 Synthesis of S13

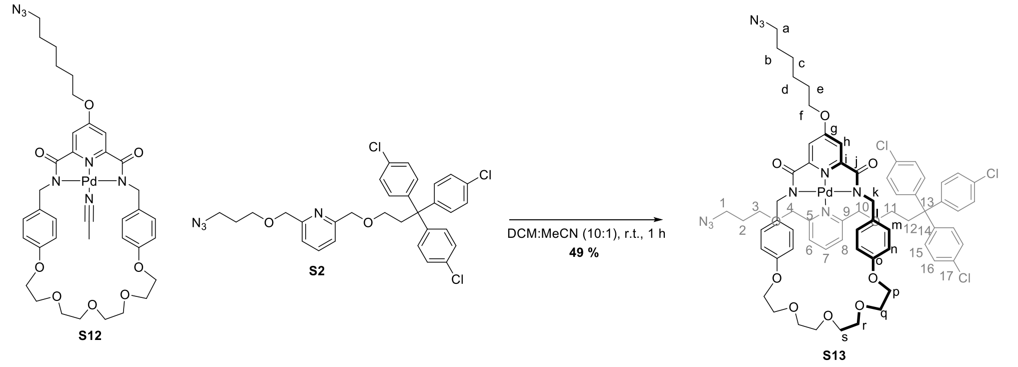

A solution of **S12** (0.030 g, 0.037 mmol) and **S2** (0.021 g, 0.037 mmol) in DCM:MeCN (10:1, 0.800 mL) was stirred for 1 h at room temperature. The solvent was removed and the crude purified by SiO_2 column chromatography, eluting with 20 % MeCN in DCM to yield **S13** as a yellow solid (0.024 g, 49 %).

^{1}H NMR (400 MHz, Acetone-d$_6$) δ: 8.08 (t, *J* = 7.8 Hz, 1H, H_7), 7.50 (d, *J* = 7.7 Hz, 1H, H_8), 7.42 (d, *J* = 7.9 Hz, 1H, H_6), 7.29 (s, 12H, $H_{15,16}$), 7.25 (s, 2H, H_h), 6.49 (d, *J* = 8.5 Hz, 4H, H_m), 6.35 (d, *J* = 8.2 Hz, 4H, H_n), 4.89 (s, 2H, H_{10}), 4.60 (d, *J* = 13.8 Hz, 2H, $H_{k'}$), 4.36 (t, *J* = 6.4 Hz, 2H, H_f), 4.10 – 3.95 (m, 4H, H_p), 3.83 (s, 2H, H_4), 3.75 (t, *J* = 4.4 Hz, 4H, H_q), 3.66 – 3.53 (m, 8H, $H_{r,s}$), 3.50 (t, *J* = 7.4 Hz, 2H, H_{11}), 3.45 (t, *J* = 7.1 Hz, 2H, H_a), 3.36 (m, 4H, H_1 and $H_{k'}$), 3.18 (t, *J* = 6.7 Hz, 2H, H_3), 3.04 (t, *J* = 7.4 Hz, 2H, H_{12}), 1.92 (p, *J* = 6.4 Hz, 2H, H_e), 1.87 (p, *J* = 6.8 Hz, 2H, H_2), 1.66 (p, *J* = 6.9 Hz, 2H, H_b), 1.61 – 1.58 (m, 4H, $H_{c,d}$).

^{13}C NMR (101 MHz, Acetone-d$_6$) δ: 171.45 (C_j), 170.36 (C_g), 163.03 (C_5), 159.61 (C_9), 158.57 (C_o), 155.12 (C_i), 146.05 (C_{14}), 140.24 (C_7), 134.74 (C_l), 132.67 (C_{17}), 131.50 (C_{16}), 129.29 (C_n), 129.06 (C_{15}), 123.37 (C_8), 122.93 (C_6), 114.95 (C_m), 111.32 (C_h), 74.08 (C_{10}), 72.40 (C_4), 71.37 ($C_{r,s}$), 70.59 (C_f), 70.38 (C_q), 69.72 (C_{11}), 69.19 (C_a), 68.39 (C_p), 55.19 (C_{13}), 51.93 (C_1), 49.70 (C_a and C_k), 39.65 (C_{12}), 29.31 (C_2), 29.10 ($C_{b,e}$), ((27.05 and 26.09) (C_c and C_d).

HRMS ESI (+): $[M+Na]^+$ Calculated for $C_{66}H_{71}Cl_3N_{10}O_{10}PdNa$ = 1397.3353, found: 1397.3332

3.1.13 Synthesis of S14

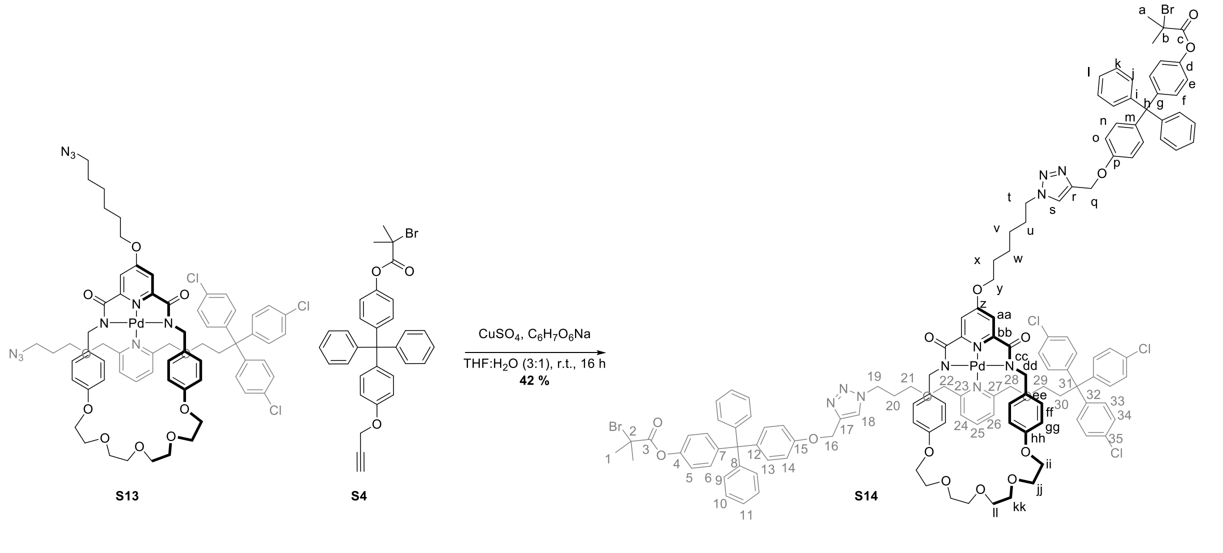

To a solution of **S13** (0.020 g, 0.015 mmol) and **S4** (0.016 g, 0.030 mmol, 2 eq.) in THF:H_2O (3:1, degassed for 20 min by bubbling N_2 prior to use, 0.150 mL) was added 50 mM aqueous solutions of $CuSO_4$ (0.060 mL, 0.003 mmol, 0.2 eq.) followed by sodium ascorbate (0.120 mL, 0.006 mmol, 0.4 eq.)

and the resulting solution stirred at room temperature overnight. The solution was diluted with DCM, washed with 0.25 M EDTA solution, dried over $MgSO_4$, filtered and concentrated. The crude was purified by preparative TLC, eluting with 30 % MeCN in DCM, yielding **S14** as a yellow solid (0.017 g, 42 %).

^{1}H NMR (500 MHz, Acetone-d$_6$) δ: 8.16 (s, 1H, H_{18},or H_s), 8.12 – 8.02 (m, 2H, H_{25} and H_{18} or H_s), 7.54 (d, *J* = 7.7 Hz, 1H, H_{26}), 7.33 –7.10 (m, 51H, $H_{5,6,9,10,11,13,14,33,34,e,f,j,k,l,n,24,6,aa}$), 7.00-6.95 (m, 4H, H_{14} or H_5 or H_e or H_o), 6.41 (d, *J* = 8.2 Hz, 4H, H_{ff}), 6.26 (d, *J* = 8.0 Hz, 4H, H_{gg}), 5.18 (s, 2H, H_{16} or H_q), 5.16 (s, 2H, H_{16} or H_q), 4.98 (s, 2H, H_{28}), 4.82 (d, *J* = 13.6 Hz, 2H, $H_{dd'}$), 4.44 (m, 4H, H_{19} and H_t), 4.29 (t, *J* = 6.4 Hz, 2H, H_y), 4.01-3.50 (m, 18H, $H_{ii\text{-}ll}$ and H_{22}), 3.15 (d, *J* = 13.7 Hz, 2H, $H_{dd'}$), 3.11-3.05 (m, 4H, H_{29} and H_{30}), 2.18 (p, *J* = 7.2 Hz, 2H, H_{20}), 2.06 (s, 6H, H_1 or H_a), 2.05 (s, 6H, H_1 or H_a), 1.96 (p, *J* = 7.4 Hz, 2H, H_x), 1.85 (p, *J* = 6.7 Hz, 2H, H_u), 1.56 (p, *J* = 7.6 Hz, 2H, H_v or H_w), 1.42 (p, *J* = 7.0 Hz, 2H, H_v or H_w).

^{13}C NMR (126 MHz, Acetone-d$_6$) δ: 171.43 (C_{cc}), 170.55 (C_3 and C_c), 170.36 (C_z), 163.75 (C_{23}), 158.74 (C_{27}), 158.57 (C_{hh}), (157.72, 157.70 ($C_{15,4,p,d}$)), 155.08 (C_{bb}), 149.84 (C_{12} or C_g), 147.77 (C_{17}), 146.03 (C_{12} or C_g), 145.95, 144.17 (C_{25}), 139.77 ($C_{7,17,m,r}$), 134.78 (C_{ee}), 132.85 (C_{35}), 132.67 (C_{34}), 131.71 (C_{33}), 131.69 (C_{32}), 131.45, 129.40 (C_{ff}), (129.08, 128.49, 126.94, 126.92, 124.89, 124.54)($C_{9\text{-}11,j\text{-}l}$), 123.50 ($C_{26}$), 123.04 ($C_{24}$), 120.97 ($C_{6,13,f,n}$) 114.92 ($C_{gg}$), (114.67,114.57 ($C_{14}$,$C_o$), 111.38 ($C_{aa}$), 74.43 ($C_{28}$), 72.33 ($C_{22}$), 71.45 ($C_{kk,ll}$), 70.52 ($C_y$), 69.80 ($C_{29}$), 69.33, 68.35 ($C_{ii\text{-}jj}$), 64.79 ($C_h$), 62.47 ($C_{16}$,$C_q$), (57.13, 55.16) ($C_{2,b}$), 50.55 ($C_{31}$), 49.74 ($C_{dd}$), 48.31, 39.79 ($C_{30}$), 30.87 ($C_{20}$), 30.79 ($C_{1,a}$), 29.21 ($C_x$), 29.10 ($C_u$), 26.69 ($C_v$), 25.89 ($C_w$).

HRMS ESI (+): $[M+Na]^+$ Calculated for = $C_{130}H_{125}Br_2Cl_3N_{10}O_{16}PdNa$, 2473.5634 found: 2473.5647, for isotopic pattern see section 8.1

3.2 Synthesis of S17

3.2.1 Synthetic strategy for S17

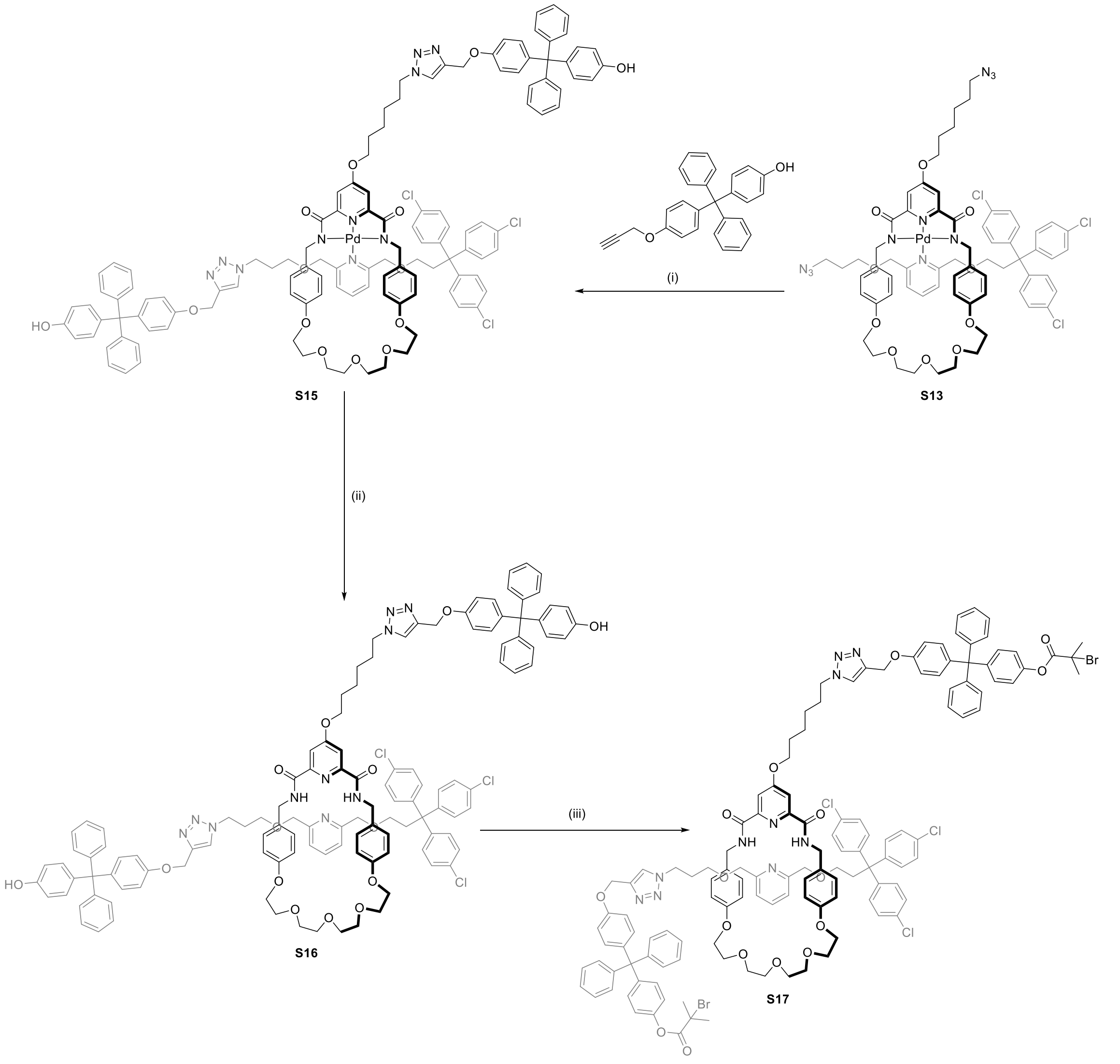

Scheme S2. Synthetic route to de-metalated rotaxane. Conditions: (i) $CuSO_4$, sodium ascorbate, THF:H_2O (3:1), r.t. (ii) KCN, DCM:MeOH (1:1), r.t. (iii) 2-bromoisobutyryl bromide, NEt_3, DCM, 0 °C-r.t.

3.2.2 Synthesis of S15

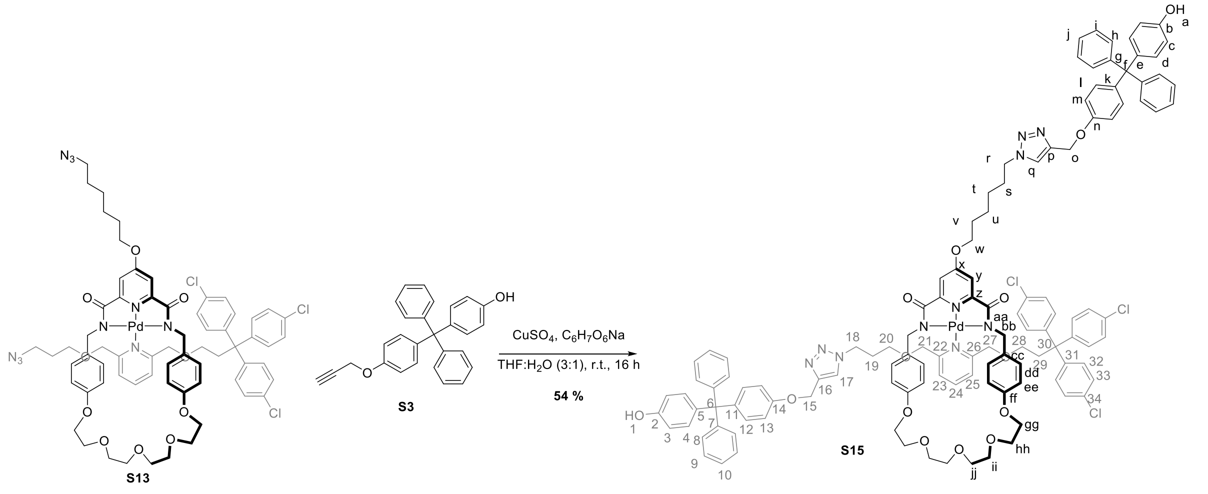

To a solution of **S13** (0.027 g, 0.020 mmol) and **S3** (0.016 g, 0.040 mmol, 2 eq.) in THF:H_2O (3:1, degassed for 20 min by bubbling N_2 prior to use, 0.080 mL) was added 50 mM aqueous solutions of $CuSO_4$ (0.040 mL, 0.002 mmol, 0.1 eq.) followed by sodium ascorbate (0.080 mL, 0.004 mmol, 0.2 eq.) and the resulting solution stirred at room temperature overnight. The solution was diluted with DCM, washed with 0.25 M EDTA solution, dried over $MgSO_4$, filtered and concentrated. The crude was purified by SiO_2 column chromatography, eluting with 20 % MeCN in DCM, yielding **S15** as a yellow solid (0.023 g, 54 %).

^{1}H NMR (400 MHz, methylene chloride-d$_2$) δ: 8.47 (s, 1H, H_1 or H_a), 8.22 (s, 1H, H_1or H_a), 7.98 (s, 1H, H_q or H_{17}), 7.88 (t, *J* = 7.9 Hz, 1H, H_{24}), 7.55 (s, 1H, H_q or H_{17}), 7.41 (d, *J* = 7.7 Hz, 1H, H_{23} or H_{25}), 7.29 (s, 2H, H_y), 7.27 – 7.06 (m, 20H, $H_{8\text{-}10,g\text{-}j}$), 7.02-6.70 (m, 16H, $H_{3,4,13,12,d,c,l,m}$), 6.31 (d, *J* = 8.3 Hz, 4H, H_{dd}), 6.23 (d, *J* = 8.3 Hz, 4H, H_{ee}), 5.25 (s, 2H, H_{15} or H_o), 5.24 (s, 2H, H_{15} or H_o), 5.14 (d, *J* = 14 Hz, 2H , $H_{bb'}$), 5.10 (s, 2H, H_{27}), 4.34 (t, *J* = 7.4 Hz, 2H, H_{18} or H_r), 4.29 (t, *J* = 7.0 Hz, 2H, H_{18} or H_r), 4.05 (t, *J* = 6.2 Hz, 2H, H_w), 3.90– 3.46 (m, 20H, $H_{gg\text{-}jj,y,20}$), 3.16 (s, 2H, H_{21}), 3.02 (t, *J* = 7.1 Hz, 2H, H_{28}), 2.95 (t, *J* = 7.1 Hz, 2H, H_{29}), 2.76 (d, *J* = 14.0 Hz, 2H, $H_{bb'}$), 2.10 – 1.96 (m, 2H, H_v), 1.88 (p, *J* = 7.8 Hz, 2H, H_{19}), 1.75 (t, *J* = 7.7 Hz, 2H, H_s), 1.46 (p, *J* = 7.8 Hz, 1H, H_u), 1.33 – 1.28 (m, 2H, H_t).

^{13}C NMR (101 MHz, methylene chloride-d$_2$) δ: 171.33 ($C_{aa,x}$), 169.94 (C_z), 164.25 (C_{22} or C_{26}), 157.96 (C_{ff}), 157.54 (C_{22} or C_{26}), 156.56, 156.47 (C_{14} and C_n), 155.50, 154.44 (C_b and C_2), 147.85-147.75 ($C_{6,7,11,k,e,g}$), 145.04 (C_{33}), 144.41-140.71 ($C_{16,p,cc}$), 140.37, 139.30 (C_{24}), (138.41, 138.26, 133.24, 132.62, 132.43, 132.31, 131.30, 130.59)($C_{31\text{-}34,7\text{-}10,4,12,d,l}$), 129.08 ($C_{dd}$), (128.71, 127.75, 127.68, 126.10, 126.06)($C_{7\text{-}10,g\text{-}j}$), 124.11 ($C_q$ or C_{17}), 123.16 (C_q or C_{17}), 122.25 (C_{23} and C_{25}), (114.85, 114.30, 114.14, 113.85)($C_{3,13,c,m,ee}$), 111.40 (C_y), (75.63, 73.85) ($C_{21,27,15,o}$), (71.38, 71.22, 71.03, 70.25, 70.11, 69.57, 68.88, 67.72)($C_{gg\text{-}jj,w,20,28}$), (63.99, 63.91)($C_{31,f}$), 54.60 (C_{30}), 50.61 (C_r or C_{18}), 49.82 (C_{bb}), 48.27 (C_r or C_{18}), 39.76 (C_{29}), (30.45, 30.00, 28.73, 26.40, 25.62)(C_{19},$C_{s\text{-}v}$).

HRMS ESI (+): $[M+Na]^+$ calculated for $C_{122}H_{115}Cl_3N_{10}O_{14}PdNa^+$ = 2177.6592, found: 2177.6596

3.2.3 Synthesis of S16

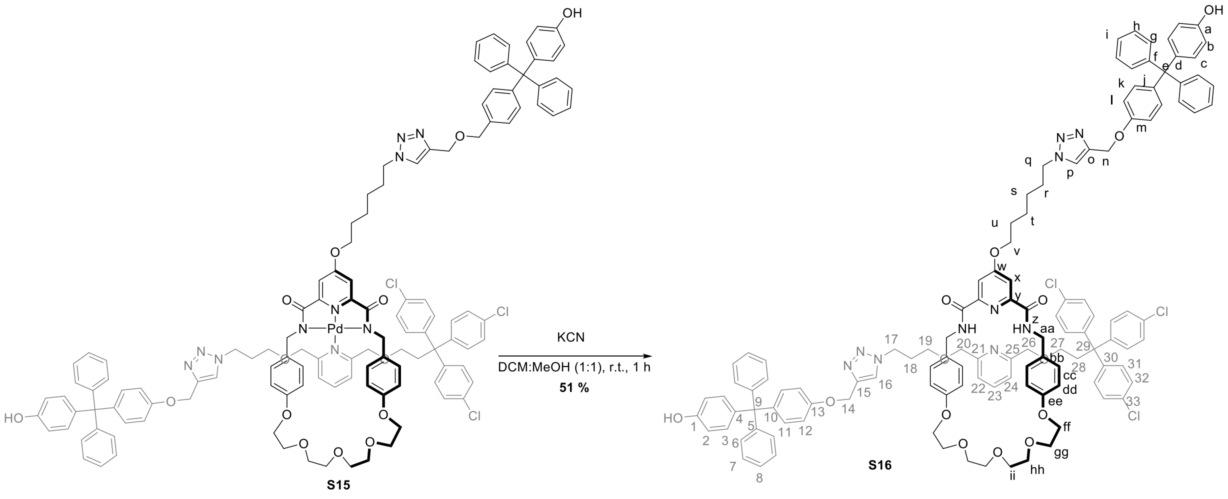

To a solution of **S15** (0.037 g, 0.017 mmol) in DCM:MeOH (1:1, 0.430 mL) was added KCN (0.017 g, 0.261 mmol, 15 eq.) and the solution stirred at room temperature for 1 h. The mixture was diluted with DCM, poured onto H_2O and extracted 3 times with DCM, dried over $MgSO_4$, filtered and concentrated. The crude was purified by preparative TLC, eluting with 3 % MeOH in DCM to yield **S16** as a white solid (0.018 g, 51 %).

^{1}H NMR (400 MHz, acetone-d$_6$) δ: 9.43 (t, *J* = 6.4 Hz, 2H, NH), 8.42 (bs, 2H, OH), 8.06 (s, 1H, H_{16} or H_p), 7.90 (s, 1H, H_{16} or H_p), 7.82 (s, 2H, H_x), 7.74 (t, *J* = 7.7 Hz, 1H, H_{23}), 7.30 (d, *J* = 8.7 Hz, 6H, H_{32}), 7.28 – 7.18 (m, 22H, $H_{6-8,22,24,g-i}$), 7.16 (d, *J* = 8.7 Hz, 6H, H_{31}), 7.09 (d, *J* = 9.0 Hz, 2H, H_{11} or H_k), 7.04 (d, *J* = 8.9 Hz, 2H, H_{11} or H_k), 7.00 – 6.95 (m, 4H, H_3 and H_c), 6.94 (d, *J* = 9.0 Hz, 2H, H_{12} or H_l), 6.87 (d, *J* = 8.9 Hz, 2H, H_{12} or H_l), 6.77 – 6.72 (m, 4H, $H_{2,b}$), 6.66 (d, *J* = 8.6 Hz, 2H, H_{cc}), 6.43 (d, *J* = 8.6 Hz, 2H, H_{dd}), 5.15 (s, 2H, H_{14} or H_n), 4.96 (s, 2H, H_{14} or H_n), 4.54 – 4.38 (m, 4H, $H_{aa',q}$), 4.25 – 4.16 (m, 4H, $H_{17,v}$), 4.15 (s, 2H, H_{20} or H_{26}), 4.11 – 4.04 (m, 2H, $H_{aa'}$), 3.96 – 3.89 (m, 6H, H_{20} or H_{26}, and H_{ff}), 3.73 – 3.66 (m, 4H, H_{gg}), 3.62 – 3.45 (m, 8H, $H_{hh,ii}$), 3.15 (m, 4H, $H_{19,27}$), 2.78 (t, *J* = 7.2 Hz, 2H, H_{28}), 1.97 – 1.87 (m, 4H, $H_{u,18}$), 1.83 (p, *J* = 6.7 Hz, 2H, H_r), 1.65 – 1.49 (m, 2H, H_t), 1.41 (m, 2H, H_s).

^{13}C NMR (101 MHz, acetone-d$_6$) δ: 168.54 (C_w), 163.79 (C_z), 159.12 (C_{21} or C_{25}), 158.64 (C_{ee}), 158.02 (C_{21} or C_{25}), (157.49, 157.34)($C_{13,m}$), (156.31, 156.26)($C_{1,a}$), 152.36 (C_y), 148.41 ($C_{8,i}$), 148.37 (C_{33}), 145.95, (144.23, 144.14) ($C_{15,o}$), (140.41, 140.27)($C_{10,j}$), (138.73, 138.69)($C_{d,4}$), 138.17 (C_{23}), (132.88, 132.84)($C_{3,c}$), (132.73, 132.62)($C_{12,l}$), 131.80 (C_3, C_{bb}), 131.75 (C_{30}), 131.72, 131.25 (C_{31}), 129.73 (C_{cc}), 129.05 (C_{32}), (128.23, 126.66, 126.61)($C_{6-8,g-i}$), (124.82, 124.48)($C_{16,p}$), (120.72, 120.63)($C_{22,24}$), (115.07, 114.95)(C_2,C_b, C_{dd}), (114.37, 114.34)($C_{11,k}$), 111.68 (C_x), (73.62, 73.26)($C_{20,26}$), (71.39, 71.30, 70.18, 69.41)(C_{ff-ii}) (C_v), 68.93 (C_{ff}), 68.40 ($C_{19,27}$), 68.34, 64.38, (62.38, 62.13)($C_{14,n}$), 54.82 (C_{29}), 50.54 (C_q), 47.90 (C_{17}), 42.78 (C_{aa}), 39.93 (C_{28}), 32.62, 30.89 (C_{18} and C_u), 26.77 (C_t), 25.98 (C_s)

HRMS ESI (+): $[M+Na]^+$ calculated for $C_{122}H_{117}O_{14}N_{10}Cl_3Na^+$ = 2073.7709, found: 2073.7728

3.2.4 Synthesis of S17

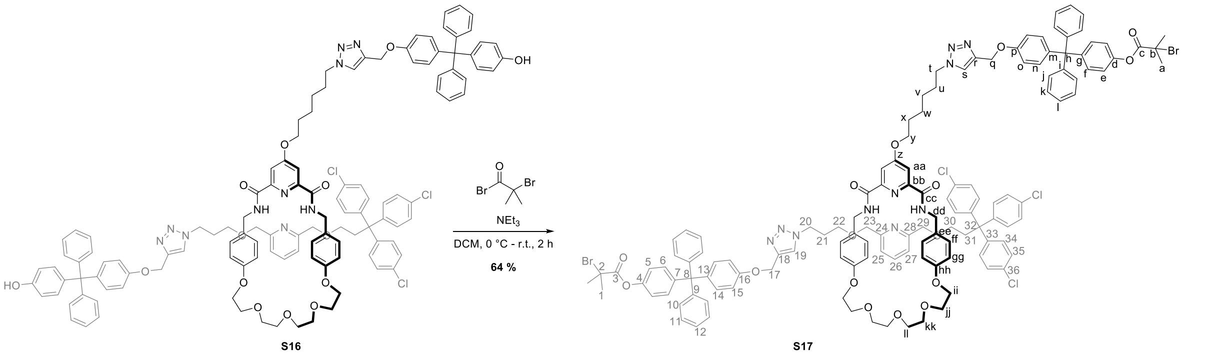

To a 0 °C solution of **S16** (0.011 g, 0.005 mmol) in DCM (0.100 mL) was added 0.2 M DCM solutions of NEt_3 (0.070 mL, 0.014 mmol, 2.6 eq.) and 2-Bromoisobutyryl bromide (0.060 mL, 0.012 mmol, 2.2 eq.) and the solution stirred at room temperature for 2 h. The solvent was removed and the crude purified by preparative TLC, eluting with 2 % MeOH in DCM to yield **S17** as a white solid (0.008 g, 64 %.)

^{1}H NMR (400 MHz, acetone-d$_6$) δ: 9.41 (t, *J* = 6.2 Hz, 2H, NH), 8.06 (s, 1H, H_s or H_{19}), 7.89 (s, 1H, H_s or H_{19}), 7.82 (s, 2H, H_{aa}), 7.74 (t, *J* = 7.7 Hz, 1H, H_{26}), 7.32 – 7.09 (m, 32H, $H_{10\text{-}12,34,35,j\text{-}l}$), 7.06 (d, *J* = 8.9 Hz, 2H, H_e or H_5, or H_o or H_{15}), 6.97 (d, *J* = 9.0 Hz, 2H, H_f or H_n or H_6 or H_{14}), 6.89 (d, *J* = 9.0 Hz, 2H, H_f or H_n or H_6 or H_{14}), 6.67 (d, *J* = 8.6 Hz, 4H, H_{ff}), 6.42 (d, *J* = 8.6 Hz, 4H, H_{gg}), 5.16 (s, 2H, H_q or H_{17}), 4.95 (s, 2H, H_q or H_{17}), 4.53 – 4.39 (m, 4H, H_t and $H_{dd'}$), 4.23-4.16 (m, 4H, H_{20} and H_y), 4.14 (s, 2H, H_{23} or H_{29}), 4.07 (m, 2H, $H_{dd'}$), 3.96 (s, 2H, H_{23} or H_{29}), 3.96 – 3.90 (m, 4H, H_{ii}), 3.72 – 3.66 (m, 4H, H_{jj}), 3.63 – 3.49 (m, 8H, $H_{kk\text{-}ll}$), 3.17 (m, 4H, $H_{30,22}$), 2.79 (d, *J* = 7.4 Hz, 2H, H_{31}), 2.06 (b, 12H, $H_{a,1}$), 1.99 – 1.89 (m, 4H, $H_{21,x}$), 1.84 (p, *J* = 6.6 Hz, 2H, H_u), 1.65 – 1.49 (m, 4H, $H_{w,v}$).

^{13}C NMR (101 MHz, acetone-d$_6$) δ: 170.54 (C_c and C_3), 168.55 (C_{cc}), 163.78 (C_z), 159.11 (C_{24} or C_{28}), 158.67(C_{hh}), 158.08 (C_{24} or C_{28}), (157.72, 157.55)($C_{4,16,d,p}$), 152.44 (C_{bb}), (149.84, 149.80, 147.81, 147.77, 145.98, 145.94)($C_{7,9,13,g,l,m,33}$), 144.19 (C_s and C_{18}), (139.78, 139.61)($C_{7,13,g,m}$), 138.16 (C_{26}), (132.85, 132.83, 132.71, 132.65)(C_f,C_n,C_6,C_{14}), (131.86, 131.71, 131.67, 131.28) ($C_{10\text{-}12}$ or $C_{j\text{-}l}$), 129.75 (C_{ff}), 129.07, 128.48, 126.94, 126.90 ($C_{10\text{-}12}$ or $C_{j\text{-}l}$), (124.80, 124.49)(C_s and C_{19}), 120.96, 120.72, 120.65 ($C_{10\text{-}12}$ or $C_{j\text{-}l}$), 114.99 (C_{gg}), 114.63 (C_e,C_o,C_5,C_{15}), 111.69 (C_{aa}), (73.67, 73.35)(C_{23} and C_{29}), (71.42, 71.32)($C_{kk\text{-}ll}$), 70.21 (C_{jj}), 69.45 (C_y), 68.38 (C_{30}), 64.79 (C_{ii}), (62.46, 62.20)(C_q and C_{17}), 57.11 ($C_{n,8}$), 54.87 (C_{32}), 50.56 (C_{20}), 47.90 (C_t), 42.80 (C_{dd}), 39.99 (C_{31}), (32.63, 30.90) ($C_{a,1}$), (26.79, 26.00)($C_{u\text{-}x}$).

HRMS ESI (+): [M+H]$^+$ calculated for $C_{130}H_{128}O_{16}N_{10}Br_2Cl_3$ = 2347.6937, found: 2347.6962, isotopic pattern shown in section 8.2.

3.3 Synthesis of control and references

3.3.1 Synthesis of S19

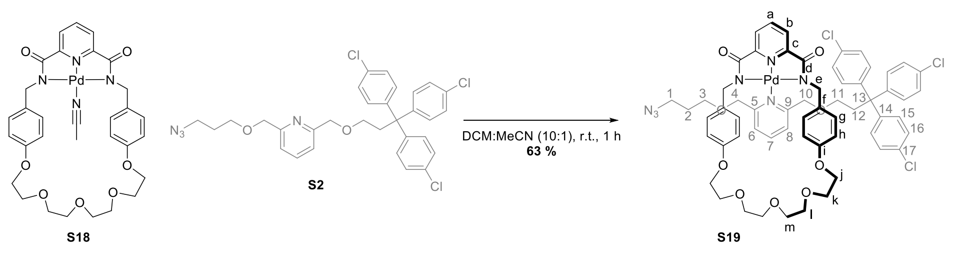

A solution of **S18** (0.022 g, 0.032 mmol) and **S2** (0.025 g, 0.042 mmol, 1.3 eq.) in DCM:MeCN (10:1, 0.900 mL) was stirred at room temperature for 1 h. Solvent was removed and the crude purified by preparative TLC eluting with 30 % MeCN in DCM yielding **S19** complex as a yellow solid (0.025 g, 63 %).

^{1}H NMR (500 MHz, methylene chloride-d$_2$) δ: 8.12 (t, *J* = 7.8 Hz, 1H, H_a), 7.89 (t, *J* = 7.8 Hz, 1H, H_7), 7.80 (d, *J* = 7.8 Hz, 2H, H_b), 7.38 (d, *J* = 7.8 Hz, 1H, H_8), 7.29 (d, *J* = 7.8 Hz, 1H, H_6), 7.25 (d, *J* = 8.7 Hz, 6H, H_{15}), 7.19 (d, *J* = 8.7 Hz, 6H, H_{16}), 6.48 (d, *J* = 8.2 Hz, 4H, H_g), 6.36 (d, *J* = 8.2 Hz, 4H, H_h), 5.00 (s, 2H, H_{10}), 4.88 (d, *J* = 14.0 Hz, 2H, $H_{e'}$), 4.08 – 3.90 (m, 4H, H_j), 3.82 – 3.69 (m, 4H, H_k), 3.69 – 3.67 (m, 2H, H_3), 3.67 – 3.53 (m, 8H, H_m and H_l), 3.48 (t, *J* = 7.2 Hz, 2H, H_{11}), 3.37 (t, *J* = 7.1 Hz, 2H, H_1), 3.33 (s, 2H, H_4), 2.98 (t, *J* = 7.3 Hz, 2H, H_{12}), 2.93 (b, 2H, $H_{e'}$), 1.77 (p, *J* = 6.8 Hz, 2H, H_2).

^{13}C NMR (126 MHz, methylene chloride-d$_2$) δ: 171.41 (C_d), 163.3 (C_5), 158.53 (C_9), 157.95 (C_i), 153.15 (C_c), 145.10 (C_{14}), 141.16 (C_a), 139.41 (C_7), 133.81 (C_f), 132.63 (C_{17}), 130.67 (C_{15}), 128.82 (C_g), 128.70 (C_{16}), 125.01 (C_b), 122.17 (C_8), 121.99 (C_6), 114.38 (C_h), 73.43 (C_4), 71.37 (C_{10}), (71.05, 71.01)($C_{m,l}$), 70.08 (C_j), 69.42 (C_{11}), 68.62 (C_3), 67.89 (C_k), 49.55 (C_1), 49.37 (C_e), 39.71 (C_{12}), 30.08 (C_{13}), 28.55 (C_2).

HRMS ESI (+): $[M+Na]^+$ Calculated for $C_{60}H_{60}Cl_3N_7O_9PdNa$ = 1256.2451, found: 1256.2470

3.3.2 Synthesis of S20

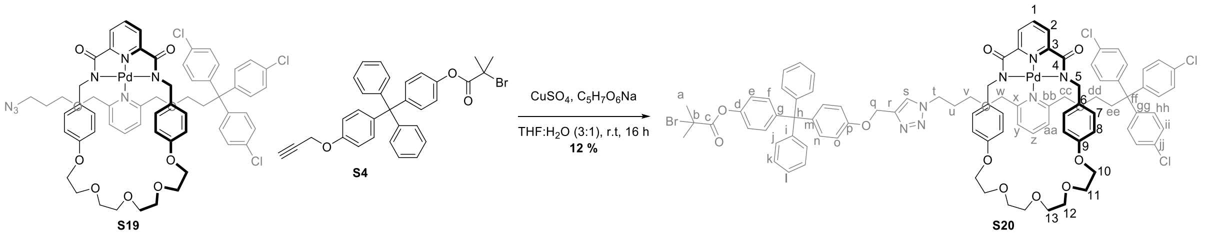

To a solution of **S19** (0.024 g, 0.019 mmol) and **S4** (0.012 g, 0.021 mmol, 1.1 eq.) in THF:H_2O (3:1, degassed for 20 min by bubbling N_2 prior to use, 0.100 mL) was added 50 mM H_2O solutions of $CuSO_4$ (0.040 mL, 0.002 mmol, 0.1 eq.) followed by sodium ascorbate (0.080 mL, 0.004 mmol, 0.2 eq.) and the resulting solution stirred at room temperature overnight. The solution was diluted with DCM, washed with 0.25 M EDTA solution, dried over $MgSO_4$, filtered and concentrated. The crude was purified by preparative TLC, eluting with 20 % acetone in DCM yielding **S20** as a yellow solid (0.004 g, 12 %).

^{1}H NMR (400 MHz, Acetone-d$_6$) δ: 8.27 (t, *J* = 7.8 Hz, 1H, H_1), 8.17 (s, 1H, H_s), 8.08 (t, *J* = 7.8 Hz, 1H, H_z), 7.80 (d, *J* = 7.8 Hz, 2H, H_2), 7.55 (d, *J* = 7.7 Hz, 1H, H_{aa}), 7.33 – 7.18 (m, 23H, $H_{f,j,k,l,y,hh,ii}$), 7.13 – 7.08 (m, 4H, $H_{e,n}$), 6.99 (d, *J* = 8.9 Hz, 2H, H_o), 6.41 (d, *J* = 8.2 Hz, 4H, H_7), 6.27 (d, *J* = 8.1 Hz, 4H, H_8), 5.19 (s, 2H, H_q), 4.98 (s, 2H, H_w or H_{cc}), 4.83 (d, *J* = 13.7 Hz, 2H, $H_{5'}$), 4.43 (t, *J* = 6.8 Hz, 3H, H_v), 4.01-3.92 (m, 4H, H_{cc} or H_w), 3.84– 3.62 (m, 12H, H_{10-12}), 3.60 – 3.50 (m, 6H, H_{dd}, H_{13}), 3.16 (d, *J* = 13.8 Hz, 2H, $H_{5'}$), 3.09 (m, 4H, $H_{ee,t}$), 2.18 (p, *J* = 7.2 Hz, 2H, H_u), 2.06 (s, 6H, H_a).

^{13}C NMR (101 MHz, Acetone-d$_6$) δ: 171.54 (C_4), 170.56 (C_c), 163.66 (C_x), 158.78 (C_9), 158.58 (C_{bb}), 157.69 (C_p), 153.53 (C_3), 149.82 (C_d), 147.76 (C_m), 146.03 (C_{gg}), 145.93 (C_i), 144.17 (C_r), 142.33 (C_a), 140.22 (C_z), 139.78 (C_g), 134.56 (C_6), 132.84 (C_{jj}), 132.67 (C_{ii}), 131.69 (C_{hh}), 131.46, 129.39 (C_l), 129.08 (C_7), 128.49 (C_k), 126.92 (C_j), 125.54 (C_2), 124.86 (C_s), 123.41 (C_{aa}), 123.07 (C_y), 120.97 (C_e), 114.95 (C_8), 114.57 (C_o), 74.31 (C_{cc}), 72.33-71.44 ($C_{dd,12,13}$), 70.50 ($C_{10,11}$), 69.75 (C_t), 69.31, 68.36 (C_w), 64.78 (C_h), 62.47 (C_q), 57.13 (C_b), 55.21 (C_{ff}), 49.68 (C_5), 48.29 (C_v), 39.84 (C_{ee}), 30.78 (C_a).

HRMS ESI (+): $[M+Na]^+$ Calculated for $C_{92}H_{87}O_{12}N_7BrCl_3NaPd$ = 1794.3589, found: 1794.3616, isotopic pattern shown in section 8.3.

3.3.3 Synthesis of S21

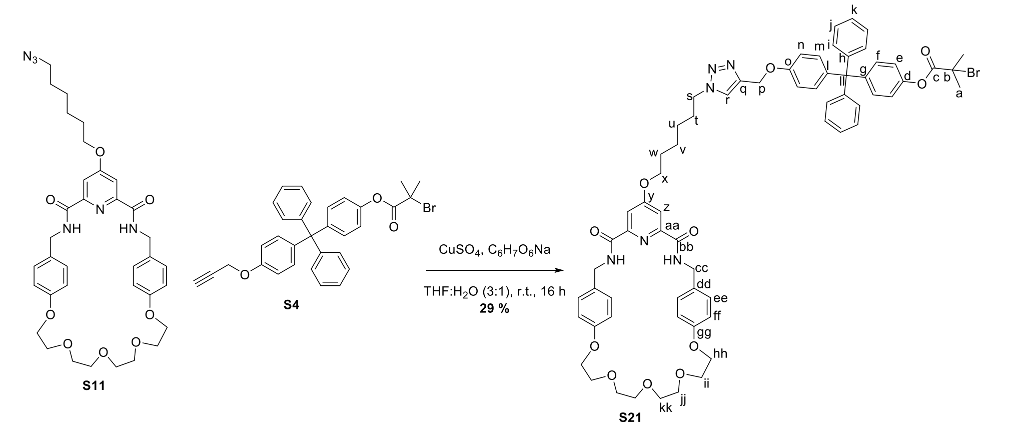

To a solution of **S11** (0.044 g, 0.065 mmol) and **S4** (0.058 g, 0.108 mmol, 1.7 eq.) in THF:H_2O (3:1, degassed for 20 min by bubbling N_2 prior to use, 0.350 mL) was added $CuSO_4$ (0.002 g, 0.013 mmol, 0.2 eq.) followed by sodium ascorbate (0.5 g, 0.026 mmol, 0.4 eq.) and the resulting solution stirred at room temperature overnight. The solution was diluted with DCM, washed with 0.25 M EDTA solution, dried over $MgSO_4$, filtered and concentrated. The crude was purified by SiO_2 column chromatography, eluting with 20 % MeCN in DCM, yielding **S21** as a white solid (0.022 g, 29 %).

^{1}H NMR (400 MHz, Acetone-d$_3$) δ: 8.97 (t, *J* = 6.3 Hz, 2H, NH), 8.08 (s, 1H, H_r), 7.80 (s, 2H, H_z), 7.35 – 7.19 (m, 10H, $H_{i\text{-}k}$), 7.16 (d, *J* = 8.6 Hz, 4H, H_{ee}), 7.14 – 7.09 (m, 4H, $H_{m,f}$), 6.98 (d, *J* = 8.9 Hz, 2H, H_n or H_e), 6.84 (d, *J* = 8.6 Hz, 4H, H_{ff}), 5.16 (s, 2H, H_p), 4.50 (d, *J* = 6.3 Hz, 4H, H_{cc}), 4.46 (t, *J* = 7.1 Hz, 2H, H_x), 4.26 (t, *J* = 6.4 Hz, 2H, H_s), 4.07 (t, *J* = 4.6 Hz, 4H, H_{hh}), 3.78 (t, *J* = 4.6 Hz, 4H, H_{ii}), 3.61 (m, 8H, $H_{jj,kk}$), 2.06 (s, 6H, H_a), 1.98 (p, *J* = 7.2 Hz, 2H, H_w), 1.87 (p, *J* = 6.4 Hz, 2H, H_t), 1.58 (p, *J* = 6.4 Hz, 2H, H_v), 1.44 (p, *J* = 6.4 Hz, 2H, H_u).

^{13}C NMR (101 MHz, Acetone-d$_3$) δ: 170.55 (C_c), 168.74 (C_y), 163.76 (C_{bb}), 159.08 (C_{gg}), 157.70 (C_o), 152.24 (C_{aa}), 149.83 (C_d), 147.76 (C_g or C_l), 145.94 (C_g or C_l), 144.18 (C_q), 139.77 (C_{ll}), 132.84 (C_i or C_j, or C_k), 132.12 (C_{dd}), 131.69 (C_i or C_j or C_k or C_f), 129.55 (C_{ee}), 128.47 C_m), 126.94 (C_i or C_j or C_k), 124.52 (C_r), 120.95 (C_e), 115.30 (C_{ff}), 114.63 (C_n), 111.42 (C_z), 71.39 (C_{kk}), 71.37 (C_{kk}), 70.20 (C_{ii}), 69.49 (C_x), 68.49 (C_{hh}), 64.78 (C_h), 62.43 (C_p), 57.11 (C_b), 50.53 (C_s), 42.61 (C_{cc}), 30.86 (C_w), 30.77 (C_a), 29.23 (C_t), 26.73 (C_u), 25.93 (C_v).

HRMS ESI (+): $[M+H]^+$ calculated for $C_{67}H_{71}BrN_6O_{11}Na$ = 1237.4262, found: 1237.4233

3.3.4 Synthesis of S22

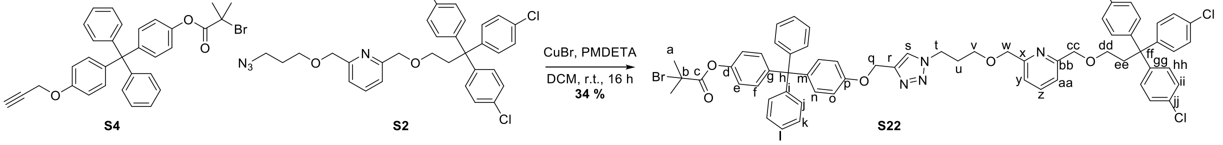

A solution of **S4** (0.007 g, 0.013 mmol), **S2** (0.008 g, 0.013 mmol, 1 eq.) and PMDETA (0.003 mL, 0.002 g, 0.013 mmol, 1 eq.) was degassed by 3 free-pump-thaw cycles then transferred via cannula onto CuBr (0.001 g, 0.007 mmol, 0.5 eq.) and the resulting solution stirred at room temperature overnight. The mixture was diluted with DCM, washed with 0.25 M EDTA and brine, dried over $MgSO_4$, filtered and concentrated. The crude was purified by preparative TLC, eluting with 50 % EtOAc in petroleum ether to yield **S22** as a white solid (0.005 g, 34 %).

^{1}H NMR (500 MHz, MeCN-d$_3$) δ: 7.82 (s, 1H, H_s), 7.73 (t, *J* = 7.8 Hz, 1H, H_z), 7.34 – 7.17 (m, 24H, $H_{f,j,kl,y,aa,hh,ii}$), 7.14 (d, *J* = 8.9 Hz, 2H, H_n), 7.03 (d, *J* = 8.8 Hz, 2H, H_e), 6.89 (d, *J* = 8.9 Hz, 2H, H_o), 5.09 (s, 2H, H_q), 4.46 (m, 4H, H_w and H_v), 4.36 (s, 2H, H_{cc}), 3.47 (t, *J* = 5.9 Hz, 2H, H_t), 3.24 (t, *J* = 7.2 Hz, 2H, H_{dd}), 2.90 (t, *J* = 7.2 Hz, 2H, H_{ee}), 2.25 (p, *J* = 7.5 Hz, 2H, H_u), 1.88 (s, 6H, H_a).

^{13}C NMR (126 MHz, MeCN-d$_3$) δ: 171.08 (C_c), 158.37 ($C_{x,bb}$), 157.43 (C_p), 149.67 (C_d), 147.95 (C_i), 146.32 (C_g), 146.15 (C_{gg}), 144.19 (C_r), 140.22 (C_m), 138.76 (C_z), 132.88 (C_{jj}), 131.60 (C_l), (C_{hh}), 131.47 (C_k), 129.05 (C_{ii}), 128.73 (C_j), 114.84 (C_o), (74.10, 74.00 ($C_{w,cc}$)),68.72 (C_{dd}), 68.00 (C_t), 66.09 (C_d), 64.92 (C_h), 62.39 (C_q), 55.28 (C_{ff}), 48.07 (C_v), 40.20 (C_{ee}), 32.64, 30.98 (C_u), 30.07 (C_u), 29.83 (C_a).

HRMS ESI (+): $[M+Na]^+$ Calculated for $C_{63}H_{56}O_5N_4BrCl_3Na^+$ = 1155.2397, found: 1155.2438

4 Synthesis of polymers

4.1 Representative Procedure for SET-LRP of Methyl Acrylate Using Mechanophore Initiators

Methyl acrylate was filtered through basic alumina to remove the inhibitor prior to use. A stock catalytic solution of Me_6TREN (16 µL, 0.060 mmol) and $CuBr_2$ (5.6 mg, 0.025 mmol) in dry DMSO (1 mL) was prepared. To a 5 mL microwave vial was added the appropriate initiator compound along with catalytic solution, methyl acrylate and dry DMSO. This solution was degassed by bubbling with N_2 for 10 min. A Cu(0) wire wrapped around a stirrer bar, having been cleaned in 12 N HCl for 10 min, was added to the reaction mixture. The reaction mixture was degassed for a further 2 min before being allowed to stir for 15 - 40 min (until the extent of polymerization, as determined approximately by the increasing viscosity of the solution, was deemed acceptable). The solution was added dropwise to a solution of vigorously stirred methanol; the precipitated polymer was recovered and dried under vacuum for two days to yield a white material. Molecular weight and polydispersity indices were determined using an analytical SEC that had been calibrated with PMMA standards.

4.2 Synthesis of mechanophore and control polymers

4.2.1 Synthesis of 1_{Pd}

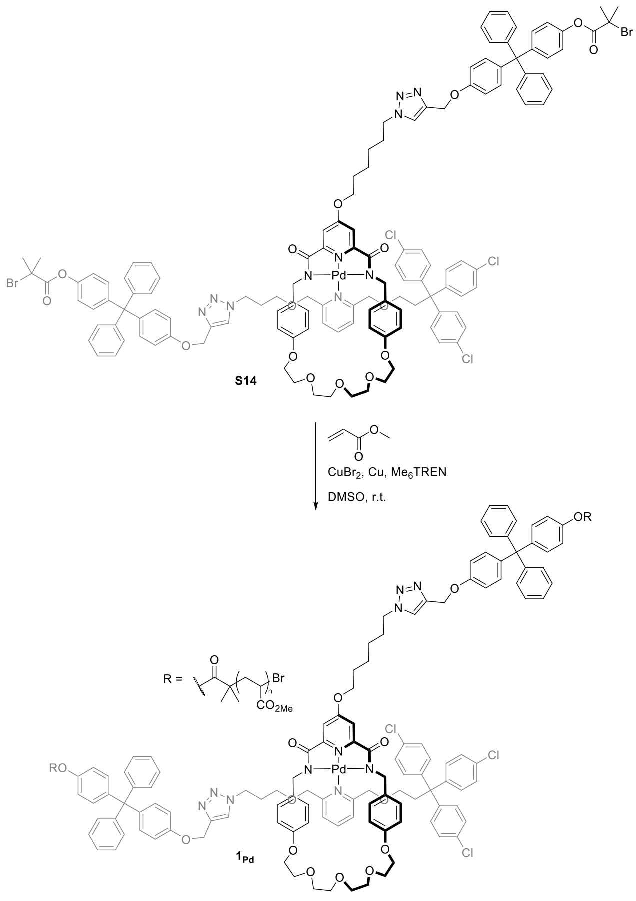

Synthesis followed the representative procedure. **S14** (9 mg, 4.0 µmol, 1.0 eq.), 15.0 µL of catalytic solution ($CuBr_2$: 0.4 µmol, 0.1 eq.; Me_6TREN: 1.2 µmol, 0.3 eq.), methyl acrylate (400 µL, 4.0 mmol, 1000.0 eq.), Cu (0) wire (~3 cm, ~30 mg, 0.5 mmol, ~125.0 eq.) and dry DMSO (400 µL) were used in the reaction to yield **1_{Pd}** (0.228 mg, M_n = 146 kDa; $Đ$ = 1.20).

4.2.2 Synthesis of 1_H

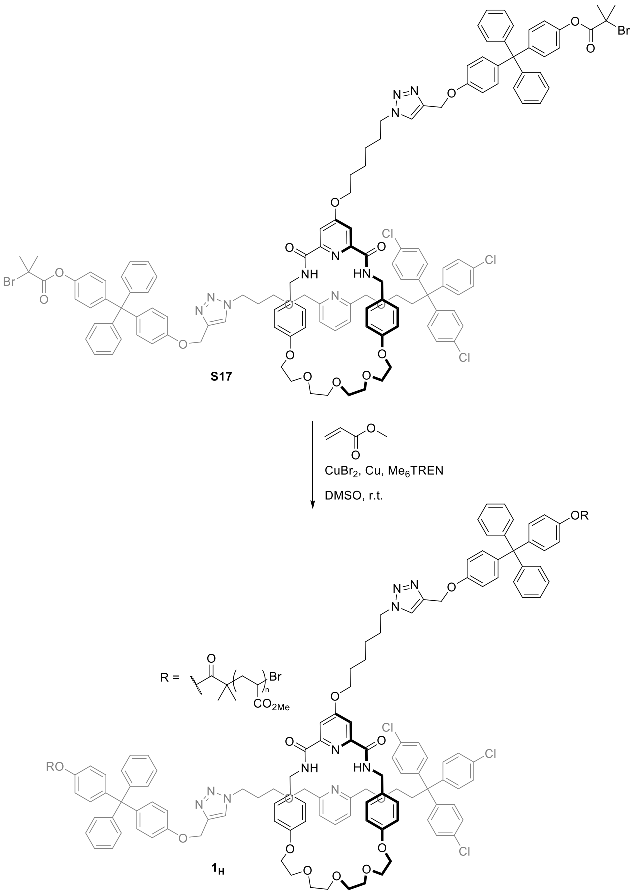

Synthesis followed the representative procedure. **S17** (8 mg, 3.4 µmol, 1.0 eq.), 14.0 µL of catalytic solution ($CuBr_2$: 0.35 µmol, 0.1 eq.; Me_6TREN: 1.0 µmol, 0.3 eq.), methyl acrylate (300 µL, 3.4 mmol, 1000.0 eq.), Cu (0) wire (~3 cm, ~30 mg, 0.5 mmol, ~150.0 eq.) and dry DMSO (300 µL) were used in the reaction to yield $\mathbf{1_H}$ (198 mg, M_n = 129 kDa; $Đ$ = 1.17).

4.2.3 Synthesis of S23

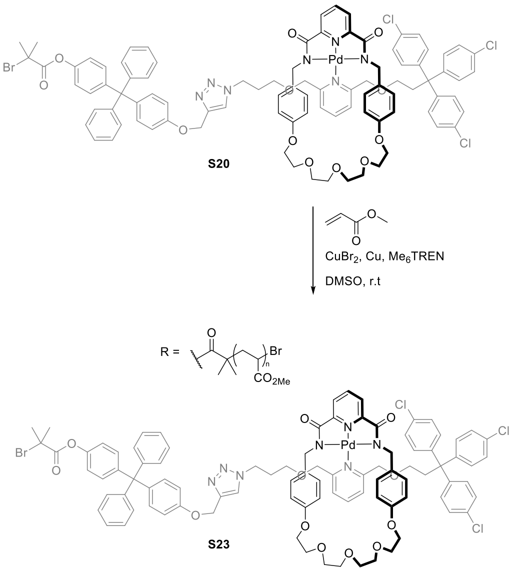

Synthesis followed the representative procedure. **S20** (4 mg, 2.0 µmol, 1.0 eq.), 5.0 µL of catalytic solution ($CuBr_2$: 0.2 µmol, 0.05 eq.; Me_6TREN: 0.3 µmol, 0.15 eq.), methyl acrylate (200 µL, 2.6 mmol, 1000.0 eq.), Cu (0) wire (~3 cm, ~30 mg, 0.5 mmol, ~250.0 eq.) and dry DMSO (200 µL) were used in the reaction to yield **S23** (139 mg, M_n = 140 kDa; $Đ$ = 1.20).

4.2.4 Synthesis of 2_H

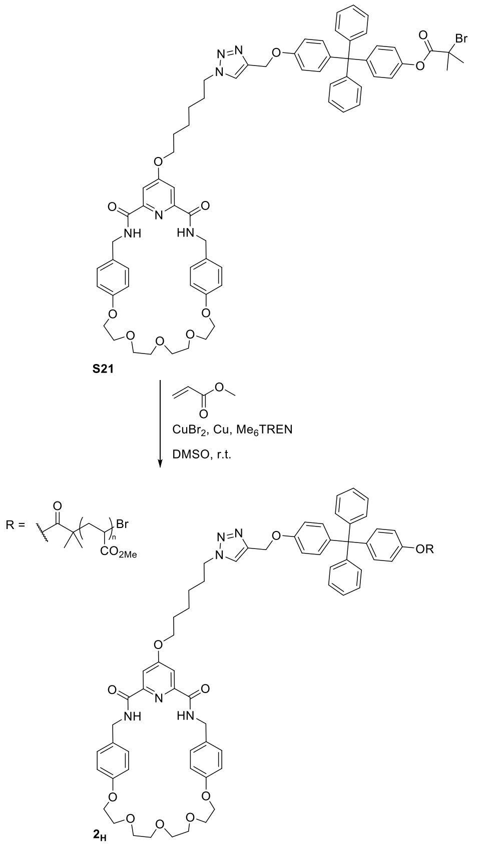

Synthesis followed the representative procedure. **S21** (0.003 mg, 2.5 µmol, 1.0 eq.), 5.0 µL of catalytic solution ($CuBr_2$: 0.13 µmol, 0.05 eq.; Me_6TREN: 0.4 µmol, 0.15 eq.), methyl acrylate (220 µL, 2.5 mmol, 1000.0 eq.), Cu (0) wire (~3 cm, ~30 mg, 0.5 mmol, ~200.0 eq.) and dry DMSO (2200 µL) were used in the reaction to yield **2_H** (143 mg, M_n = 104 kDa; $Đ$ = 1.07).

4.2.5 Synthesis of 6

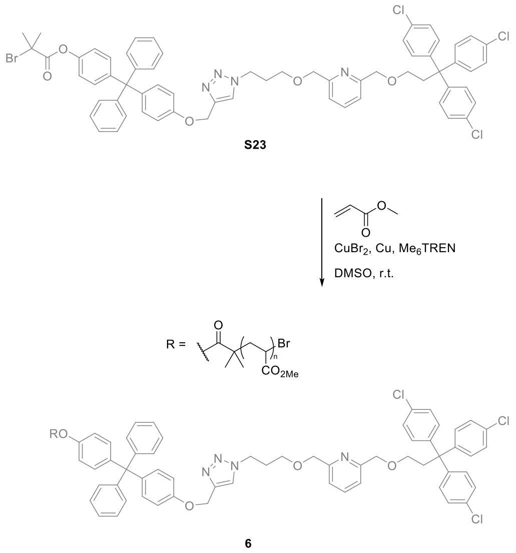

Synthesis followed the representative procedure. **S23** (8 mg, 7.0 µmol, 1.0 eq.), 14.0 µL of catalytic solution ($CuBr_2$: 0.35 µmol, 0.05 eq.; Me6TREN: 1.1 µmol, 0.15 eq.), methyl acrylate (630 µL, 7.0 mmol, 1000.0 eq.), Cu (0) wire (~3 cm, ~30 mg, 0.5 mmol, ~71.0 eq.) and dry DMSO (630 µL) were used in the reaction to yield **6** (140 mg, M_n = 78.6 kDa; *Đ* = 1.22).

4.3 SEC Data for Synthesised Polymers

Table S1. SEC data for synthesised polymers

Polymer	M_n/ kDa	*Đ*
$\mathbf{1_{Pd}}$	146	1.20
$\mathbf{1_{H}}$	129	1.17
**S23**	140	1.19
$\mathbf{2_{H}}$	104	1.07
**6**	78.6	1.22

4.4 SEC traces for Synthesised Polymers

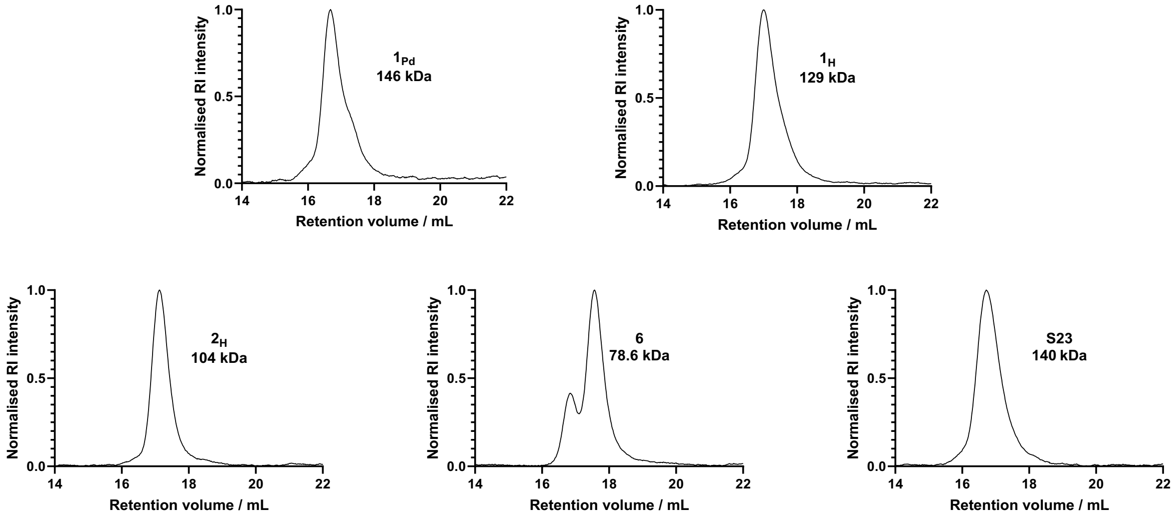

Figure S1. SEC traces for polymer **1_{Pd}**, **1_H**, **2_h**, **6** and **S24**

5 Mechanochemical activation via ultrasound

5.1 General procedure for sonication experiments

The appropriate polymer (20 mg) was added to a Suslick cell and dissolved in MeCN (20 mL). The solution was degassed by bubbling N_2 through it for a minimum of 10 min prior to the start of sonication; bubbling of N_2 was also maintained throughout the experiment. The Suslick cell was cooled with an ice bath throughout the duration of the sonication to maintain a temperature of ~ 5-10 °C inside the cell. Pulsed ultrasound was applied to the system (1 s ON / 1 s OFF, 25% amplitude (10.4 W.cm^{-2}), 20 kHz) for the desired period of time. After sonication, the solvent was evaporated, and the polymer was analysed by SEC and NMR spectroscopy. The post-sonication polymer was recovered and washed with MeOH to extract any small molecules not attached to polymer chains. The remaining MeOH-washed polymer and the concentrated MeOH washings were then analysed by NMR spectroscopy.

5.2 Representative sonication of 1_{Pd}

Sonication of $\mathbf{1_{Pd}}$, using the methodology described in the general procedure, was carried out three times to determine the extent and pathway of rotaxane dissociation (**Table S2**). SEC analysis of the sonicated polymers showed complete cleavage of the PMA chains (M_n of the post-sonication material was less than half of that of the pre-sonication polymer).

Comparison of the ^{1}H NMR spectra of pre- and post-sonication polymer showed that unstoppering occurred upon mechanochemical activation (**Figure S2**). It is evidenced by the shift in macrocycle aromatic protons (*a*) from 6.3 ppm in the starting polymer to 6.8 ppm indicating the macrocycle has separated from the axle in a conversion of 81 % (77 % on average over 3 runs). The absence of the ethylene linker to the stopper in the axle (protons *b* and *c*) compared to the post sonication polymer shows that the rotaxane dissociated by unstoppering. The presence of protons *d* and *e* from the axle shows that the axle is intact between the polymer and the pyridine unit. Comparing the post-sonication polymer to the axle reference shows the absence of the axle pyridine proton *f* and the two methylene protons *g* and *h* suggesting that the whole pyridine unit is eliminated after mechanochemical bond scission. The formation of 4,4'-dichlorobenzophenone (*i*) and 4,4',4''-trichlorotrityl alcohol (*j*), after sonication and in the MeOH wash phase respectively, suggests that unstoppering occurs via scission of the bond between the stopper and atom *c* (see scheme S3 for proposed formation of 4,4'-dichlorobenzophenone).

Conversion of the rotaxane dissociation was calculated by the relative integrations of the rotaxane and macrocycle aromatic protons (H_a) in the post-sonication methanol washed polymer. The amount of demetalation was calculated by the relative integrations of macrocycle aromatic protons H_a and $H_{a'}$ in the post-sonication methanol washed polymer. The amount of 4,4'-dichlorobenzophenone generated was calculated by the relative integrations of the 4,4'-dichlorobenzophenone protons compared to the sum of the two triazole-adjacent methylene protons (H_k and H_d) in the macrocycle and the axle in the post-sonication polymer before methanol wash.

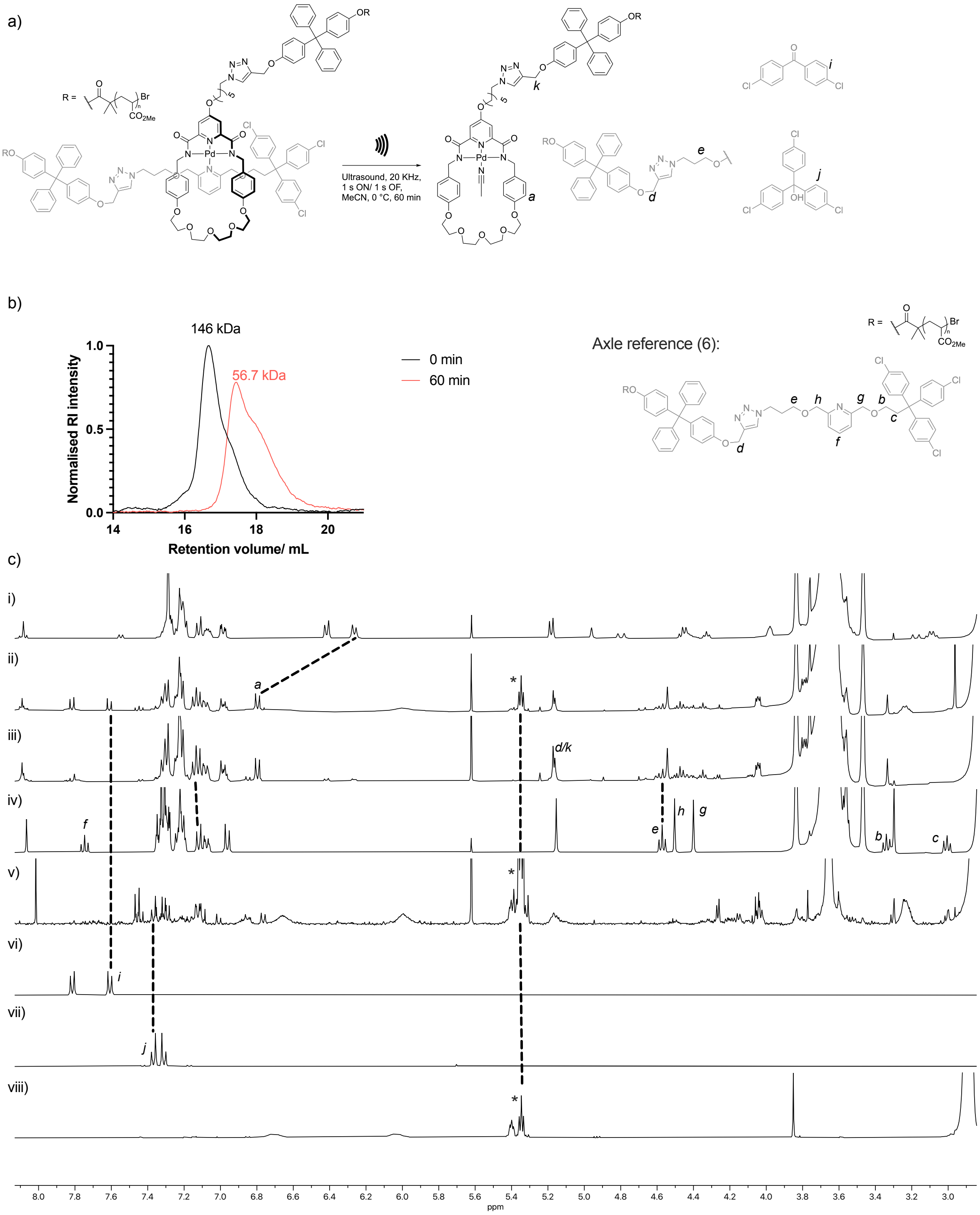

Figure S2. a) Sonication of $\mathbf{1}_{\mathbf{Pd}}$ after 60 min. b) GPC analysis before and after sonication. c) ^{1}H NMR (400 MHz, acetone-d$_6$) comparison of pre- (i), post-sonication (ii) and MeOH washed (iii) polymer compared to axle reference **6** (iv). And MeOH phase (v) compared to references of 4,4'-dichlorobenzophenone (vi) and 4,4',4''-Trichlorotrityl alcohol (vii). Concentrated solution after soaking syringe in DCM/Acetone (viii) shows that peak at 5.3 ppm (*) is due to external impurity from syringe (see spectrum S67).

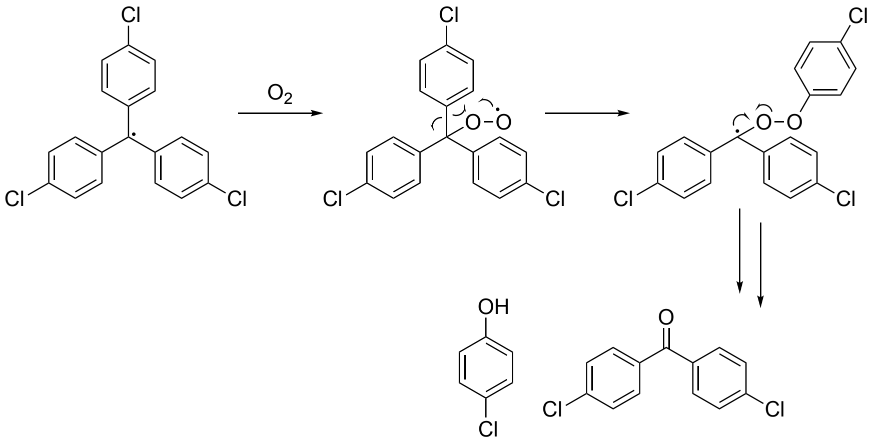

Scheme S3. Proposed mechanism for the formation of *4,4'-dichlorobenzophenone* based on reference 27 in the manuscript.

5.3 Representative sonication of 1_H

Sonication of $\mathbf{1_H}$, using the methodology described in the general procedure, was carried out three times to determine the extent and pathway of rotaxane dissociation (**Table S2**). SEC analysis of the sonicated polymers showed complete cleavage of the PMA chains (M_n of the post-sonication material was less than half of that of the pre-sonication polymer).

Comparison of the ^{1}H NMR spectra of pre- and post-sonication polymer showed that dethreading occurred upon mechanochemical activation (**Figure S3**). It is evidenced by the shift in macrocycle aromatic protons (*a*) from 6.4 ppm in the starting polymer to 6.8 ppm indicating the macrocycle has separated from the axle in a conversion of 81 % (84 % on average over 3 runs). The presence of all signals of both the macrocycle and axle after sonication shows that the rotaxane dissociated by dethreading. NMR integration suggests this is the only observable species, although a small amount (<5 %) of the 4,4'-dichlorobenzophenone is present suggesting some unstoppering is present but below the limit of which can be observed by ^{1}H NMR analysis of the polymer.

a)

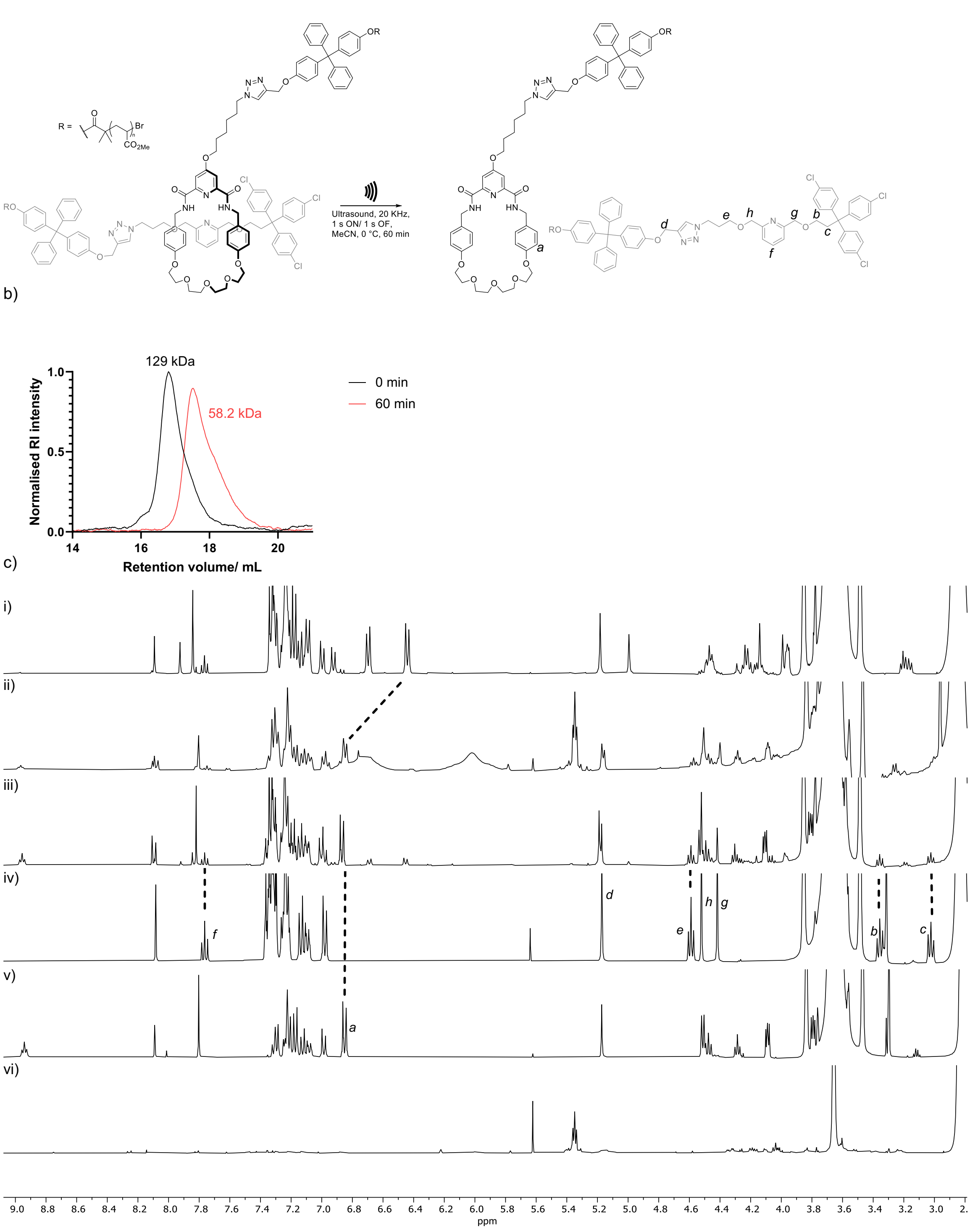

Figure S3. a) Sonication of $\mathbf{1_H}$ after 60 min. b) GPC analysis before and after sonication. c) ^{1}H NMR (400 MHz, acetone-d$_6$) comparison of pre- (i), post-sonication (ii) and MeOH washed (iii) polymer compared to references of axle **6** (iv) and macrocycle $\mathbf{2_H}$ (v), and MeOH phase (vi). Peak at 5.3 ppm is due to external impurity from syringe (see Figure S2).

5.4 Representative sonication of S23

Sonication of **S23**, using the methodology described in the general procedure, was carried out three times to determine the extent and pathway of rotaxane dissociation (**Table S2**). SEC analysis of the sonicated polymers showed complete cleavage of the PMA chains (M_n of the post-sonication material was less than half of that of the pre-sonication polymer).

Comparison of the spectra before and after sonication show that the control polymer is largely untouched by sonication. Although there is a small amount of demetalation (19 %, evidenced by de-metalated macrocycle proton *b′* corresponding to the adjacent aromatic signal) which is also observed in the other Pd-rotaxane, there is no evidence of rotaxane dissociation to the separate component parts, shown by the close match of signal *b′* to the interlocked but demetalated rotaxane $\mathbf{1_H}$, or any stopper fragments in the methanol wash phase.

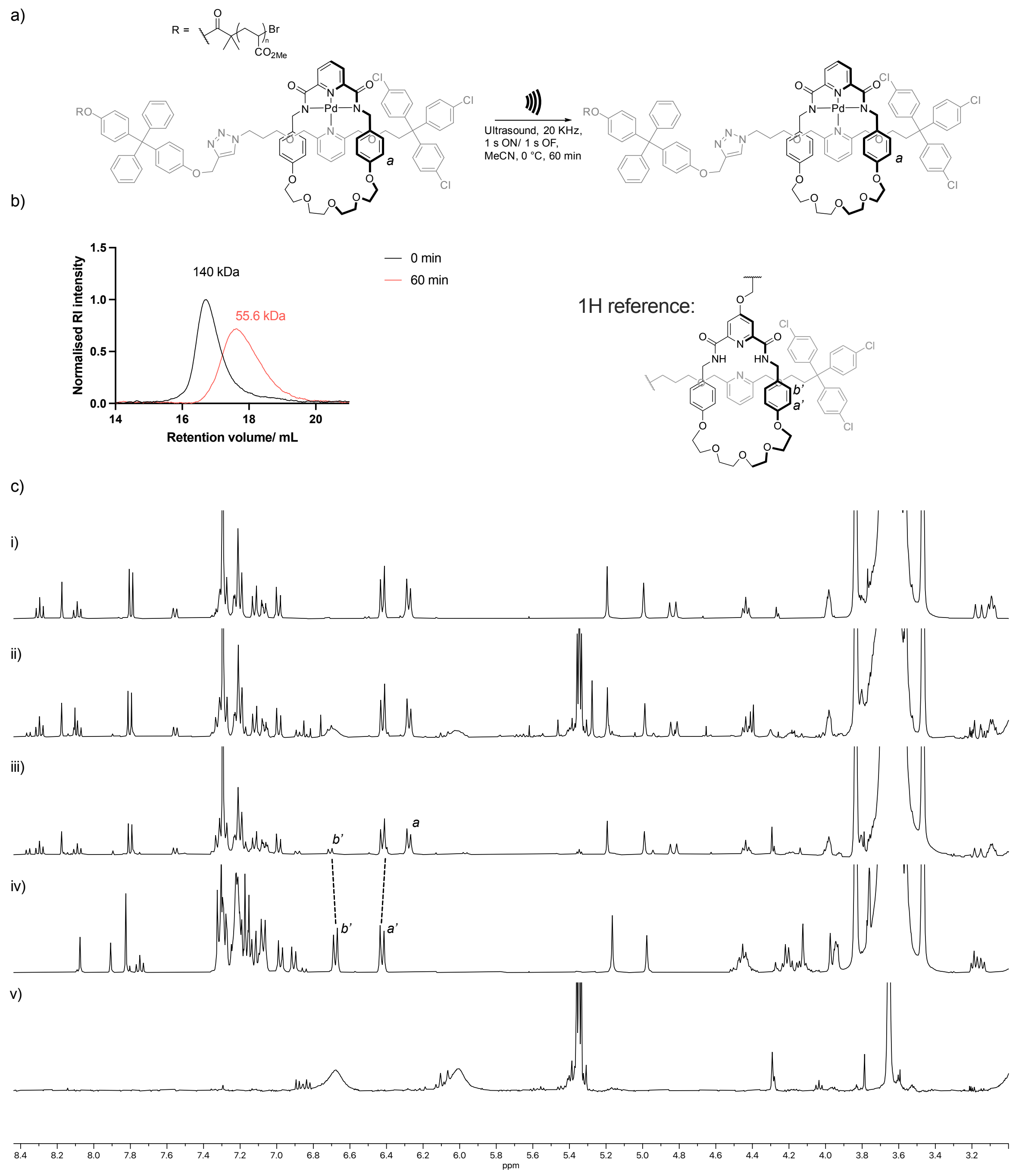

Figure S4. a) Sonication of **S23** after 60 min. b) GPC analysis before and after sonication. c) ^{1}H NMR (400 MHz, acetone-d_6) comparison of pre- (i), post-sonication (ii), MeOH-washed polymer (iii), reference of 1H (iv) and MeOH phase (v). Peak at 5.3 ppm is due to external impurity from syringe (see Figure S2).

5.5 Summary of mechanophores activated by sonication

Table S2. Analysis of mechanical activation of rotaxanes

Mechanophore		Pre-sonication		Pre-sonication		Conversion			
		M_n (kDa)	Đ	M_n (kDa)	Đ	Intact mechanophore	Unstoppering	Dethreading	Benzophenone
**1_{Pd}**	Run 1	146	1.20	69.3	1.29	19 %	81 %	0 %	13 %
	Run 2	146	1.20	71.3	1.29	21 %	79 %	0 %	21 %
	Run 3	146	1.20	57.3	1.24	30 %	70 %	0 %	14 %
**1_H**	Run 1	129	1.17	58.2	1.87	13 %	0 %	80 %[c]	5 %
	Run 2	129	1.17	60.1	1.24	15 %	0 %	78 %[c]	4 %
	Run 3	129	1.17	58.4	1.20	19 %	0 %	76 %[c]	3 %
**S23**	Run 1	140	1.19	55.6	1.29	100 %	0 %	0 %	12 % [a]
	Run 2	140	1.19	72.7	1.32	100 %	0 %	0 %	3 % [a]
	Run 3	140	1.19	72.6	1.36	81%[b]	0 %	0 %	0 %

[a] (caused by external impurity), [b] (19 % background de-metalation), [c] corrected values subtracting 6 % **2_H** present as an impurity in **1_H**

6 Computational modelling

6.1 General method

CoGEF calculations were performed using Gaussian16 following Beyer's method[7]. The structure of the mechanophore was built in GaussianView6. The distance between the terminal methyl groups was constrained and increased by increments with 0.2 Å. Each step was run with DFT (B3LYP/6-31G) in vacuum. The relative energy of each intermediate was determined by setting the energy of the initial state at 0 kJ/mol. F_{max} values were determined from the slope of the final 10 points before bond scission.

We performed simulations using the External Force is Explicitly Included (EFEI) method to investigate the effect of mechanical forces on the rotaxane's molecular structures. The EFEI calculations were conducted using the semiempirical GFN2-xTB method, using ORCA 6.0.1 interfaced with xtb. ORCA allows for the direct inclusion of external force vectors during geometry optimizations, enabling the study of force-induced structural changes and mechanochemical behaviour. In our case, force was applied along a defined coordinate where atoms indicated in Fig. S7 were selectively subjected to directional force vectors.

To simulate the dynamic effects of external mechanical forces, we performed molecular dynamics (MD) simulations at the GFN2-xTB level of theory using the ORCA/xtb interface. Atomic positions were recorded every step of the trajectory. MD protocol was structured in two stages: an initial equilibration phase and a subsequent force-application phase. In both phases, temperature was controlled using a CSVR8 thermostat set at 300 with a time constant of 20.0 fs.

Equilibration was started from an optimised geometry by assigning random atomic velocities were initialized at 300 K. The system was equilibrated for 2000 steps with a timestep of 0.5 fs. The desired temperature was reached after ~1000 steps (see Fig. S8-13).

In the force application phase, a mechanical force, acting on the selected atoms defined above (see Fig. S8-23), was introduced. Force was applied through a spring constant that mimics a constant force pulling experiment acting on a collective variable defined as the distance between the selected atoms. The MD simulation then proceeded for an additional 10 000 steps at the given force.

In the MD simulations, external mechanical force was applied to the rotaxanes using harmonic restraints to selected atomic coordinates. The potential energy values reported in the output files include contributions from both intrinsic molecular interactions and the externally applied harmonic bias. To analyse the internal energy of the system independently of the external work, a correction was applied to remove the artificial potential energy associated with the applied force. The corrected energy was computed as:

$$E(t) = \left[E_{pot}(t) - \frac{1}{2}k\big(x_r - x(t)\big)^2\right]$$

Where: $E_{pot}(t)$ is the total potential energy at time (t) including both internal and restraint energy terms; k is the harmonic force constant used for the restraint; x_r is the target value of the restraint; and $x(t)$ is the value of the collective variable.

This correction removes the contribution of the harmonic restraint energy (the external mechanical work) from the potential energy. To facilitate comparison across simulations and improve visualisation, we set the energy baseline to the average of the second half of the equilibration phase,

when the desired temperature was reached (see above). This standardisation allows the relative energy changes to be plotted (ΔE) over time.

6.2 CoGEF of 1_{Pd}

The structure was initially stretched to the point where the macrocycle reaches the stopper using a semi empirical method before DFT was used to simulate the macrocycle pulling against the stopper. Pulling atoms are indicated with pink discs.

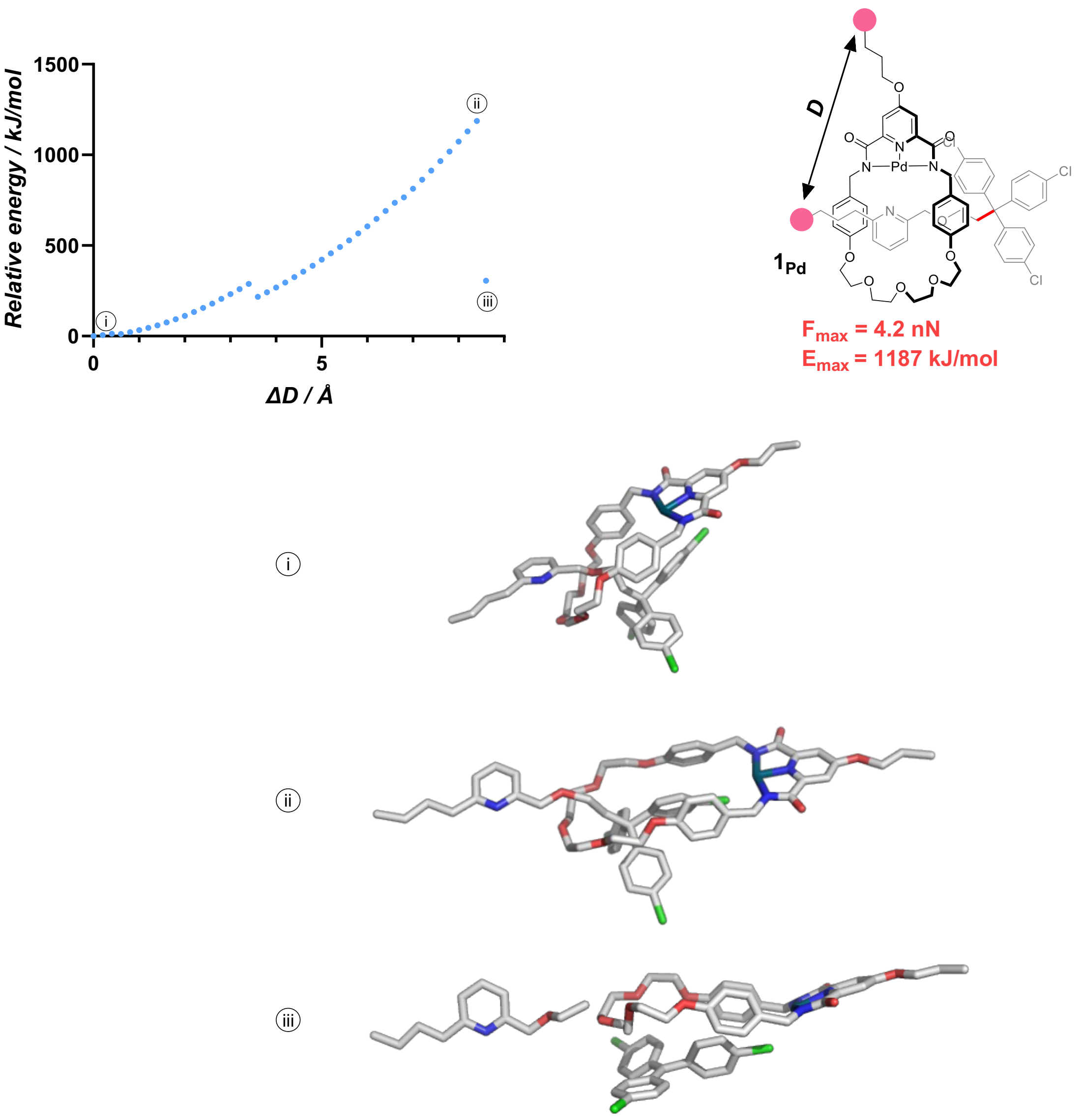

Figure S5. Evolution of energy of $\mathbf{1_{Pd}}$ simulated elongation (CoGEF, DFT, B3LYP/6-31G) and structures at E_0 (i), E_{max} (ii) and scission (iii). F_{max} was determined from the final 10 points of the energy/elongation curve. Scissile bond shown in red

6.3 CoGEF of 1_H

The structure was initially stretched to the point where the macrocycle reaches the stopper using a semi empirical method before DFT was used to simulate the macrocycle pulling against the stopper. Pulling atoms are indicated with pink discs.

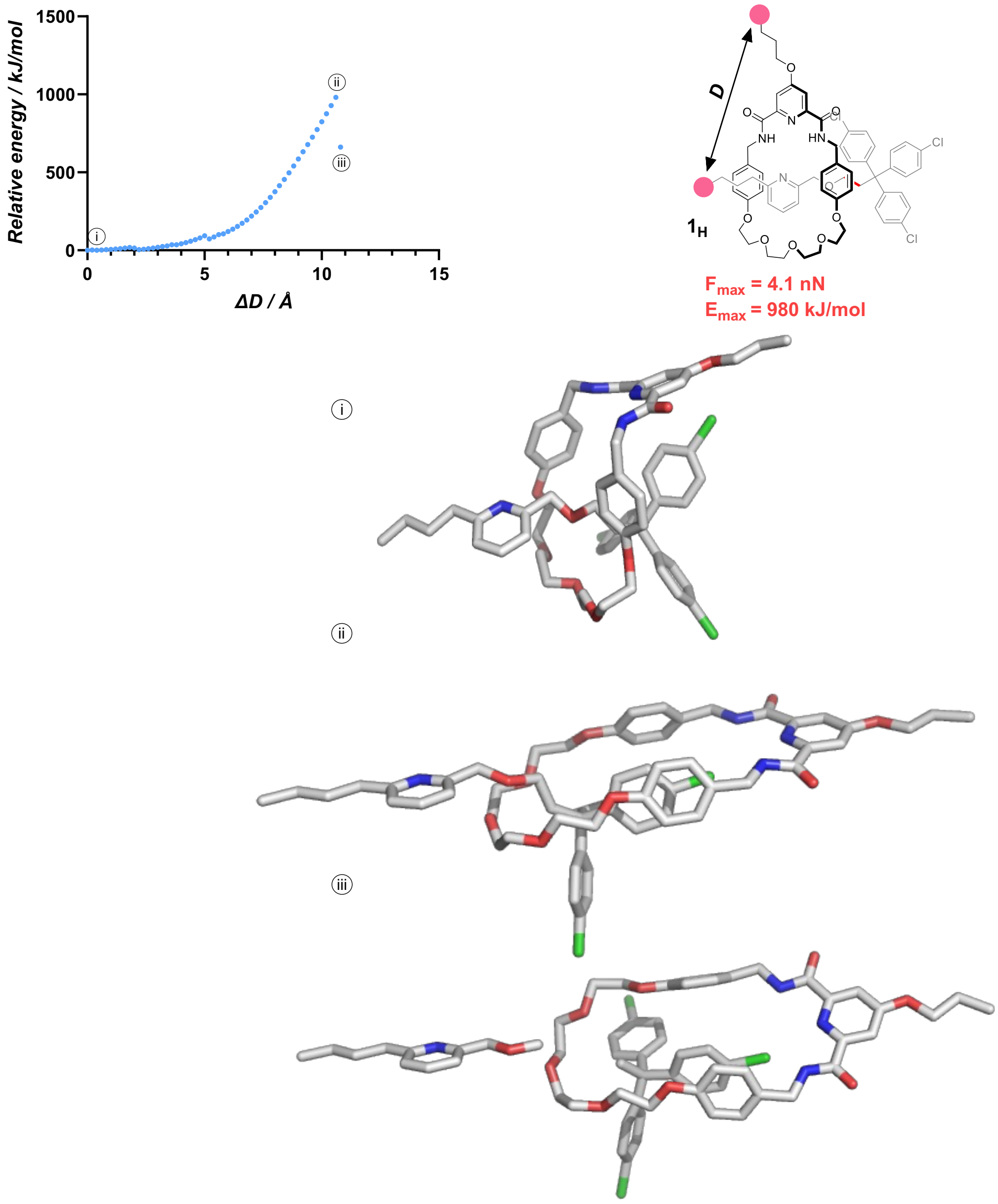

Figure S6. Evolution of energy of $\mathbf{1_H}$ simulated elongation (CoGEF, DFT, B3LYP/6-31G) and structures at E_0 (i), E_{max} (ii) and scission (iii). F_{max} was determined from the final 10 points of the energy/elongation curve. Scissile bond shown in red

6.4 EFEI of 1_{Pd} and 1_H

We performed geometry optimisations using the External Force is Explicitly Included method (EFEI),[8] where external force (from 0 to 5.5 nN, in 250 pN increments) was applied to the anchor atoms indicated by pink disks in Figure S7. All geometry optimizations and calculations were performed at the GFN2-xTB level of theory. This EFEI method enabled us to detect the largest difference in energy (ΔE) indication scission of the Pd-pyridine bond of the axle on Pd-rotaxane between 1.25-1.50 nN, no other bond scission was observed for either rotaxanes.

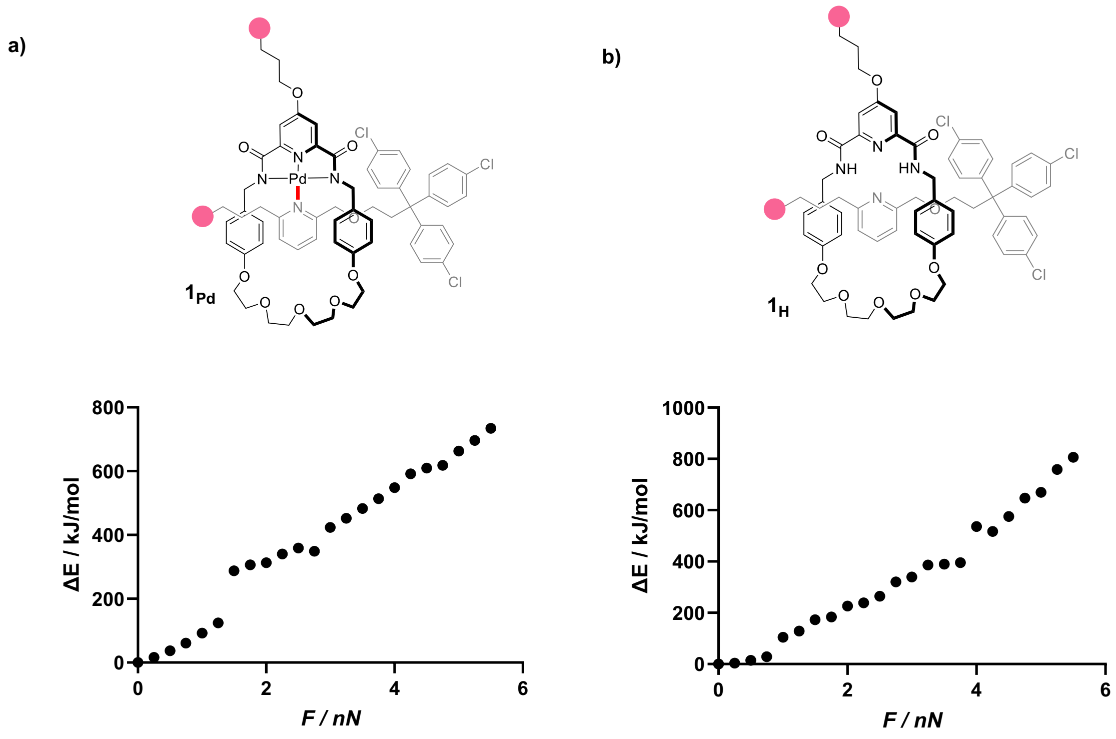

Figure S7. Model structure and EFEI landscape upon external force (GFN2-xTB) of $\mathbf{1_{Pd}}$ (a) and $\mathbf{1_H}$ (b). Scissile bond indicated in red

6.5 Molecular Dynamics Simulations

Molecular dynamics simulations (20 trajectories) starting from randomized velocities at 3.00 nN or 3.50 nN for model rotaxanes, were performed as dethreading is predicted to be a low force pathway. From these calculations we extracted the evolution of the distance between one of the Cl atoms of the stopper and the central O atom of the glycol chain of the macrocycle relative to the centre of the rotaxane model. If the position of these atoms crossed, this indicated dethreading as the macrocycle moves over the stopper. If these atoms reach a plateau this indicates either unstoppering or no reaction as the macrocycle is unable to move past the stopper. We tracked the distance between the central O atom of the glycol chain of the macrocycle and either the Pd atom (in the case of $\mathbf{1_{Pd}}$) or the pyridine N atom (in the case of 1_H) to calculate the length of the macrocycle throughout the simulation. We tracked the distance between two phenolic O atoms of the macrocycle to calculate the width of the macrocycle throughout the simulation.

6.5.1 Molecular Dynamics Simulations of 1_{Pd}

Out of 20 molecular dynamics trajectories of model $\mathbf{1_{Pd}}$ at 3.00 nN, C-C bond scission was observed in 55 % of the trajectories while there was no reaction in the other 45 %. Trajectories 1, 2, 5, 7, 9-11, 16 and 17 resulted in no rotaxane dissociation. All other trajectories resulted in bond scission. Bond scission is evidenced by the movement of the atoms at the end of the stopper and centre of the macrocycle in the same direction and by a sharp peak in the energy profile at ≈ 1000 $kJmol^{-1}$. Comparing the length of the cavity size over the course of the simulation compared to the width of the stopper at 0 force (figures S12) shows that the cavity remains a similar size to the stopper (grey dashed line) at all forces. The width of the stopper is represented as an average of the stopper width of $\mathbf{1_{Pd}}$ and $\mathbf{1_H}$ at zero force. The width of the macrocycle decreases for all trajectories (figure S13).

Out of 20 molecular dynamics trajectories of $\mathbf{1_{Pd}}$ at 3.50 nN, C-C bond scission was observed in 100 % of the trajectories. A summary of the dissociation pathways and scission points is shown in tables S3 and S4.

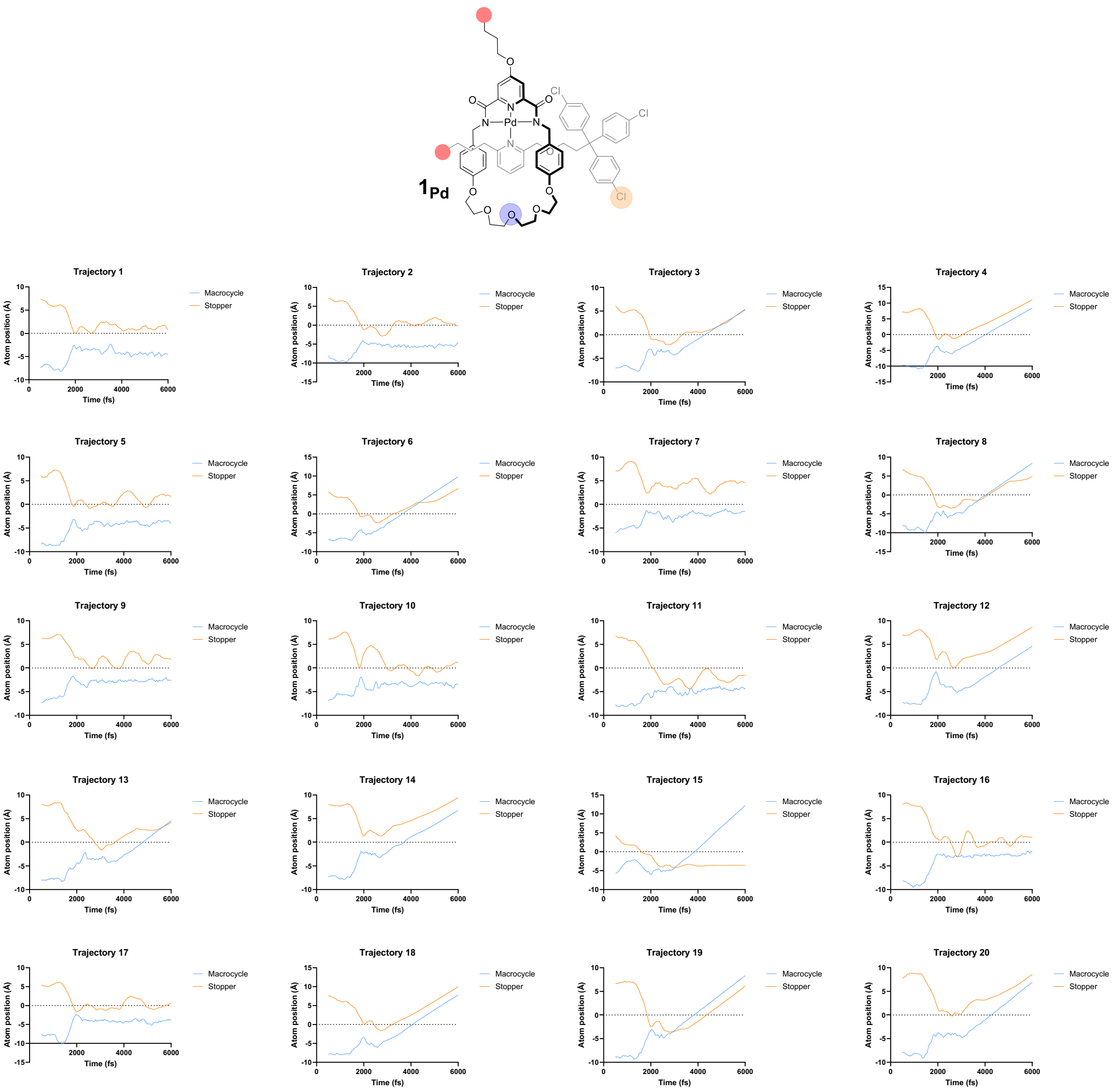

Figure S8. MD trajectories for $\mathbf{1_{Pd}}$ @ 3.0 nN

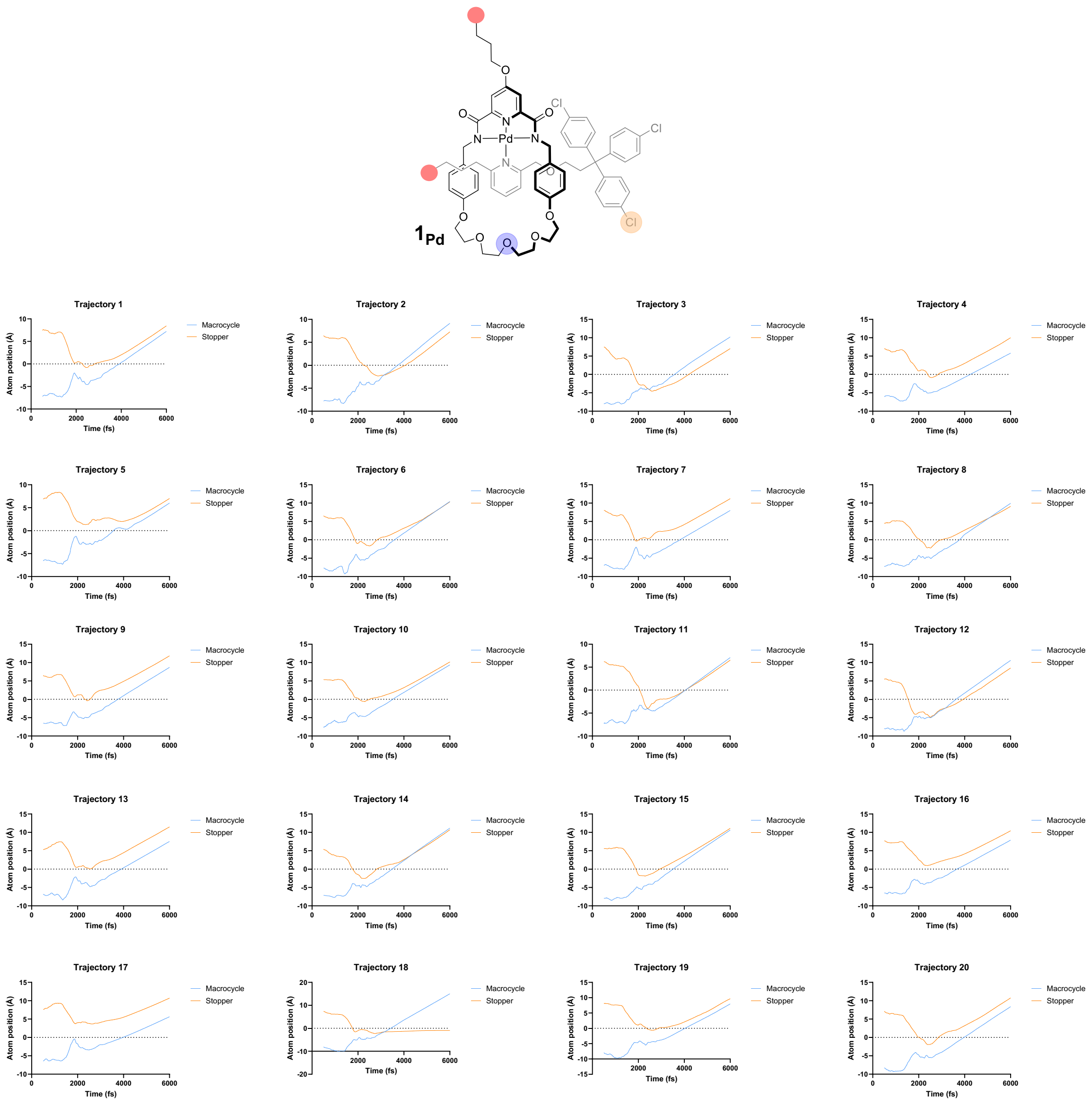

Figure S9. MD trajectories for $\mathbf{1_{Pd}}$ @ 3.5 nN

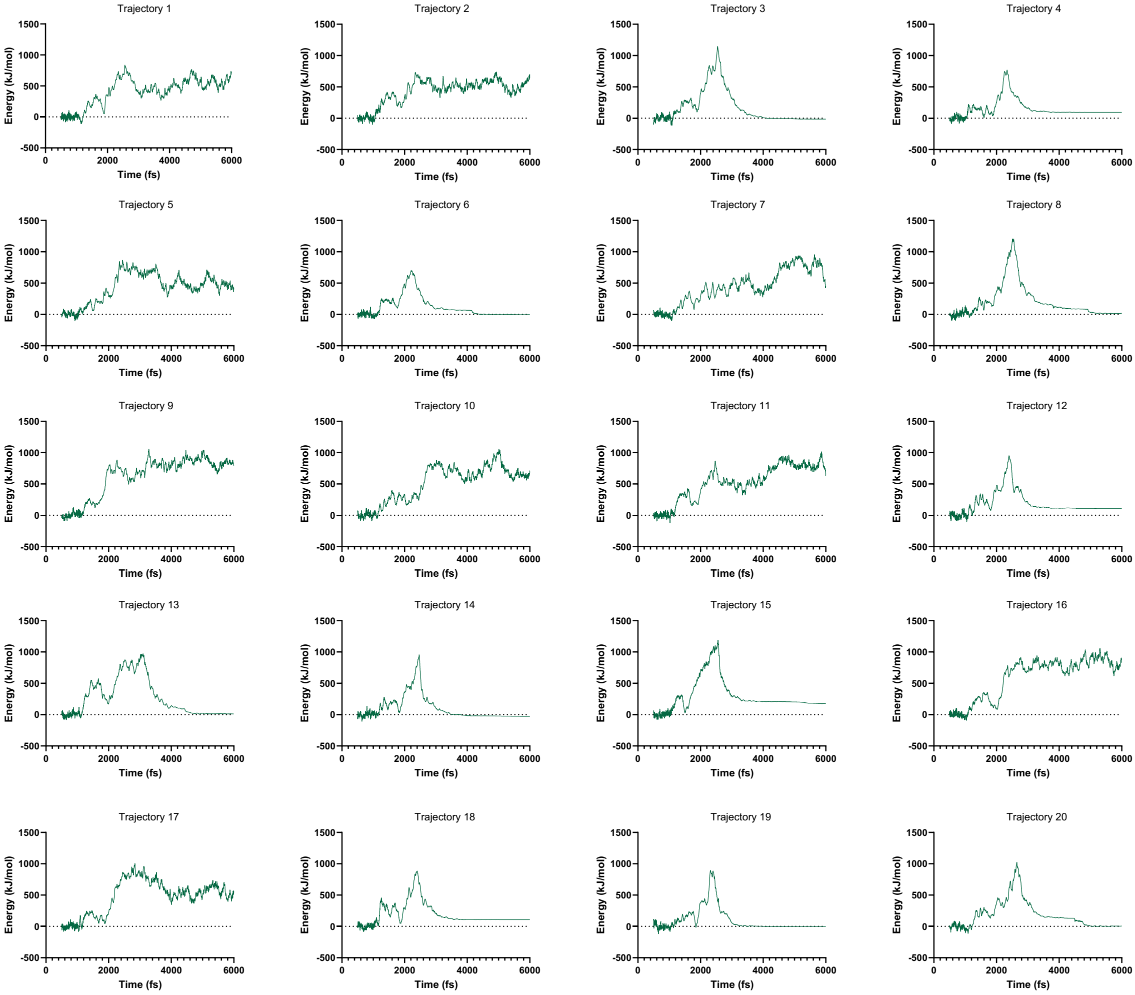

Figure S10. Energy profiles for each MD trajectory @ 3.00 nN

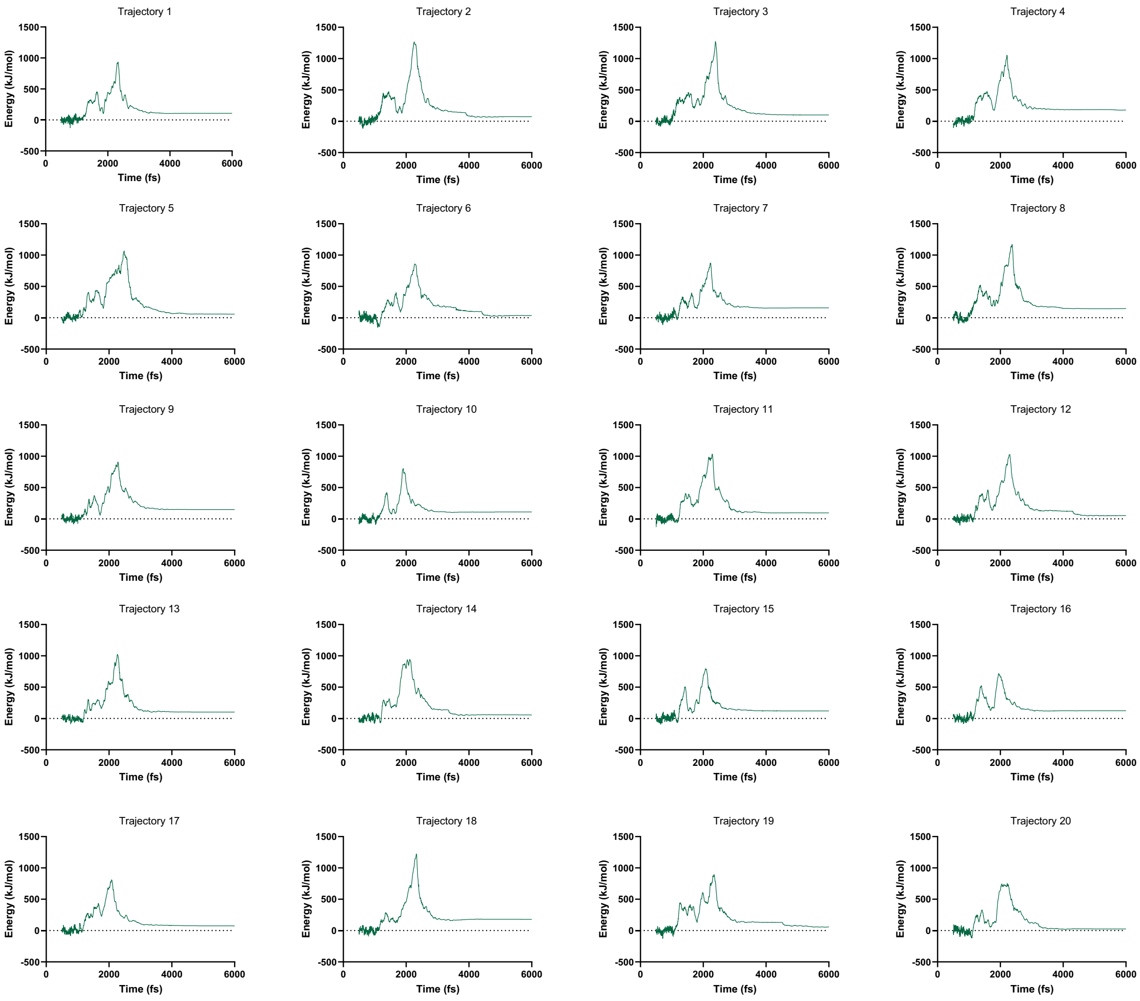

Figure S11. Energy profiles for each MD trajectory @ 3.50 nN

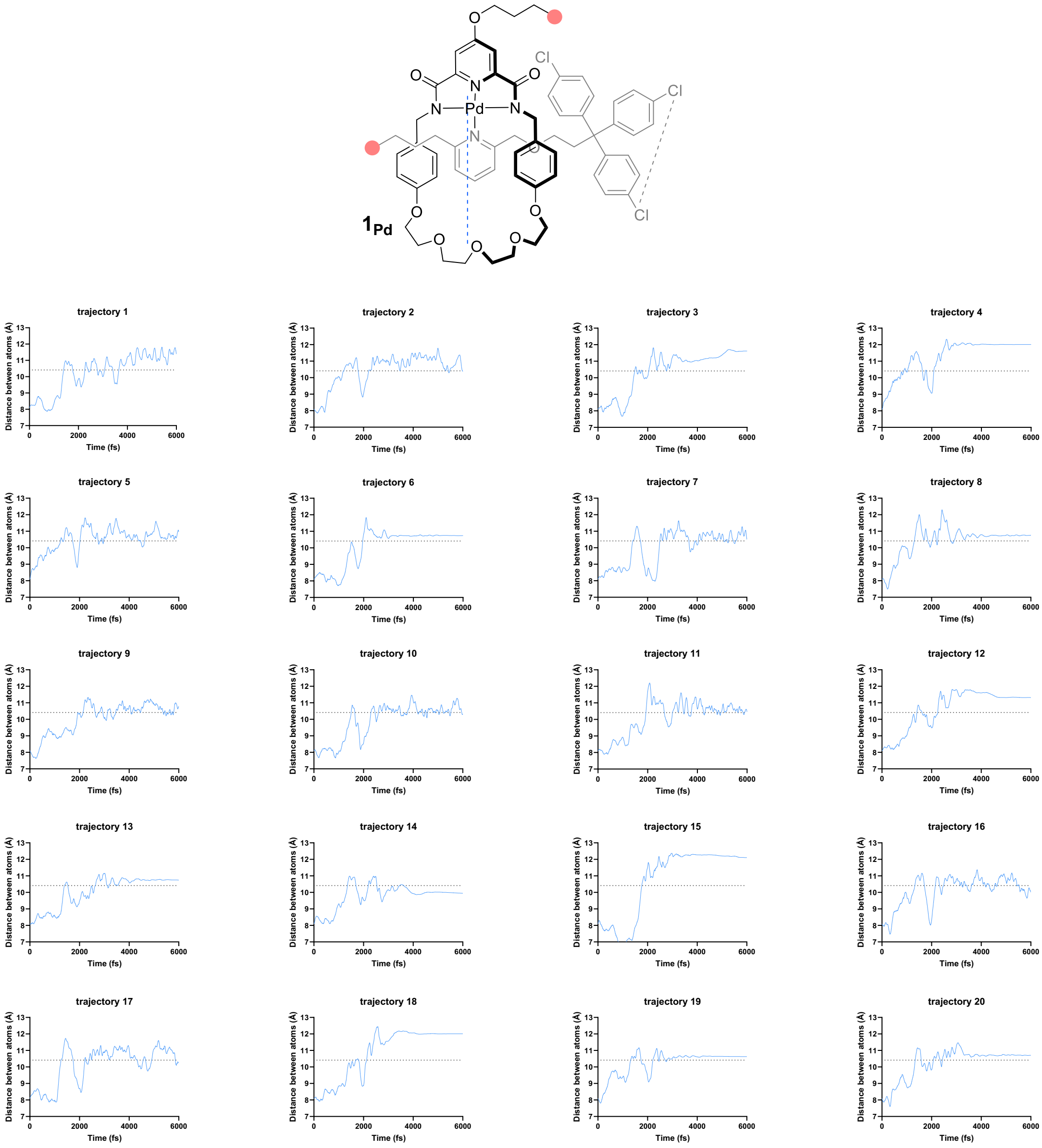

Figure S12. Calculation of cavity length of $\mathbf{1_{Pd}}$ @ 3.0 nN

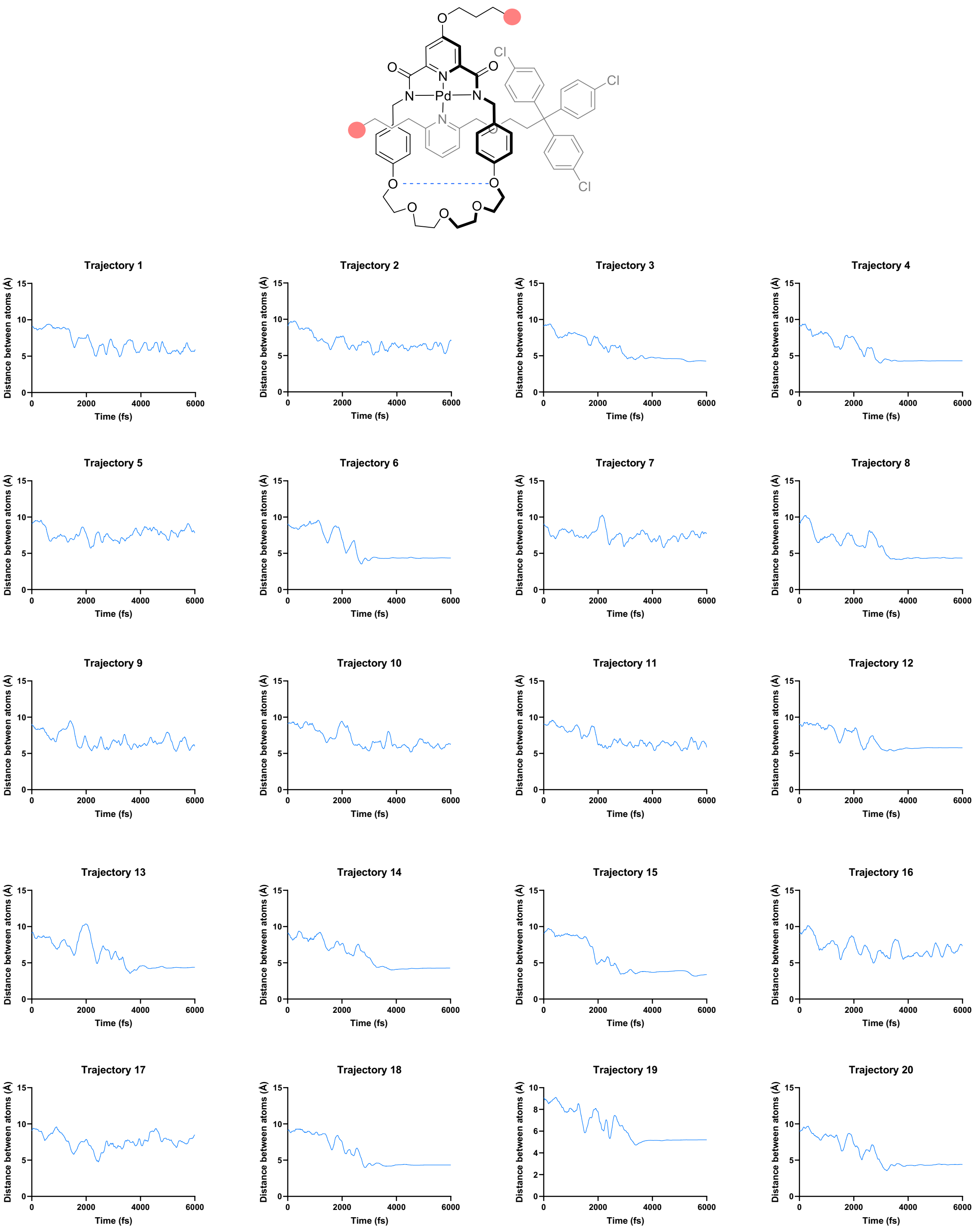

Figure S13. Cavity width of $\mathbf{1_{Pd}}$ @ 3.0 nN

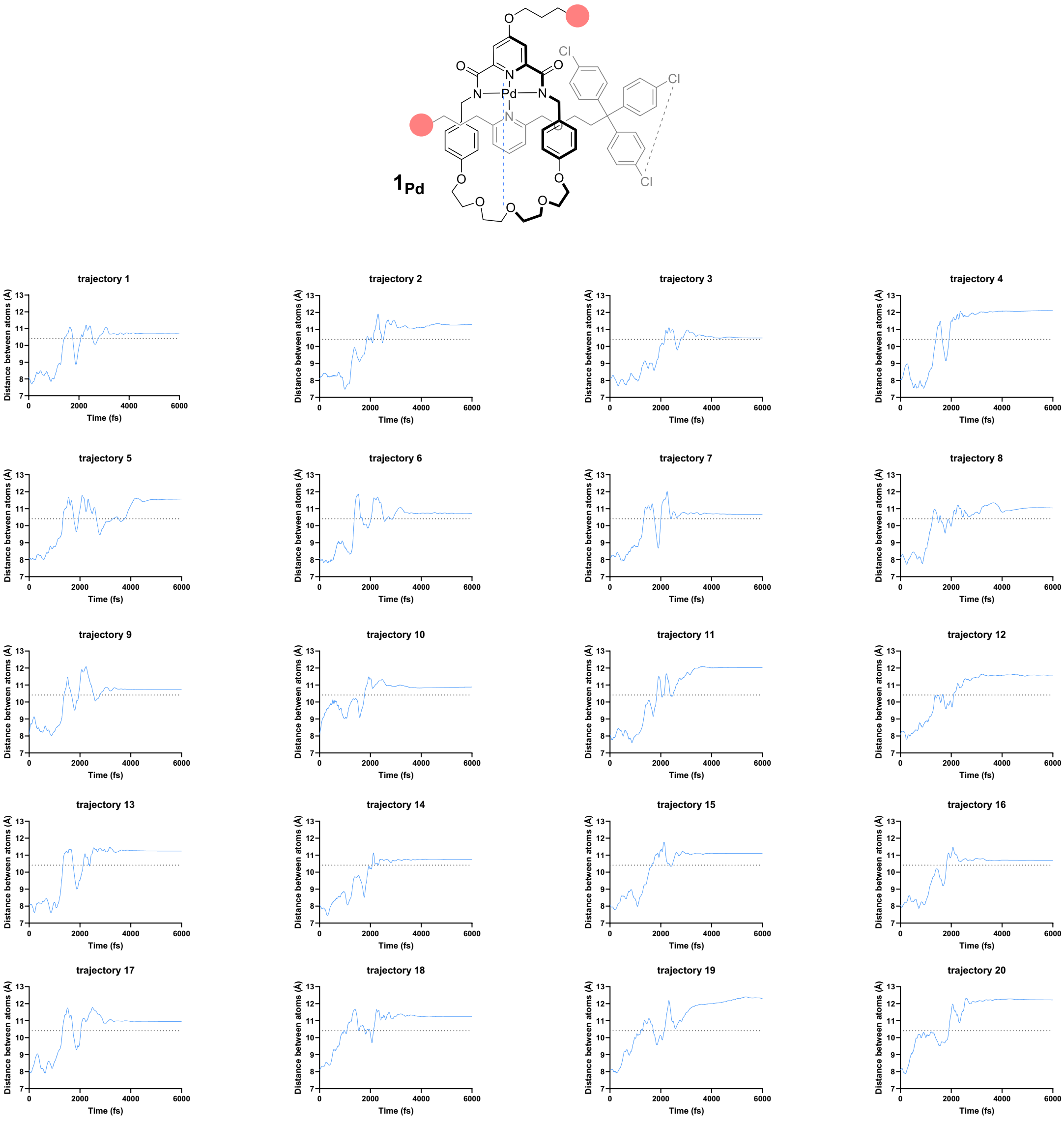

Figure S14. Calculation of cavity length of $\mathbf{1}_{\mathbf{Pd}}$ @ 3.5 nN

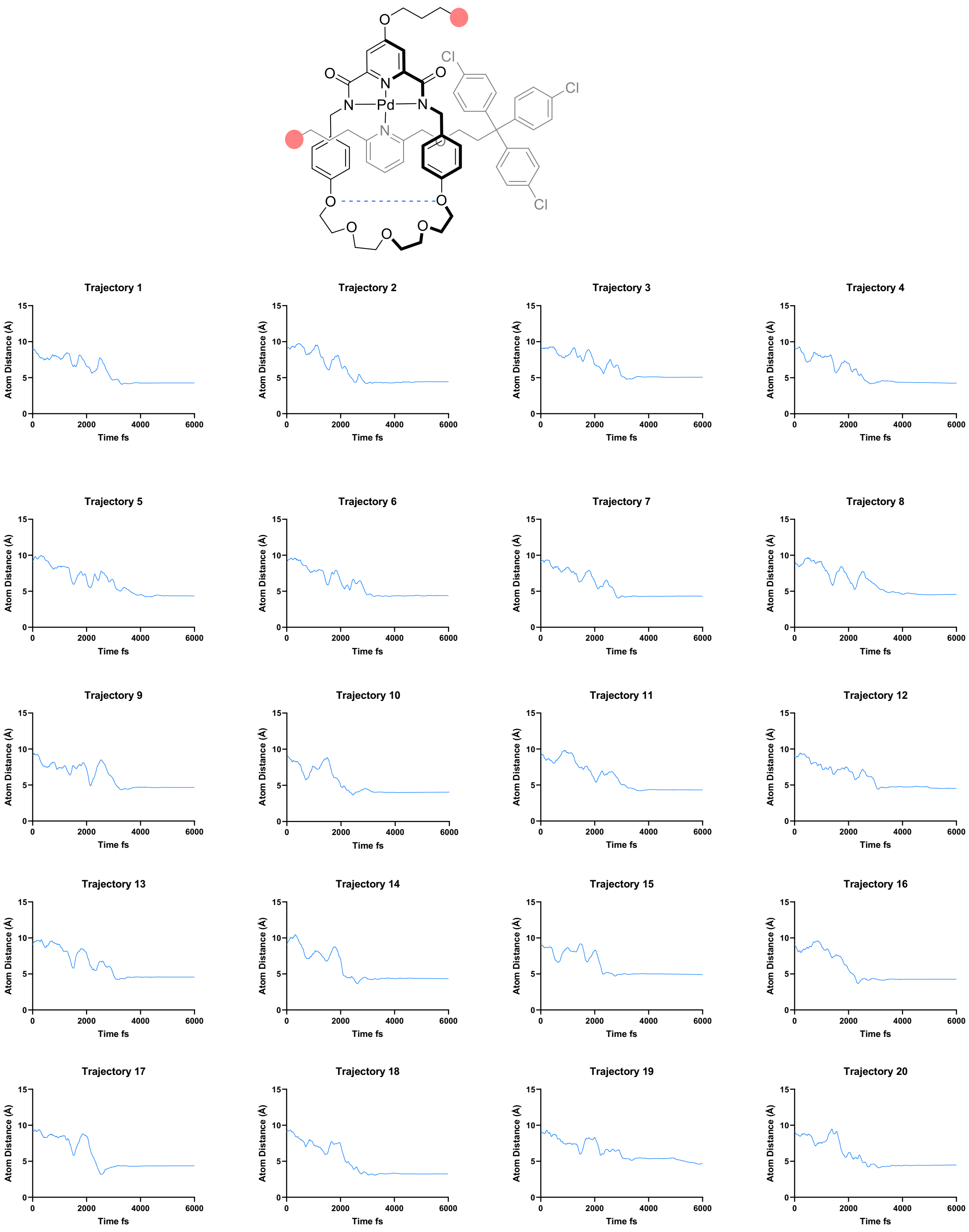

Figure S15. Calculation of cavity width of $\mathbf{1}_{\mathbf{Pd}}$ @ 3.5 nN

6.5.2 Molecular Dynamics Simulations of 1_H

Out of 20 molecular dynamics trajectories of model **1_H** at 3.00 nN, C-C bond scission was observed in 20 % of the trajectories, dethreading in 40 % of trajectories and no reaction in 35 % of trajectories. Trajectories 1, 5, 8, 9, 11, 12 and 16 resulted in no reaction. Trajectories 2, 6, 10, 15 and 17 resulted in bond scission, trajectories 3, 4, 7, 13, 14, 18, 19 and 20 resulted in dethreading. Dethreading can be observed by the atoms at the need of the stopper and the centre of the macrocycle moving in opposite directions and by a shallow rounded energy profile peaking at ≈ 500 kJmol^{-1}. Comparing the length of the cavity size over the course of the simulation compared to the width of the stopper at 0 force (Figures S20 and S22) shows that the cavity is able to increase in size, longer than the length of the stopper (grey dashed line), as the rotaxane is stretched. The width of the macrocycle is represented as a U-shape for trajectories that show dethreading behaviour as the macrocycle contracts as it lengthens then expands over the stopper (figures S19 and S21). For trajectories which show unstoppering behaviour or no reaction the macrocycle narrows over the entire time period.

Out of 20 molecular dynamics trajectories of model rotaxane at 3.50 nN, C-C bond scission was observed in 80 % of the trajectories and dethreading in 20 % of trajectories. Trajectories 1-5, 8, 9, 11, 12, and 14-20 resulted in bond scission, trajectories 6, 7, 10 and 13 resulted in dethreading.

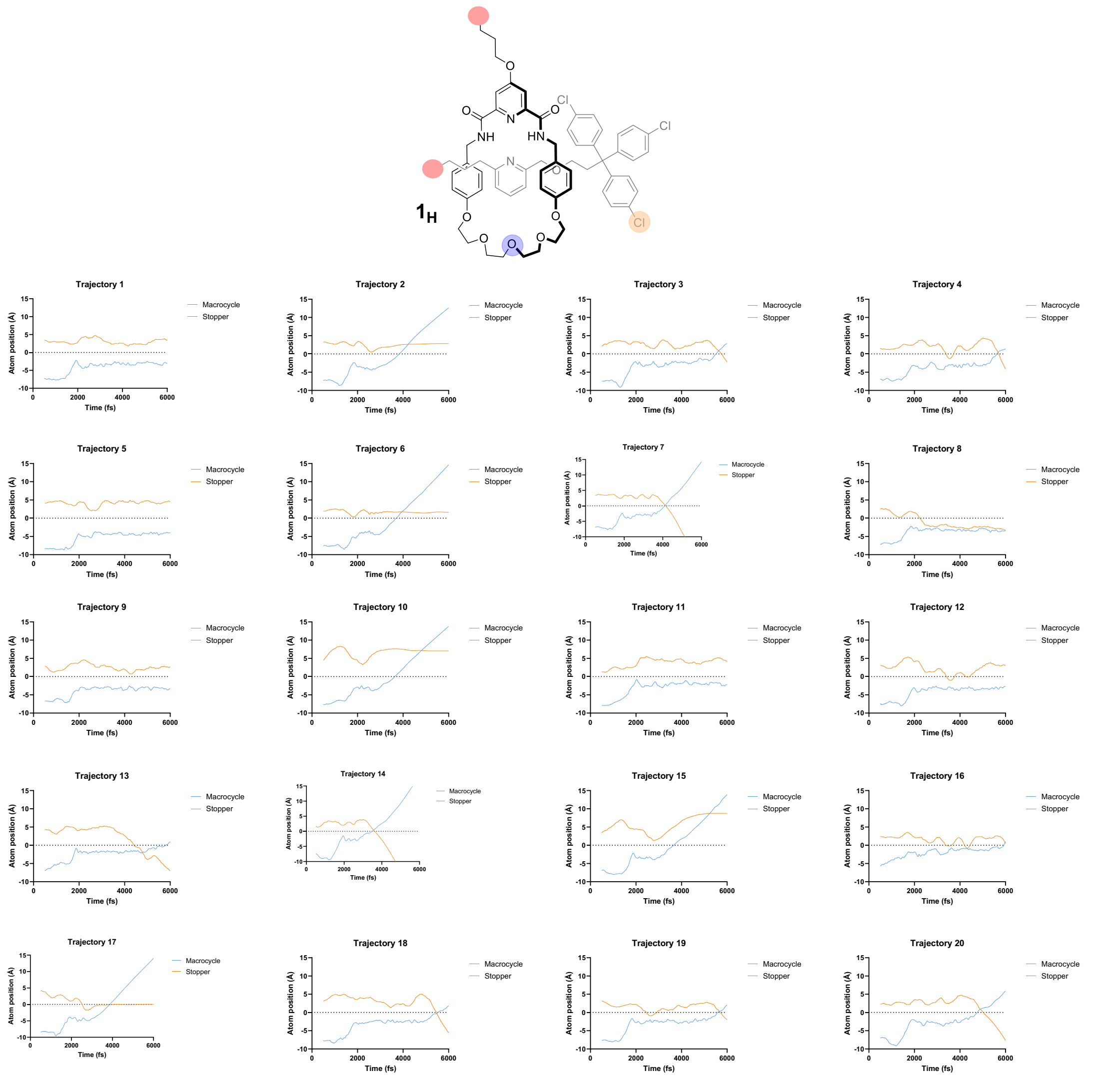

Figure S16. MD trajectories for **1_H**@ 3.00 nN

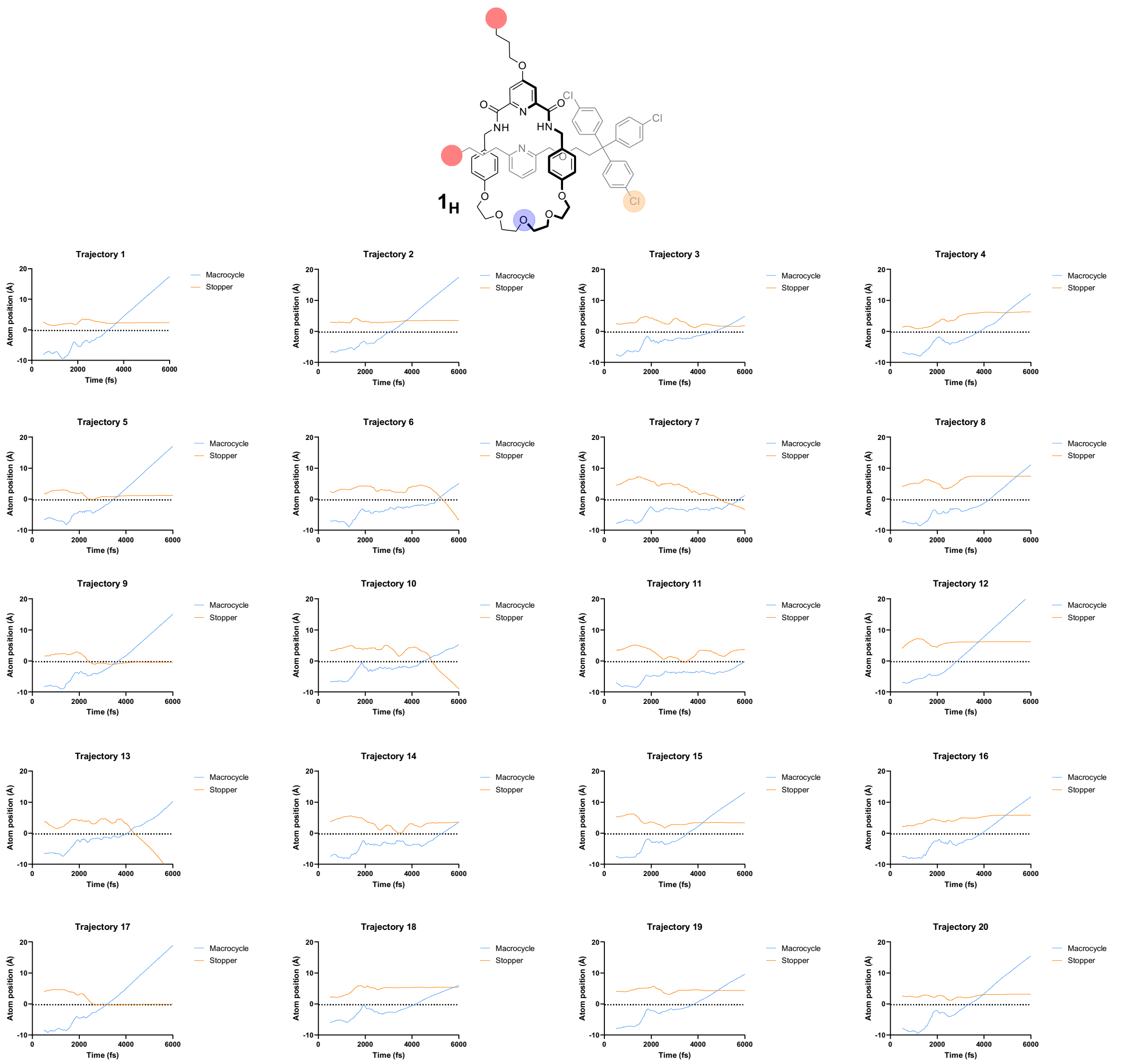

Figure S17. MD trajectories for $\mathbf{1_H}$ @ 3.50 nN

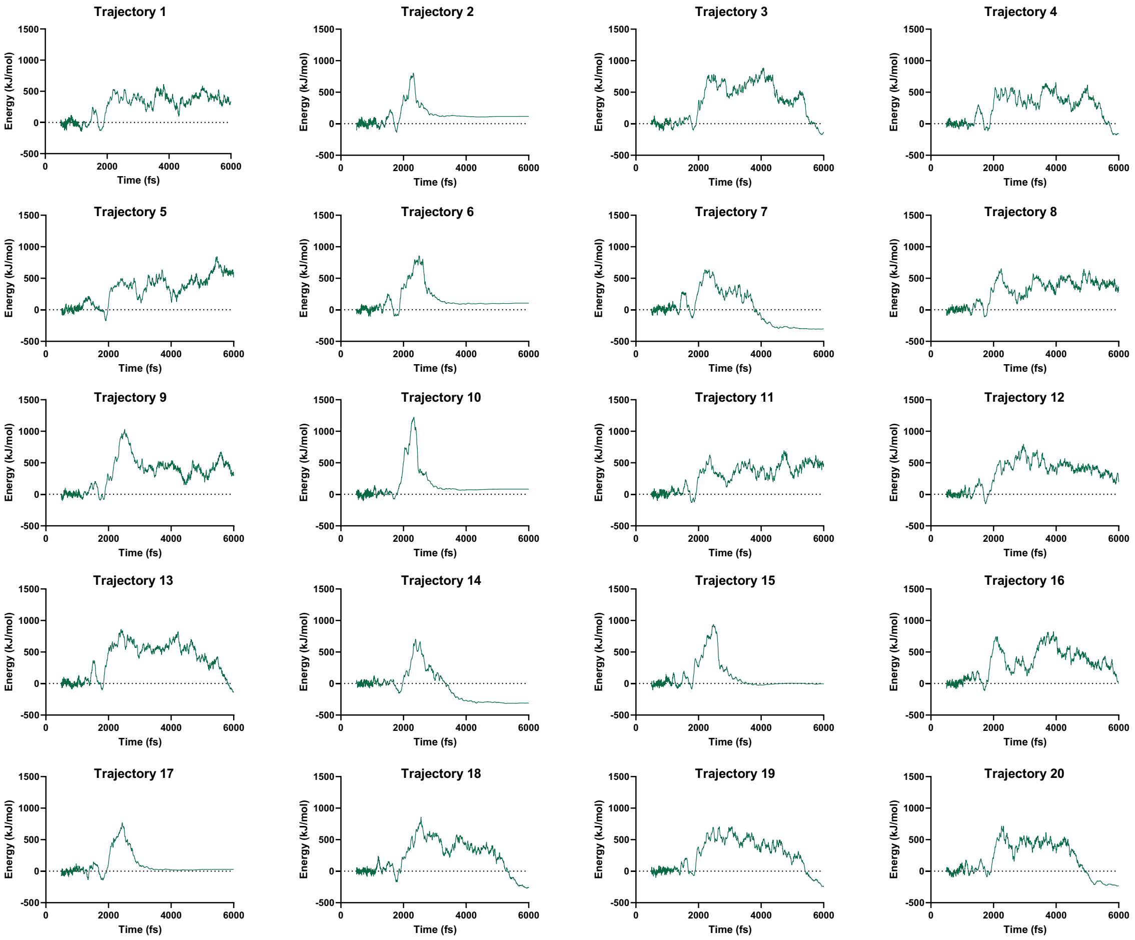

Figure S18. Energy profiles from MD calculations @ 3.0 nN

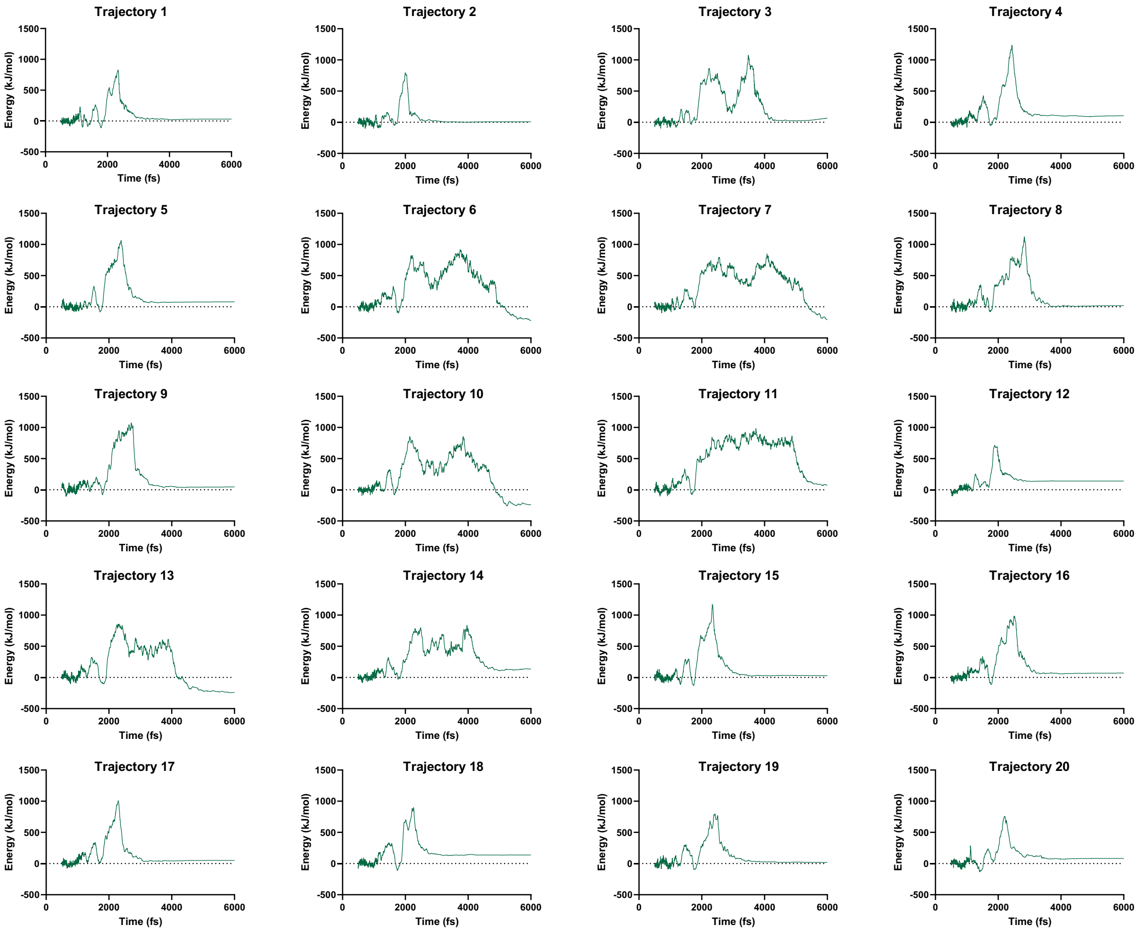

Figure S19. Energy profiles for MD trajectories @ 3.5 nN

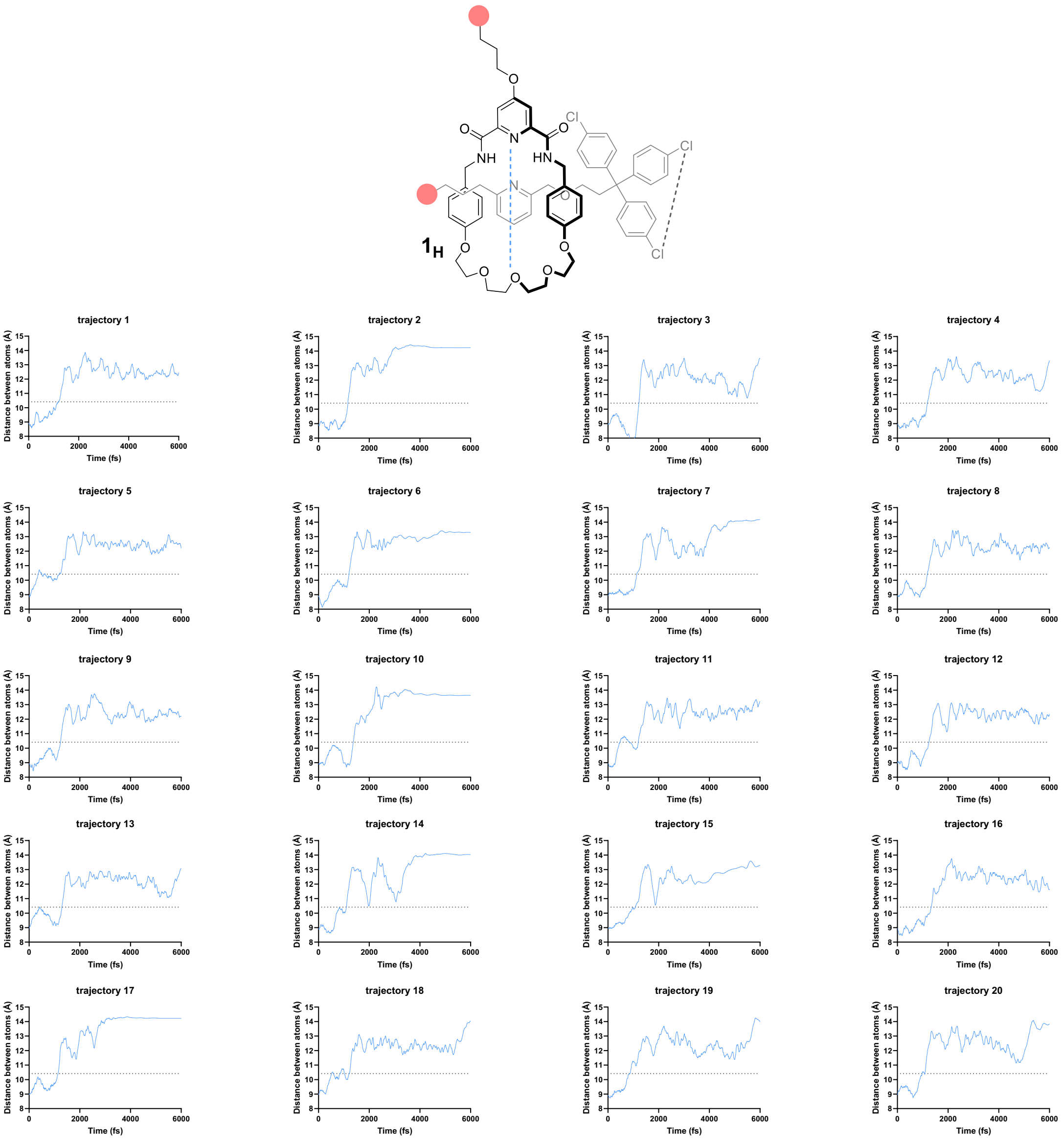

Figure S20. Calculation of cavity length of $\mathbf{1_H}$ @ 3.0 nN

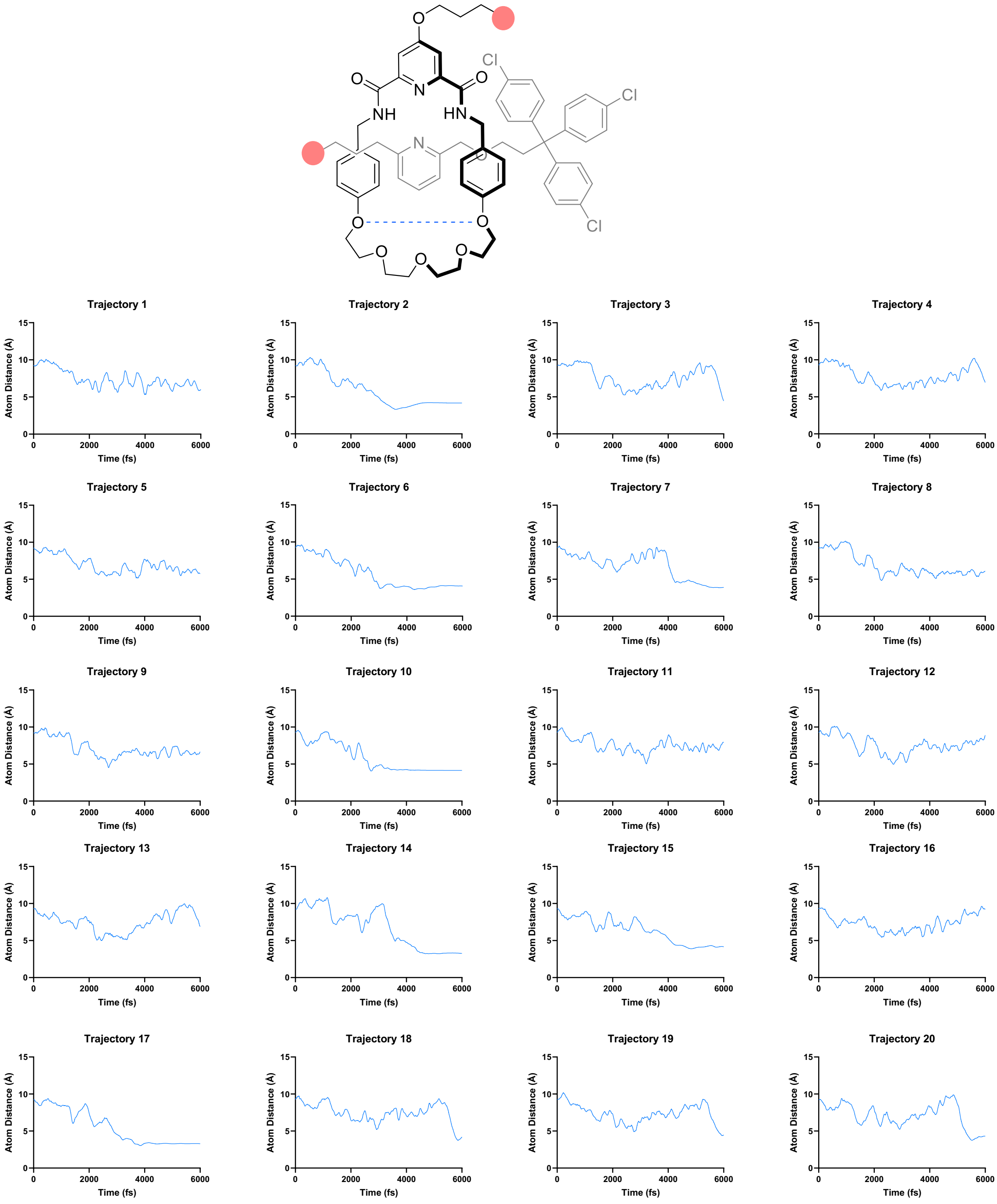

Figure S21. Calculation of cavity width of $\mathbf{1_H}$ @ 3.0 nN

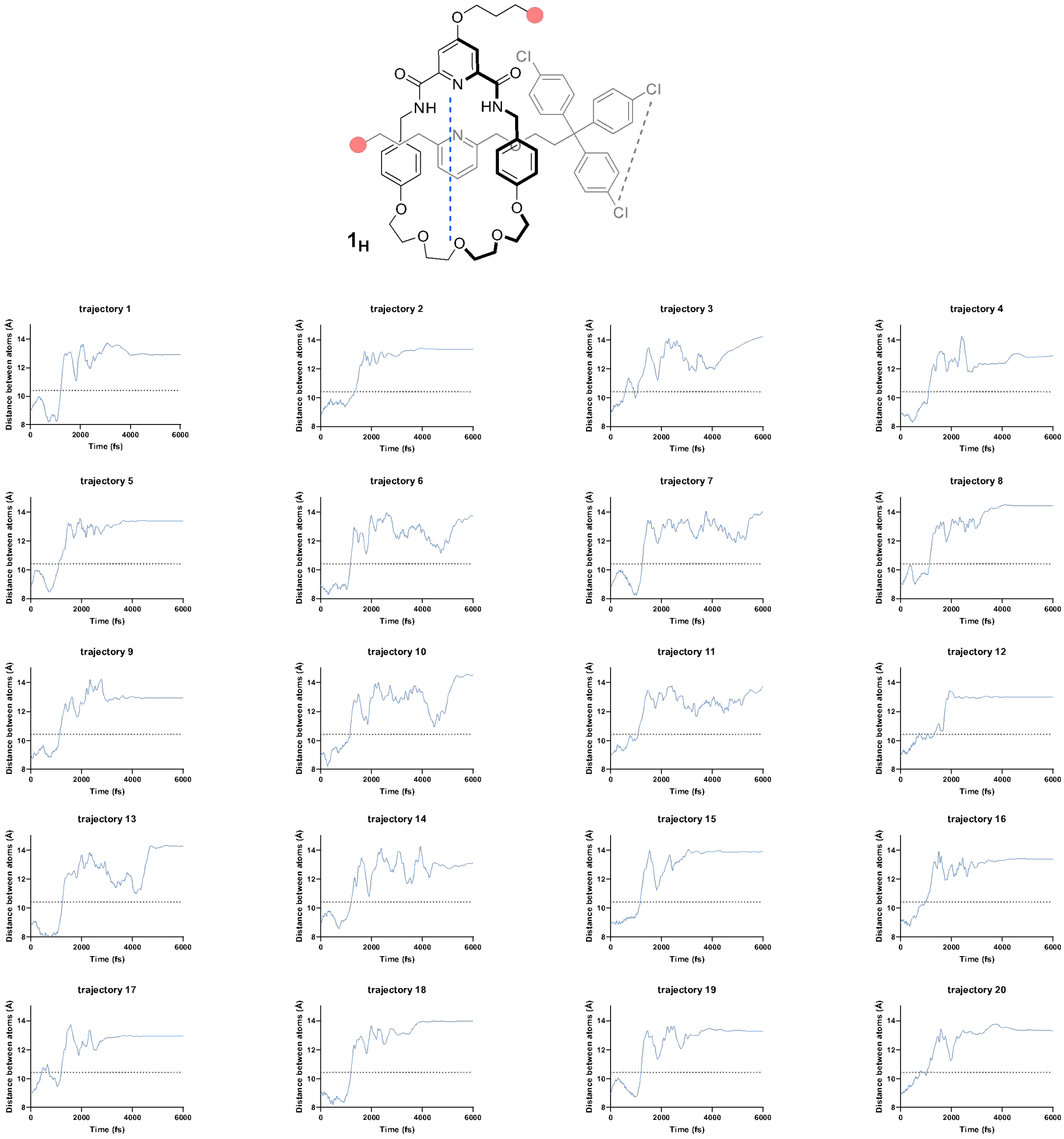

Figure S22. Calculation of cavity length of $\mathbf{1_H}$ @ 3.5 nN

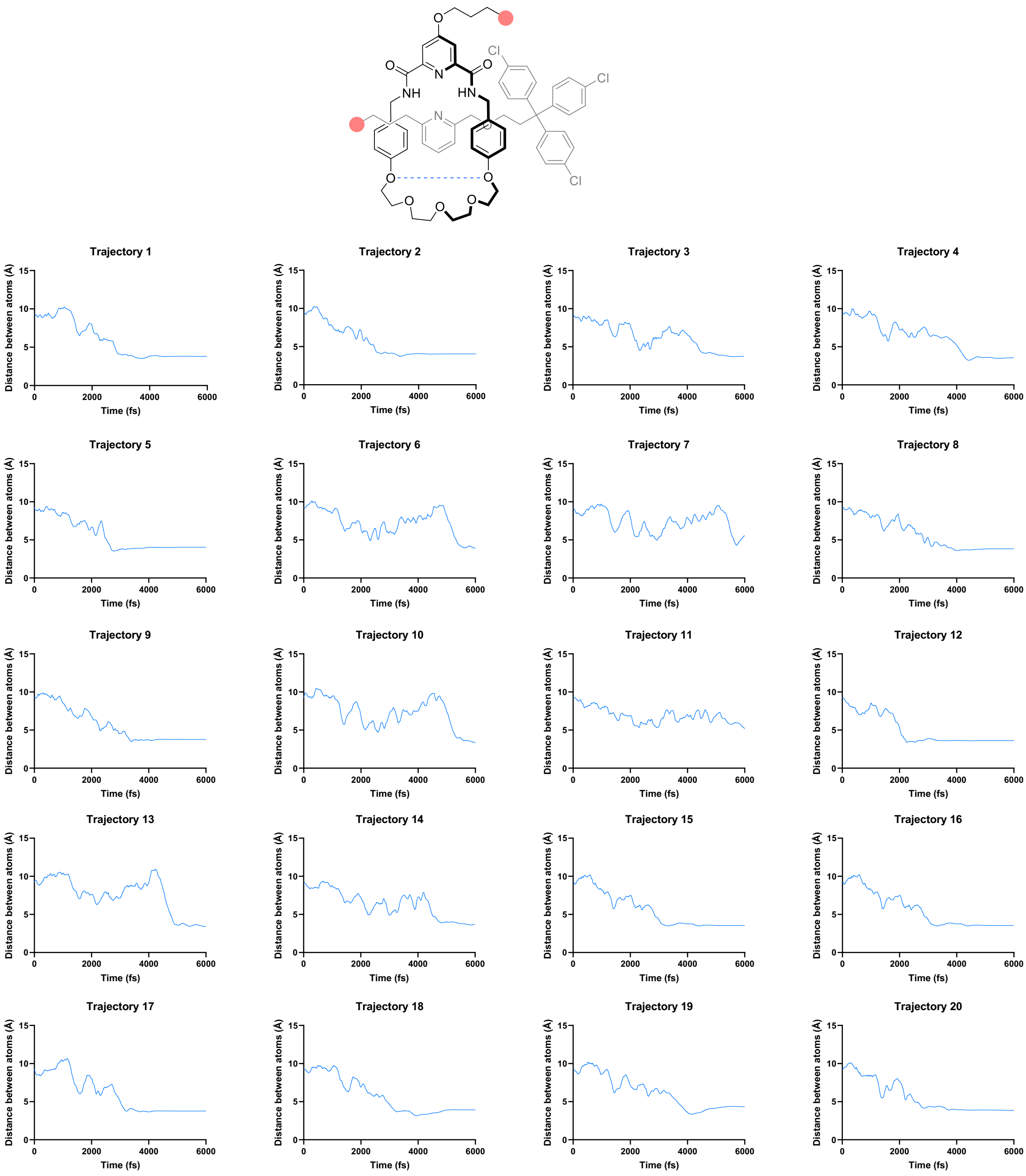

Figure S23. Calculation of cavity width of $\mathbf{1_H}$ @ 3.5 nN

Table S3. Summary of MD calculation results

Mechanophore	Force (nN)	Unstoppering (%)	Dethreading (%)	No reaction (%)
1-Pd	3.0	60	0	40
	3.5	100	0	0
1-H	3.0	25	45	30
	3.5	80	20	0

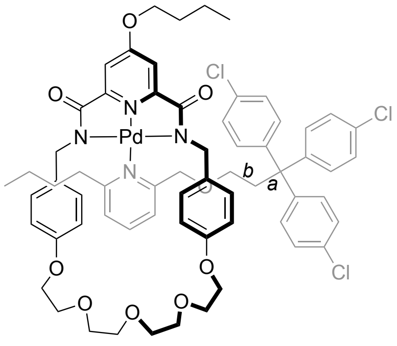

Table S4. Summary of bond scission sites

Mechanophore	Force (nN)	Scission at *a* (%)	Scission at *b* (%)
**1$_{Pd}$**	3.0	25	75
	3.5	15	85
**1$_{H}$**	3.0	20	80
	3.5	31	69

7 NMR spectra

7.1 Small molecule NMR spectra

7.1.1 Spectra of S2

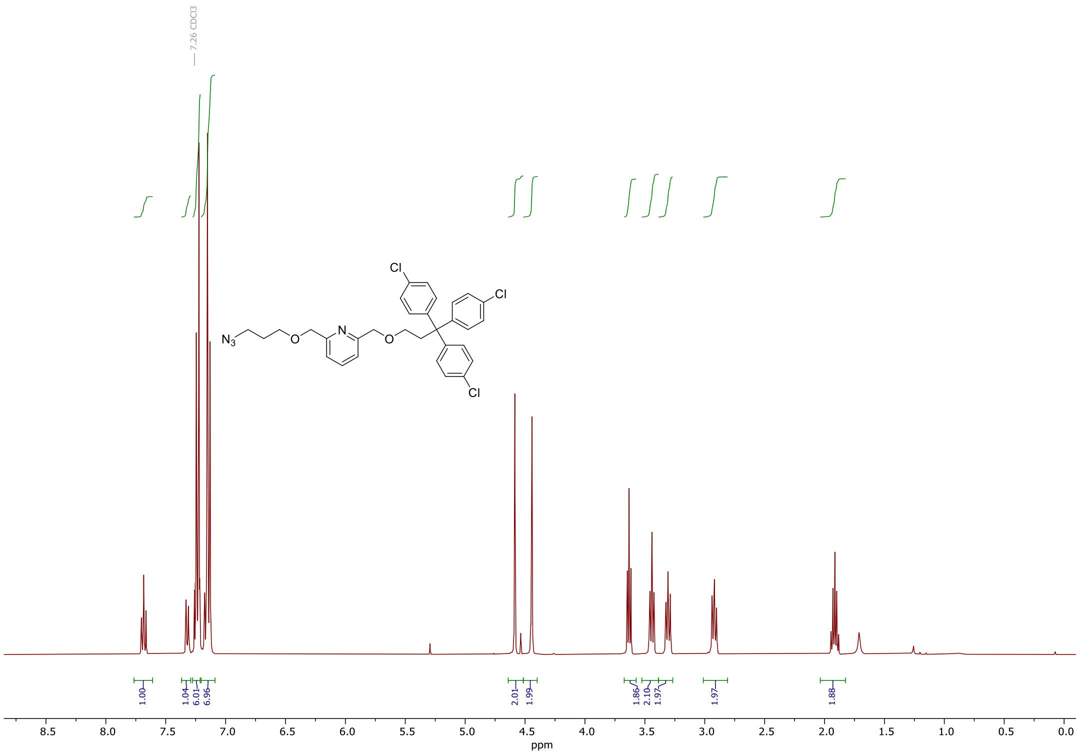

Spectrum S1. ^{1}H NMR (400 MHz, $CDCl_3$, 298 K) spectrum of compound **S2**

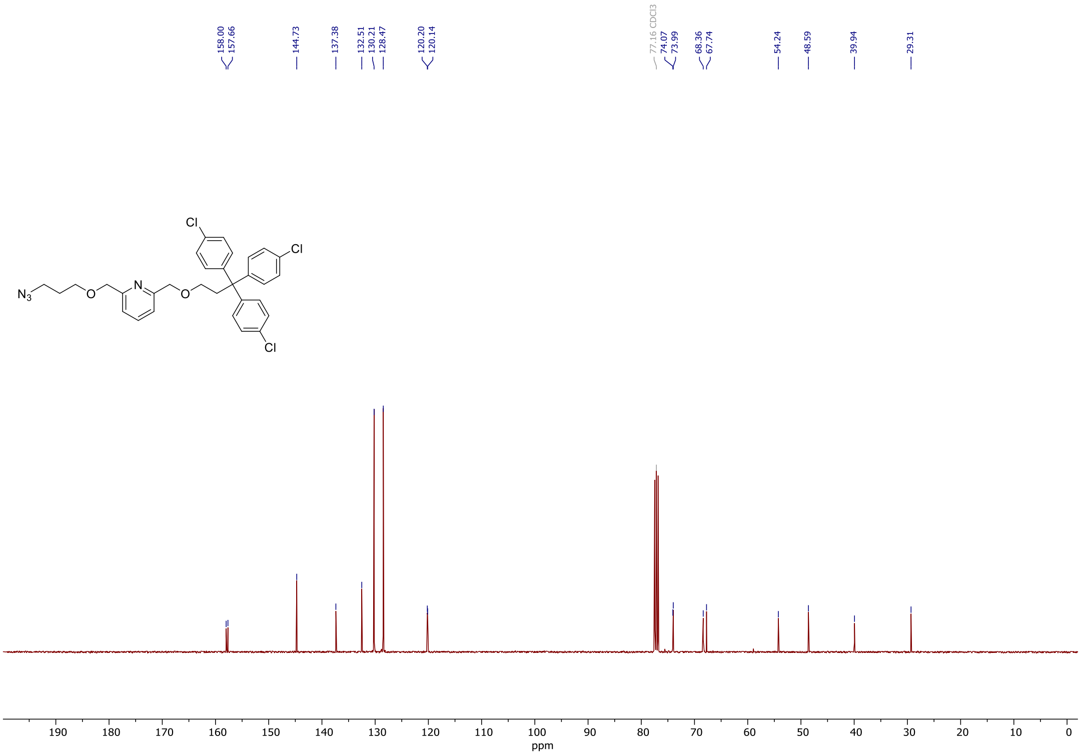

Spectrum S2. ^{13}C NMR (101 MHz, $CDCl_3$, 298 K) spectrum of compound **S2**

7.1.2 Spectra of S3

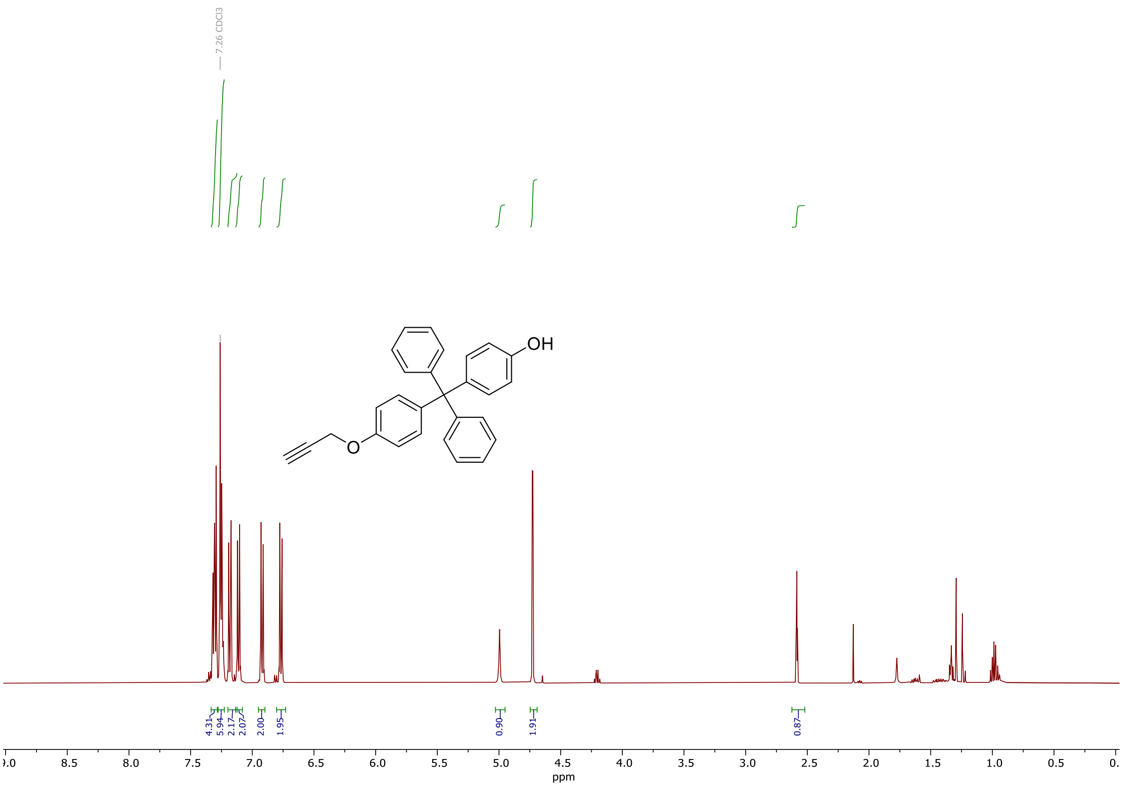

Spectrum S3. ^{1}H NMR (500 MHz, $CDCl_3$, 298 K) spectrum of compound **S3**

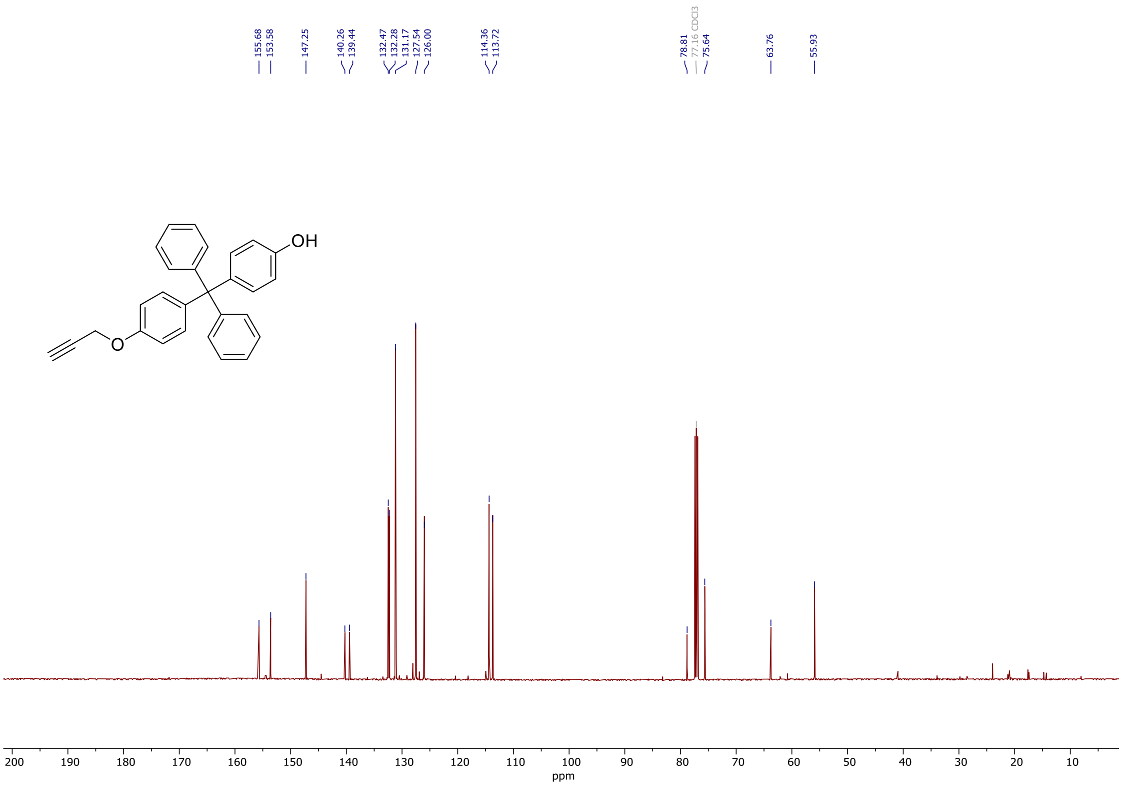

Spectrum S4. ^{13}C NMR (126 MHz, $CDCl_3$, 298 K) spectrum of compound **S3**

7.1.3 Spectra of S4

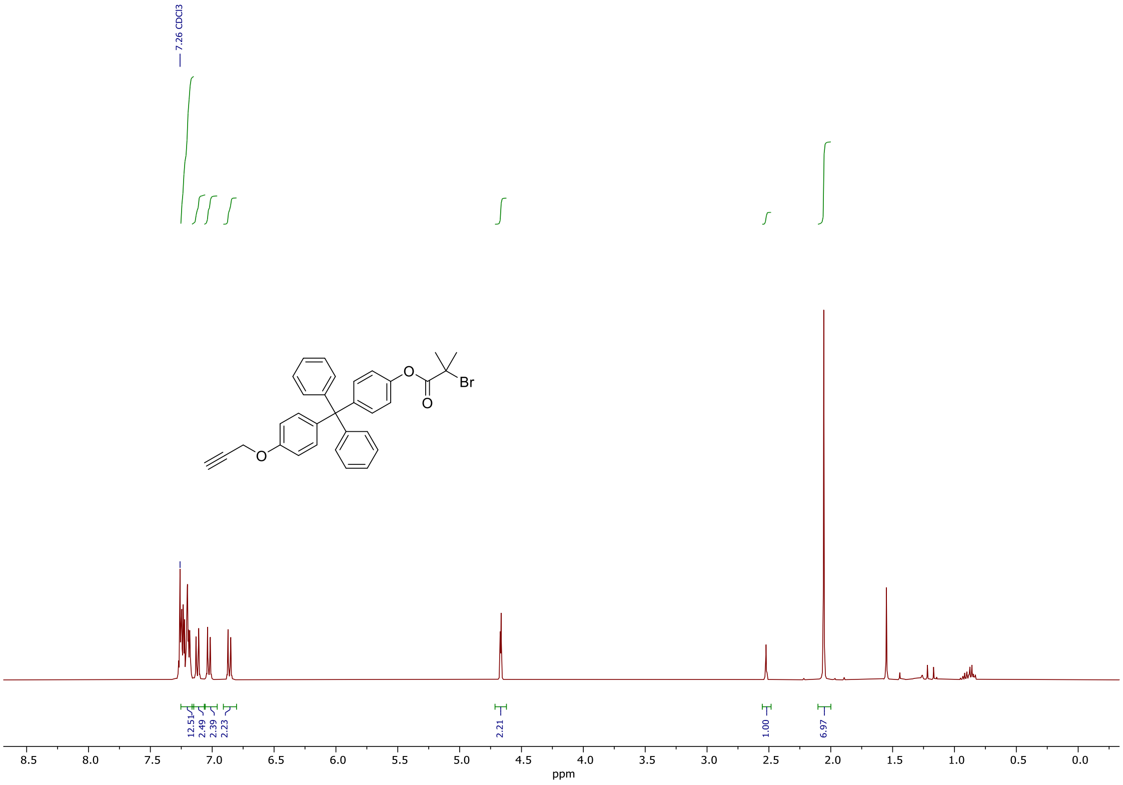

Spectrum S5. ^{1}H NMR (400 MHz, $CDCl_3$, 298 K) spectrum of compound **S4**

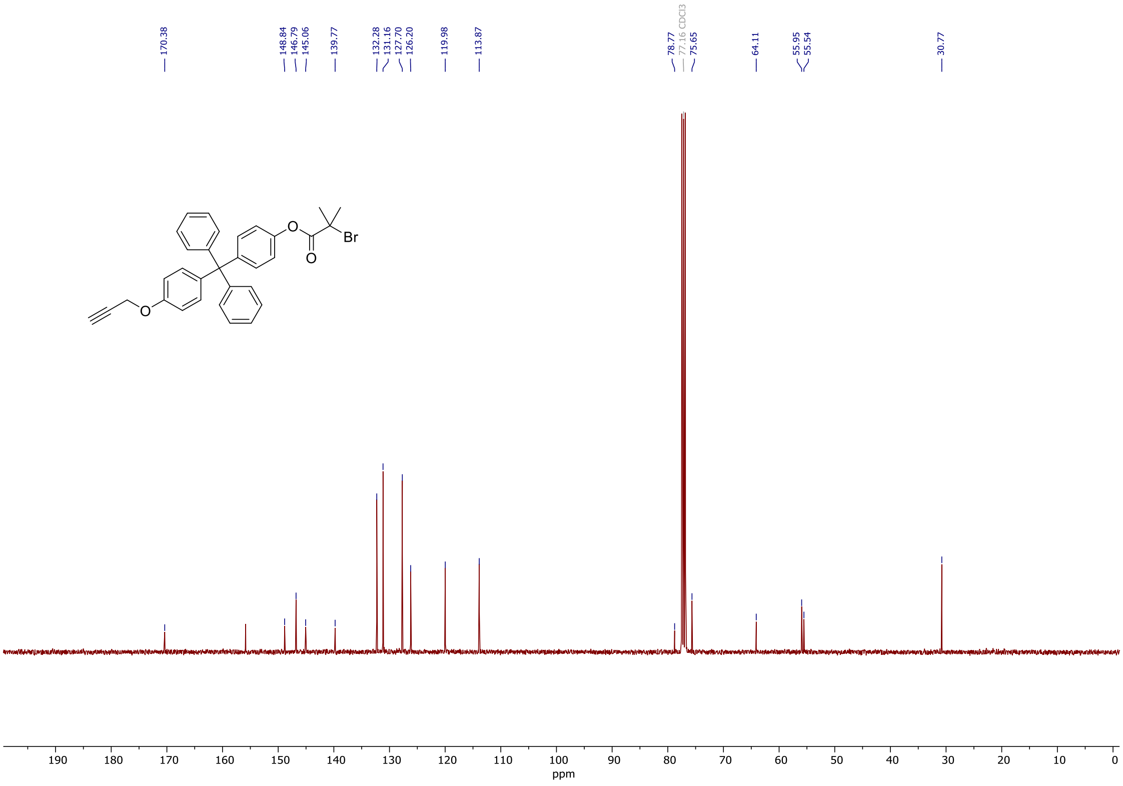

Spectrum S6. ^{13}C NMR (101 MHz, $CDCl_3$, 298 K) spectrum of compound **S4**

7.1.4 Spectra of S5

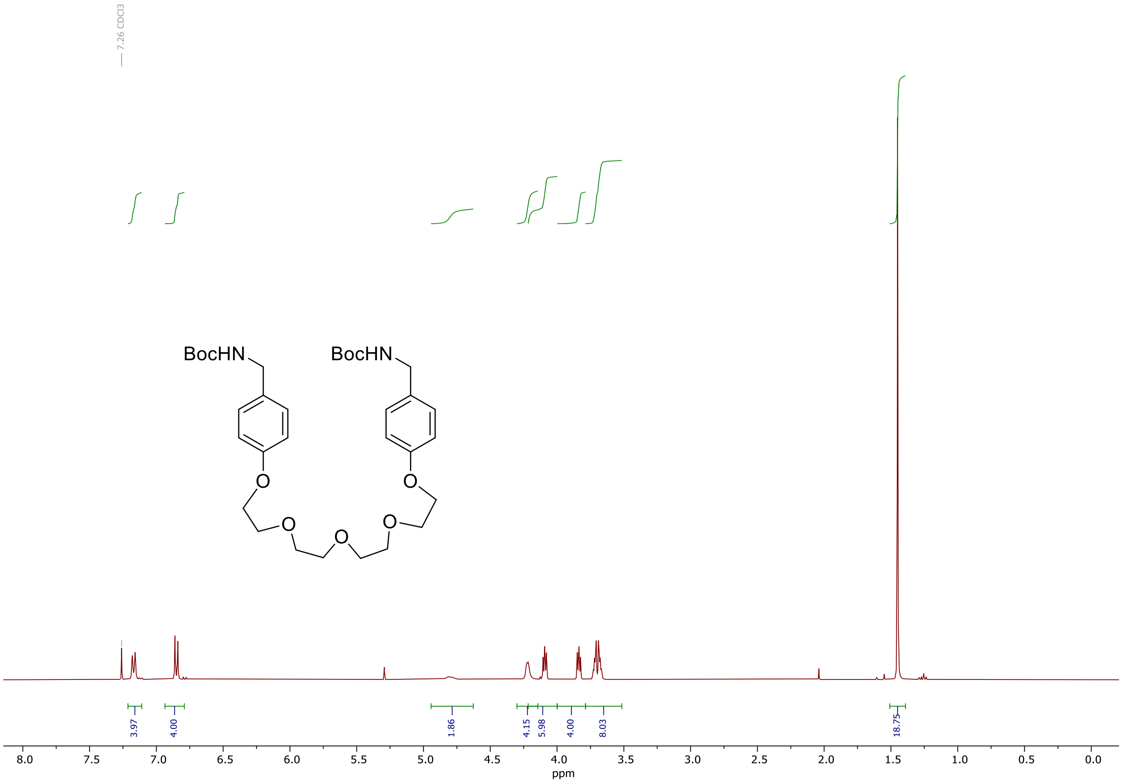

Spectrum S7. ^{1}H NMR (400 MHz, $CDCl_3$, 298 K) of compound **S5**

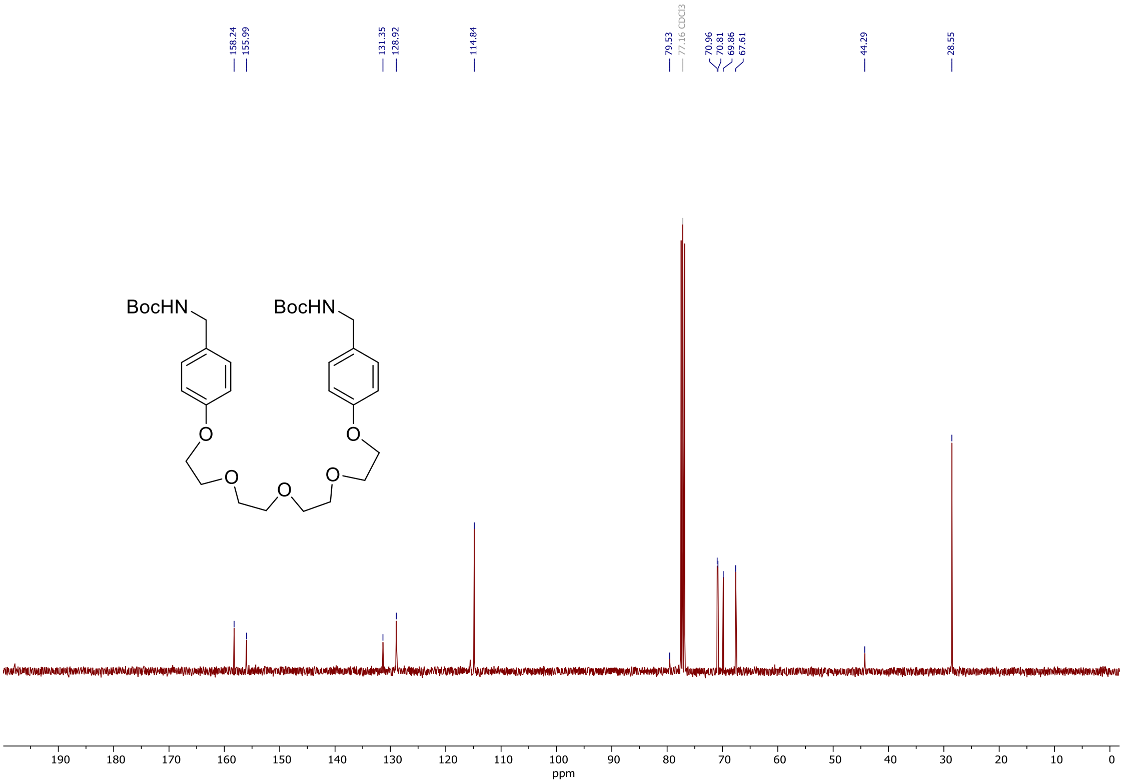

Spectrum S8. ^{13}C NMR (101 MHz, $CDCl_3$, 298 K) of compound **S5**

7.1.5 Spectra of S6

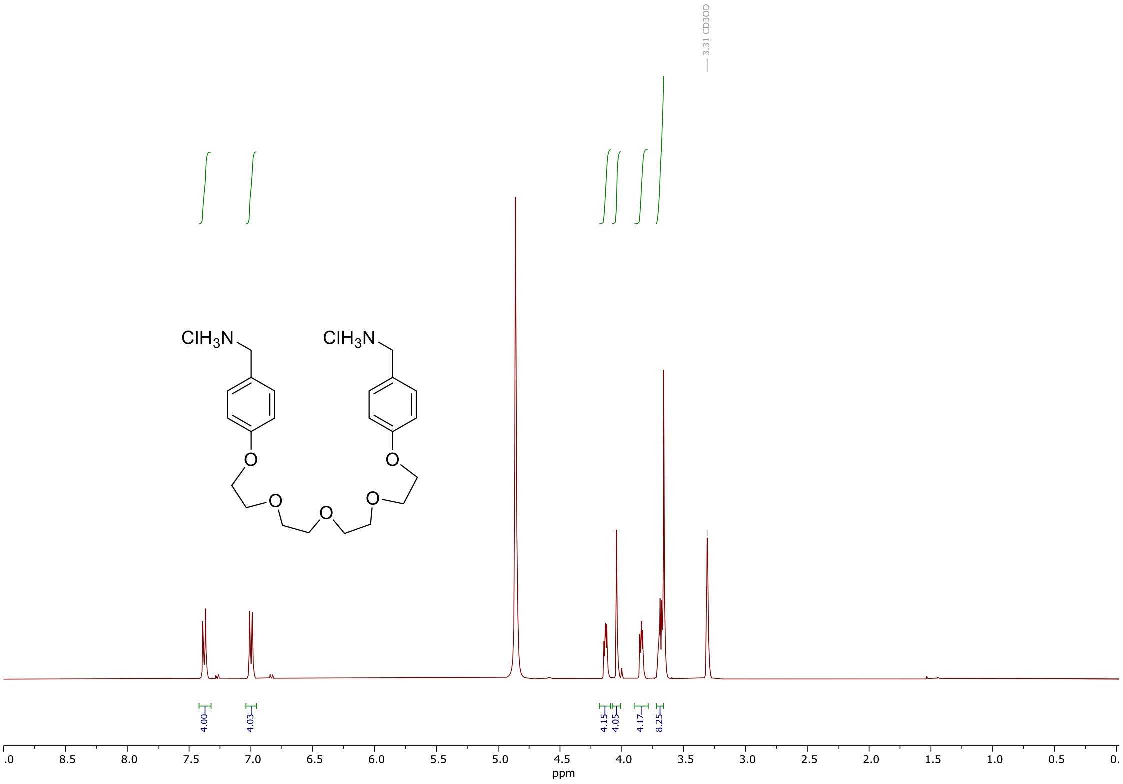

Spectrum S9. ^{1}H NMR (400 MHz, MeOD, 298 K) of compound **S6**

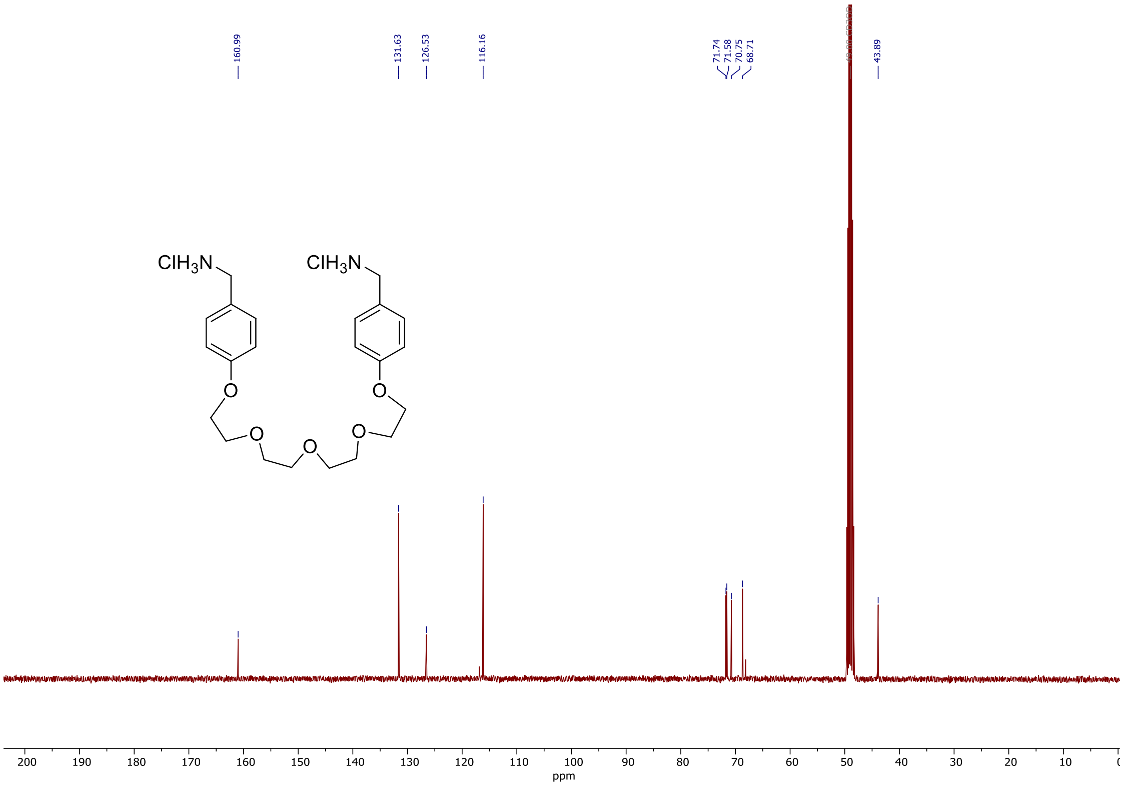

Spectrum S10. ^{13}C NMR (101 MHz, MeOD, 298 K) of compound **S6**

7.1.6 Spectra of S8

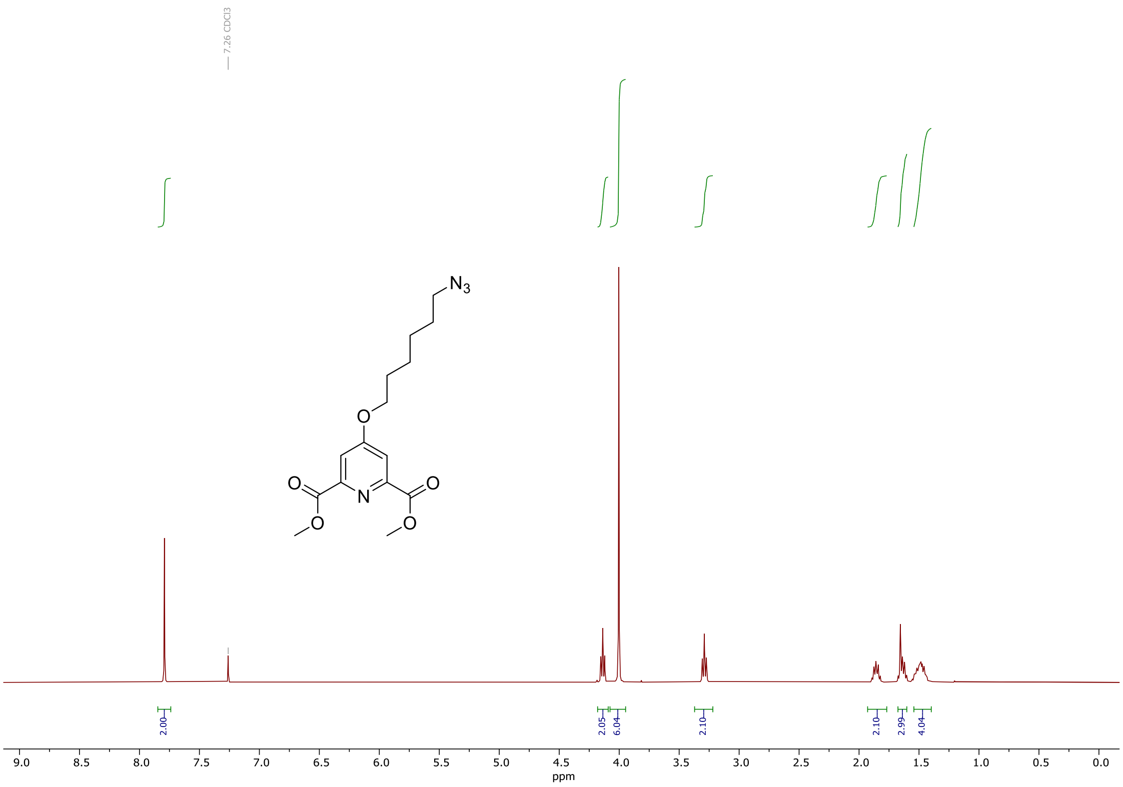

Spectrum S11. ^{1}H NMR (400 MHz, $CDCl_3$, 298 K) of compound **S8**

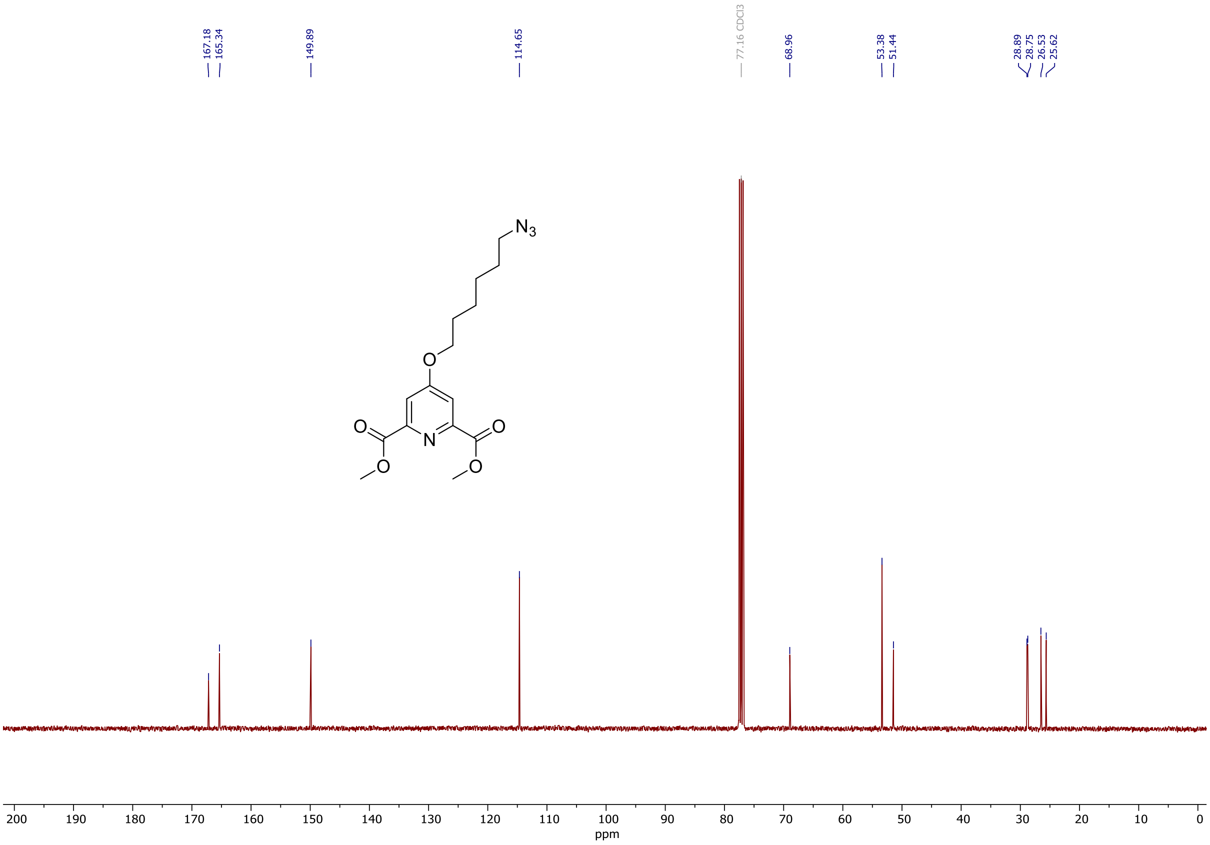

Spectrum S12. ^{13}C NMR (101 MHz, $CDCl_3$, 298 K) of compound **S8**

7.1.7 Spectra of S9

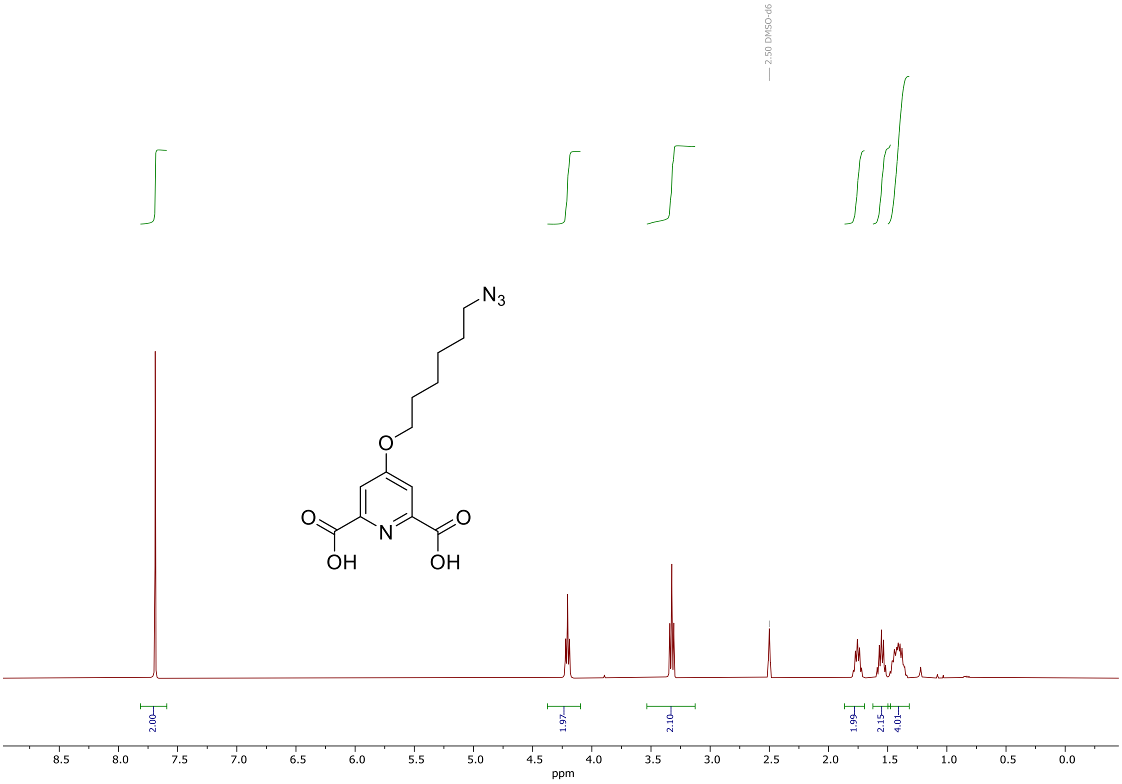

Spectrum S13. ^{1}H NMR (400 MHz, DMSO-d_6, 298 K) of compound **S9**

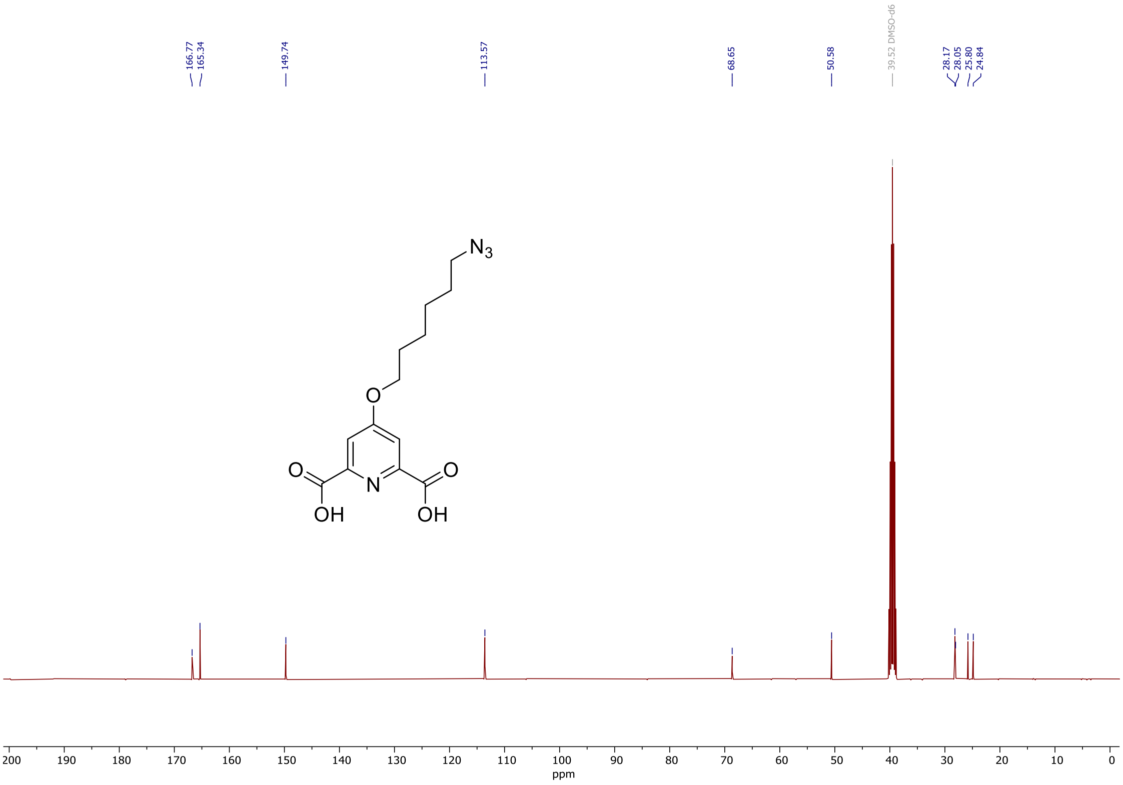

Spectrum S14. ^{13}C NMR (101 MHz, DMSO-d_6, 298 K) of compound **S9**

7.1.8 Spectra of S11

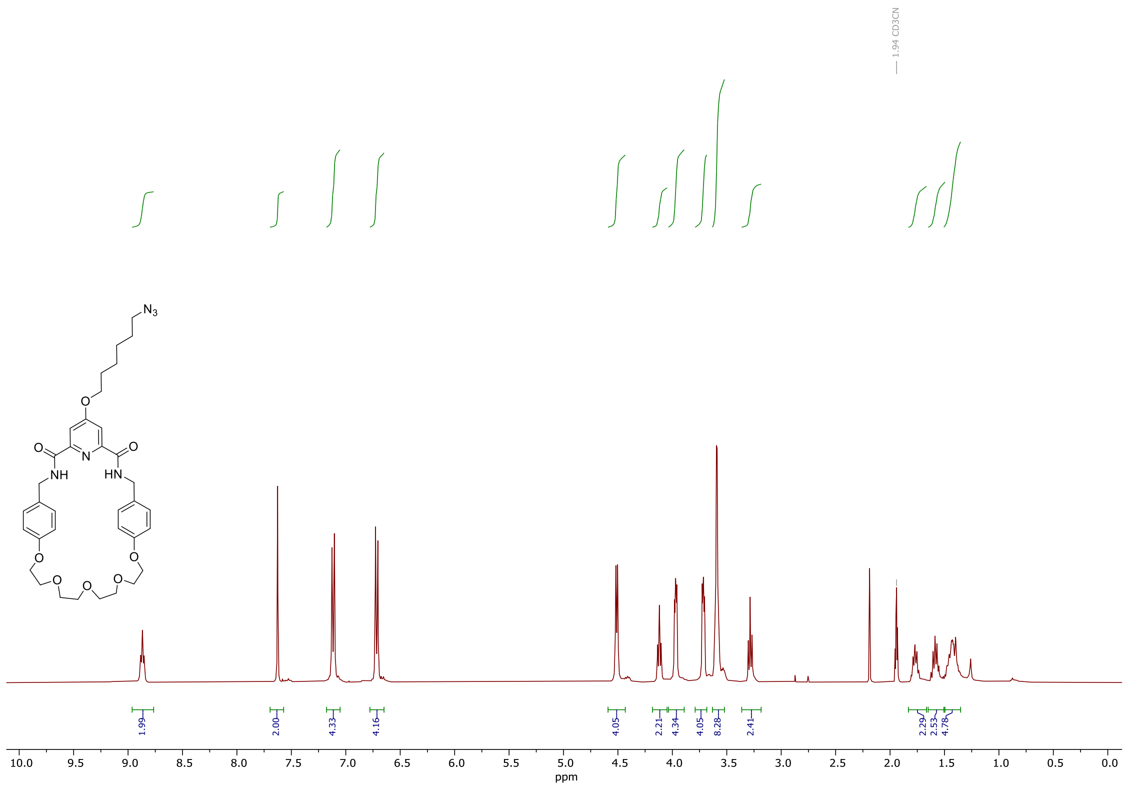

Spectrum S15. ^{1}H NMR (400 MHz, MeCN-d_3, 298 K) spectrum of compound **S11**

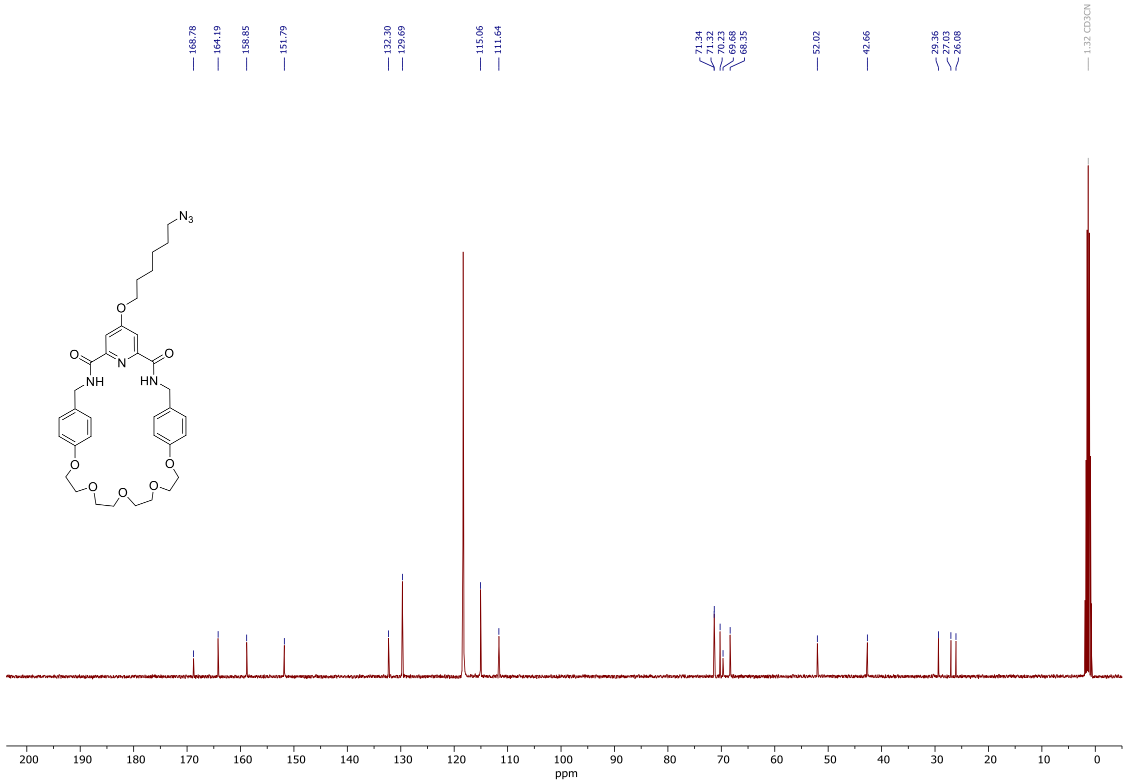

Spectrum S16. ^{13}C NMR (101 MHz, MeCN-d_3, 298 K) spectrum of compound **S11**

7.1.9 Spectra of S13

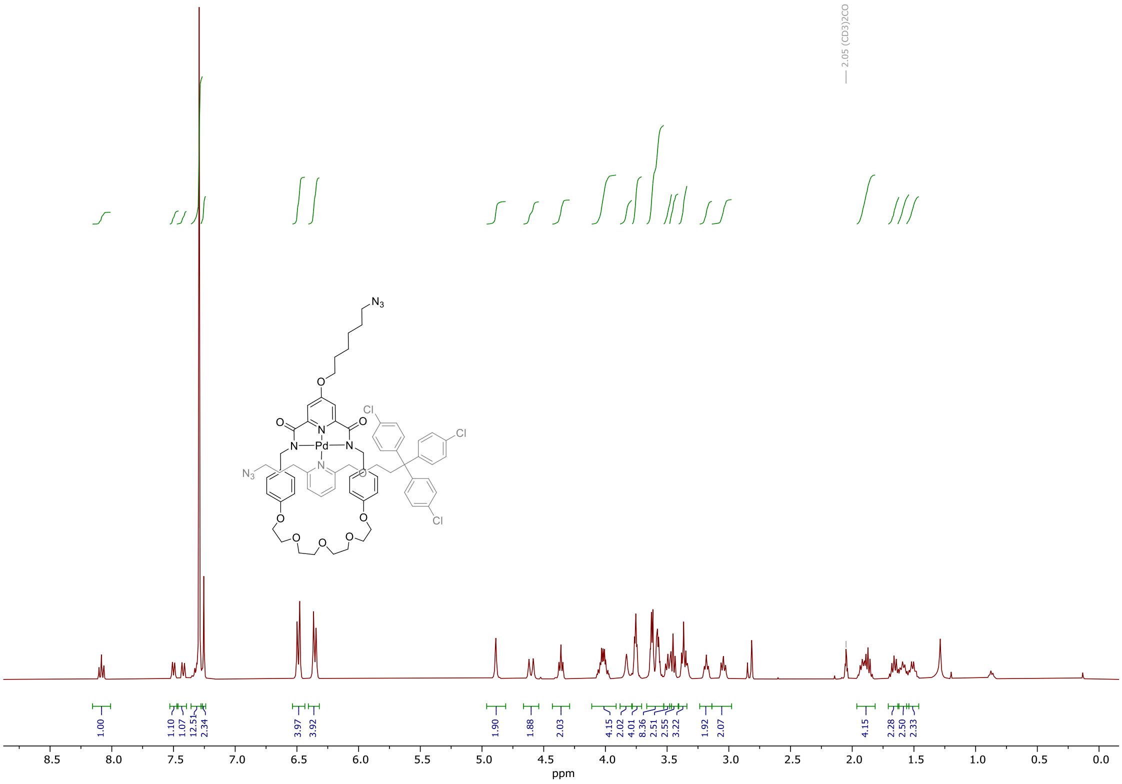

Spectrum S17. ^{1}H NMR (400 MHz, acetone-d_6, 298 K) spectrum of compounds **S13**

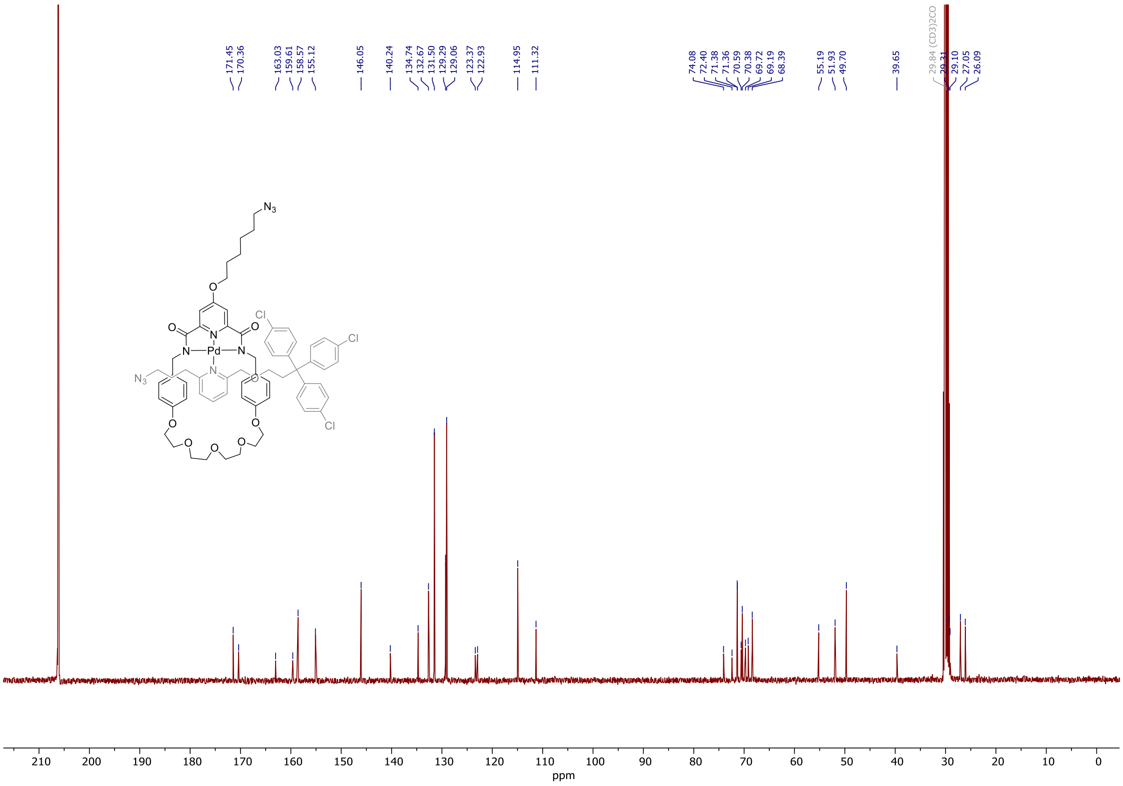

Spectrum S18. ^{13}C NMR (101 MHz, acetone-d_6, 298 K) spectrum of compound **S13**

7.1.10 Spectra of S14

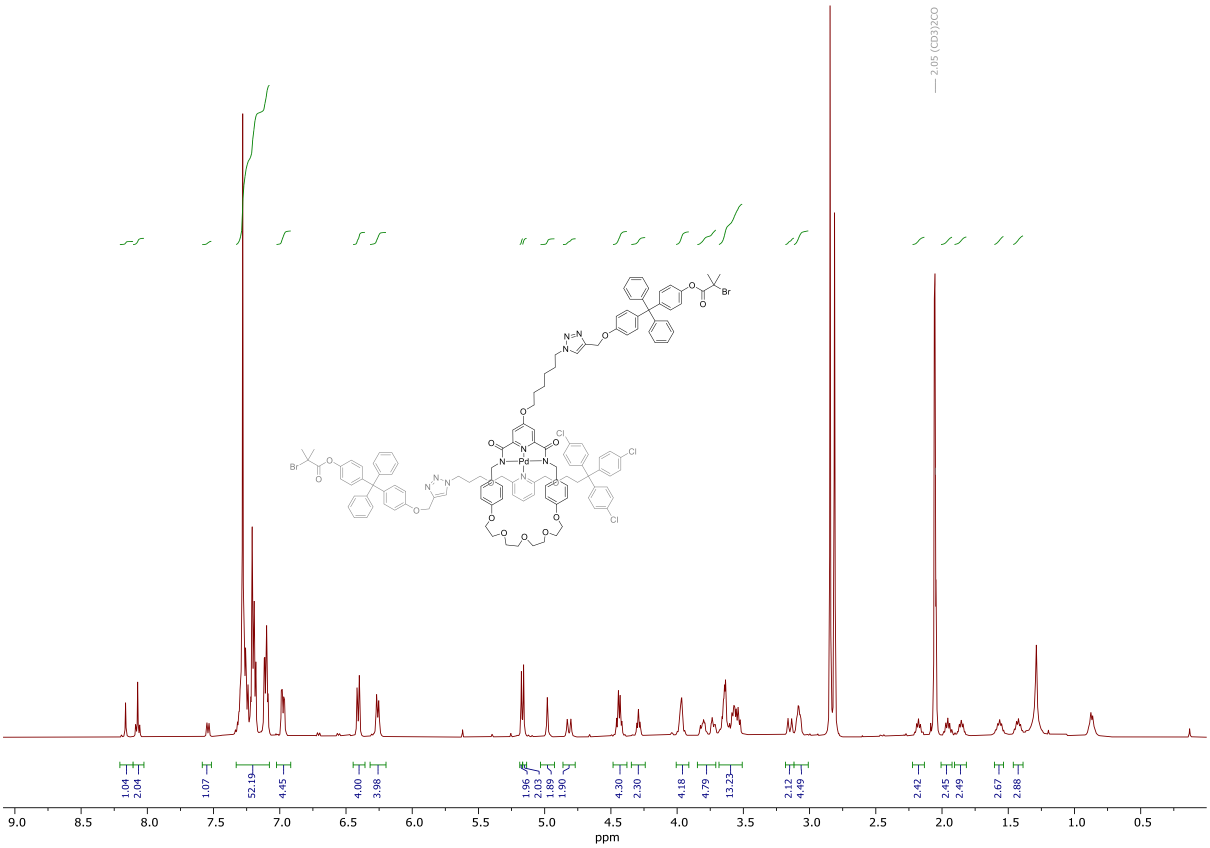

Spectrum S19. ^{1}H NMR (500 MHz, Acetone-d$_6$, 298 K) spectrum of compound **S14**

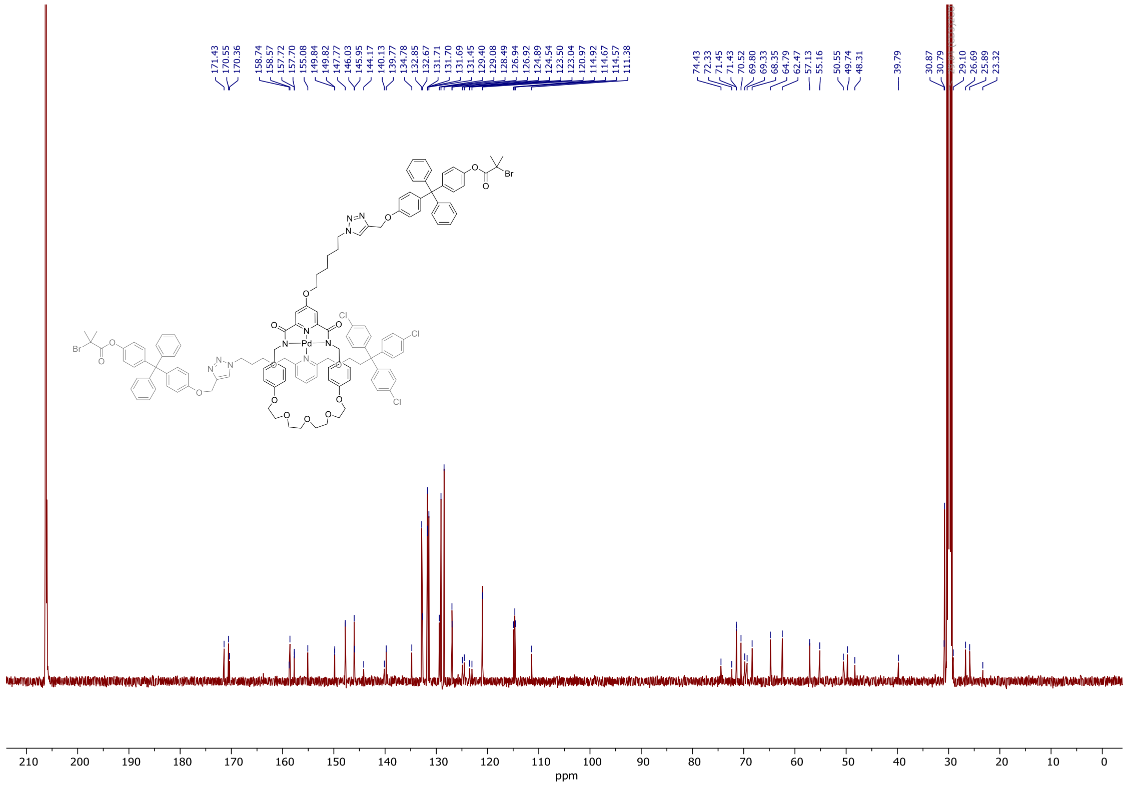

Spectrum S20. ^{13}C NMR (126 MHz, Acetone-d$_6$, 298 K) spectrum of compound **S14**

7.1.11 Synthesis of S15

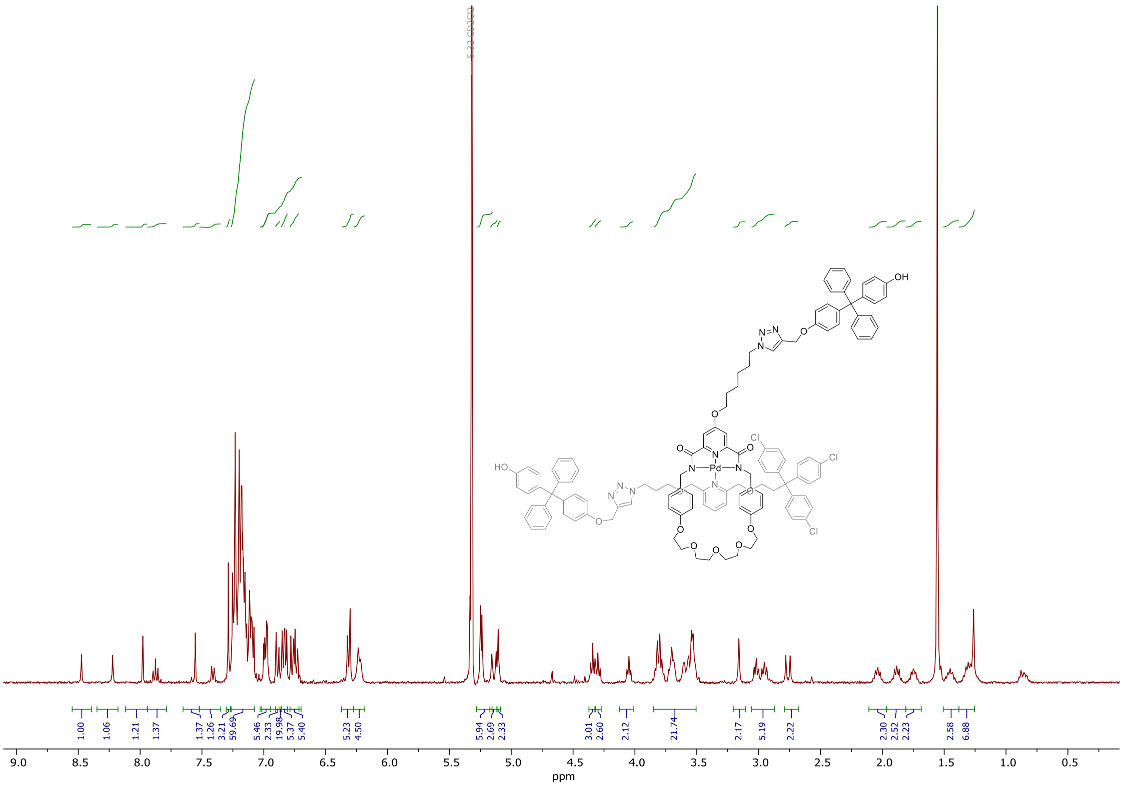

Spectrum S21. ^{1}H NMR (400 MHz, DCM-d_2, 298 K) of compound **S15**

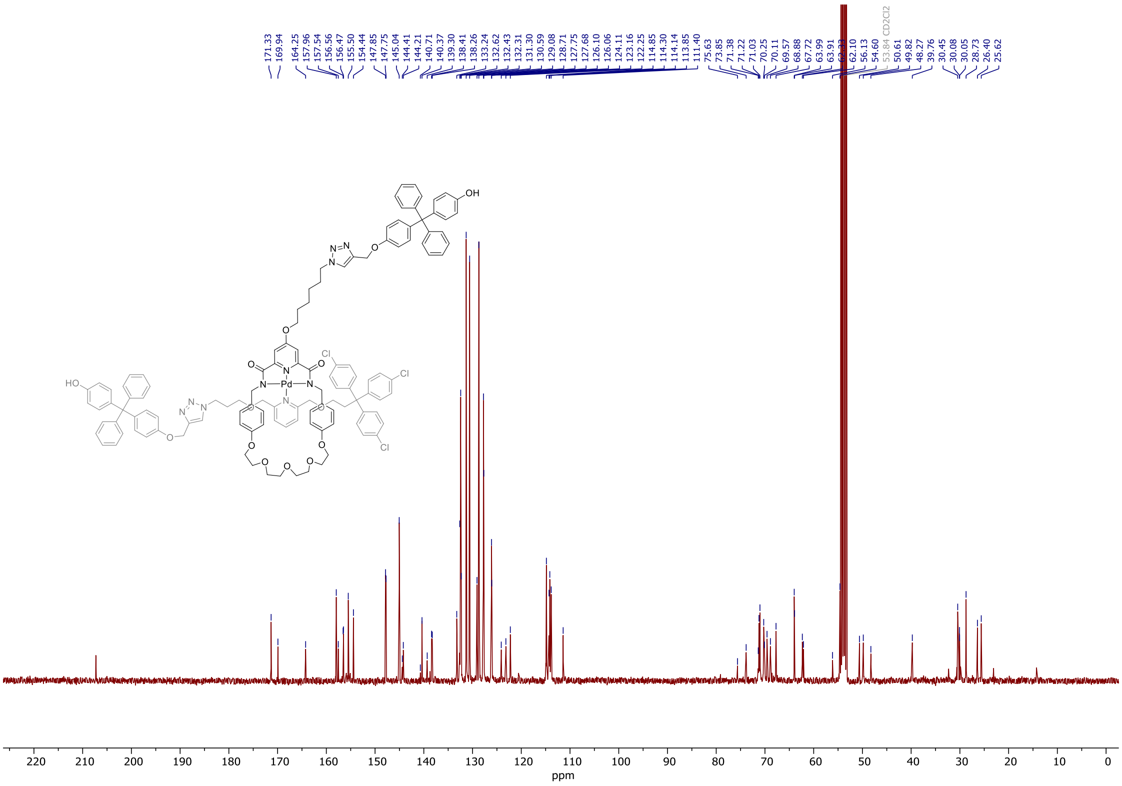

Spectrum S22. ^{13}C NMR (101 MHz, DCM-d_2, 298 K) of compound **S15**

7.1.12 Spectra of S16

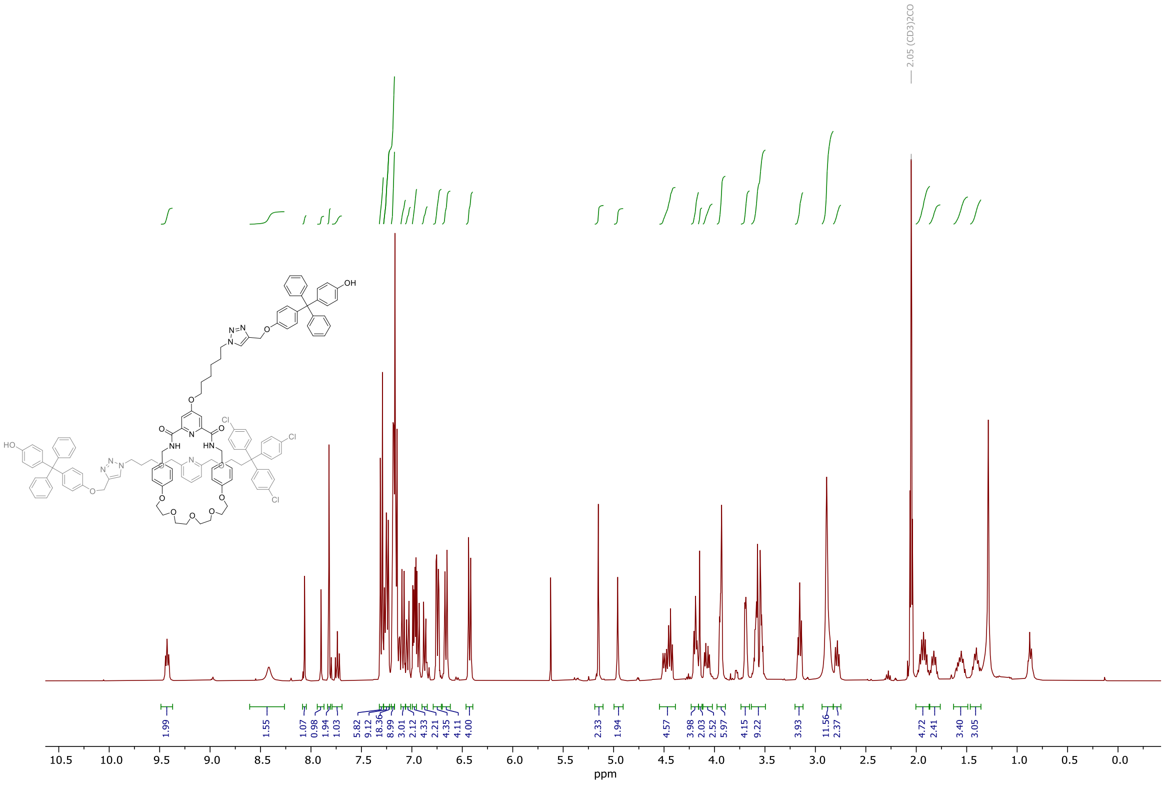

Spectrum S23. ^{1}H NMR (400 MHz, acetone-d_6, 298 K) of compound **S16**

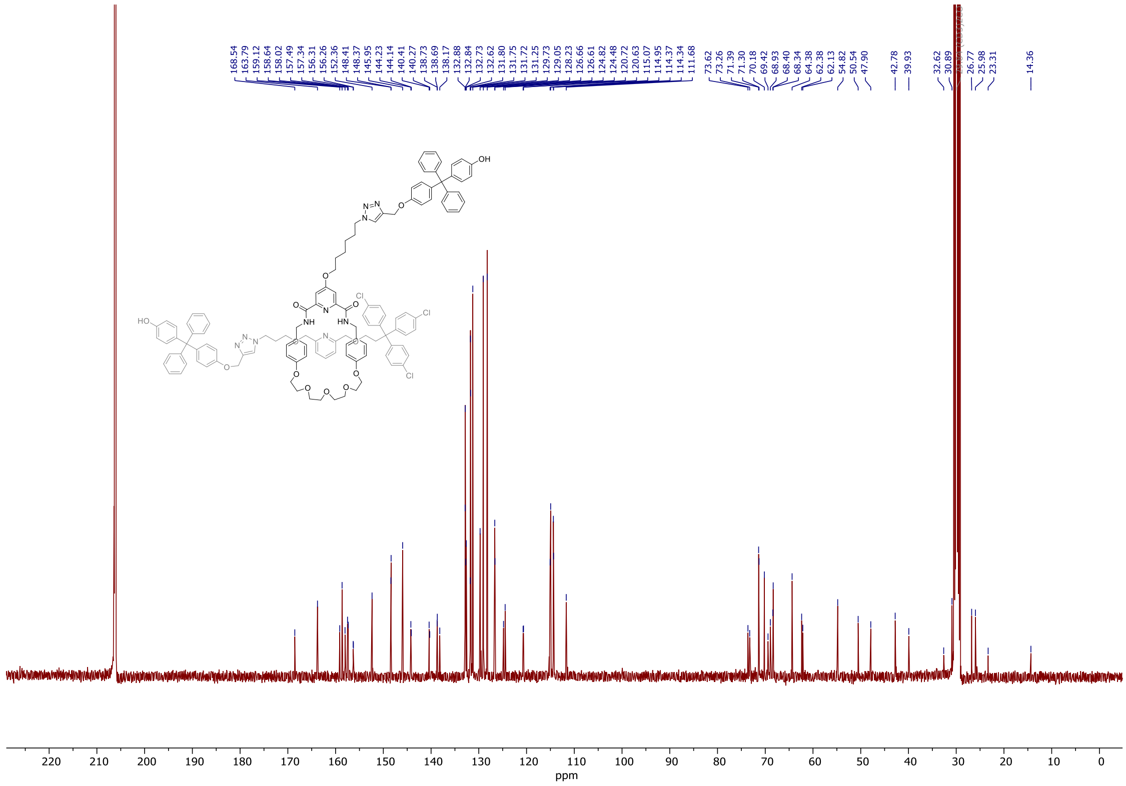

Spectrum S24. ^{13}C NMR (101 MHz, acetone-d_6, 298 K) of compound **S16**

7.1.13 Spectra of S17

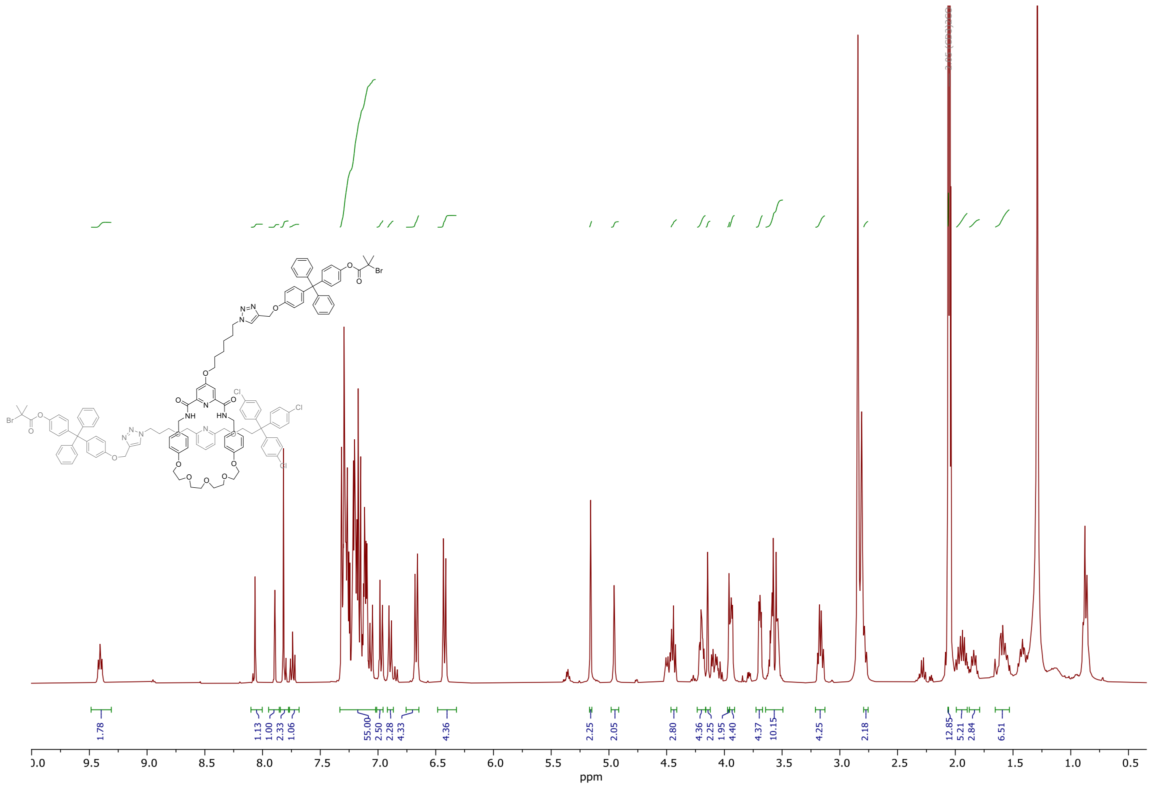

Spectrum S25. ^{1}H NMR (400 MHz, acetone-d$_6$, 298 K) of compound **S17.** Peak at 5.3 ppm is due to external impurity from syringe (see Figure S2).

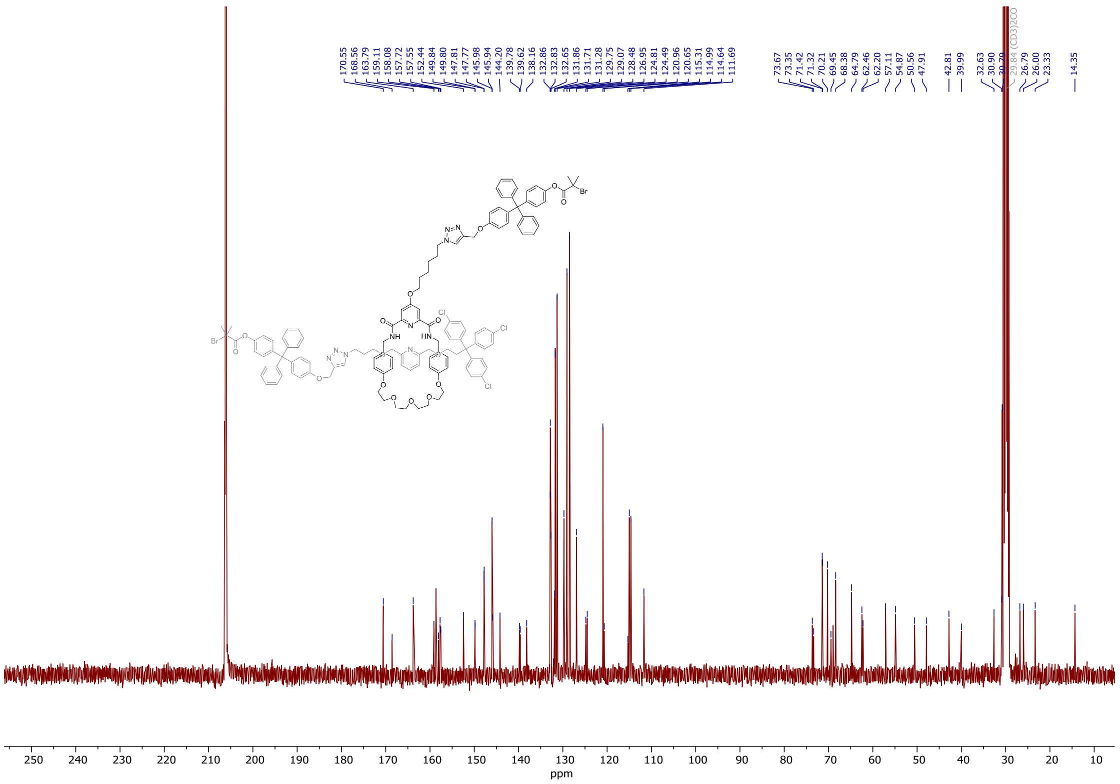

Spectrum S26. ^{13}C NMR (101 MHz, acetone-d$_6$, 298 K) of compound **S17**

7.1.14 Spectra of S19

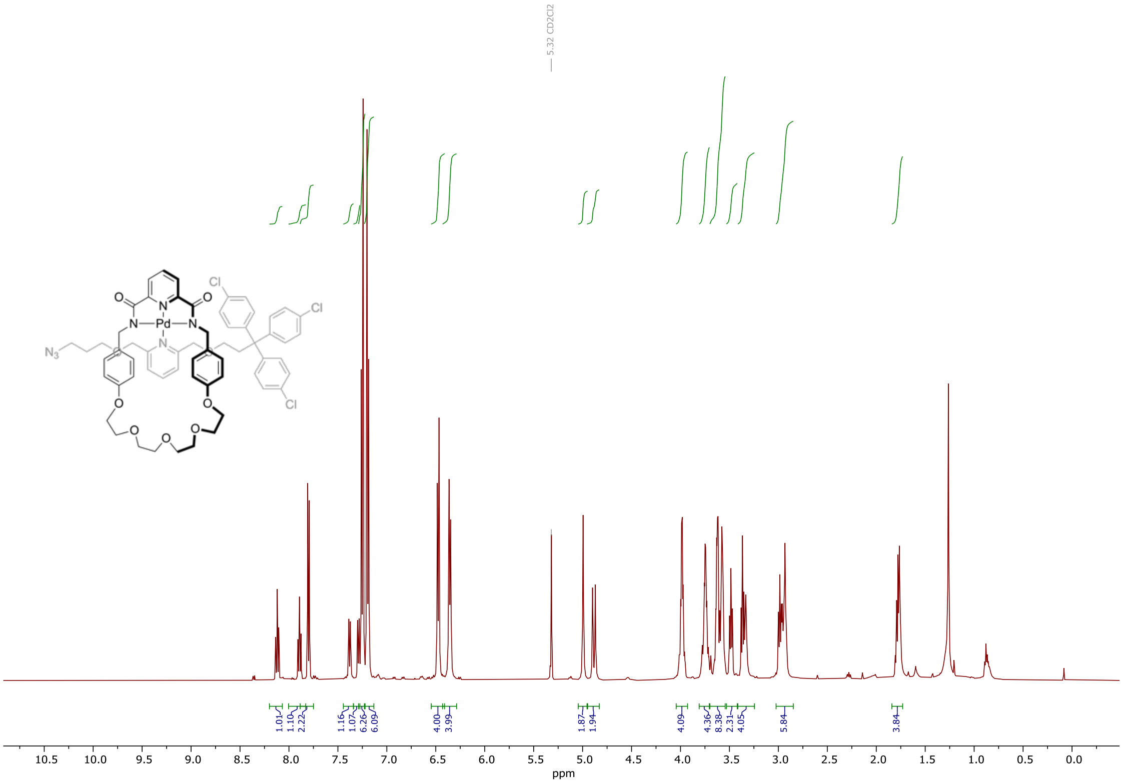

Spectrum S27. ^{1}H NMR (500 MHz, DCM-d_2, 298 K) of compound **S19**

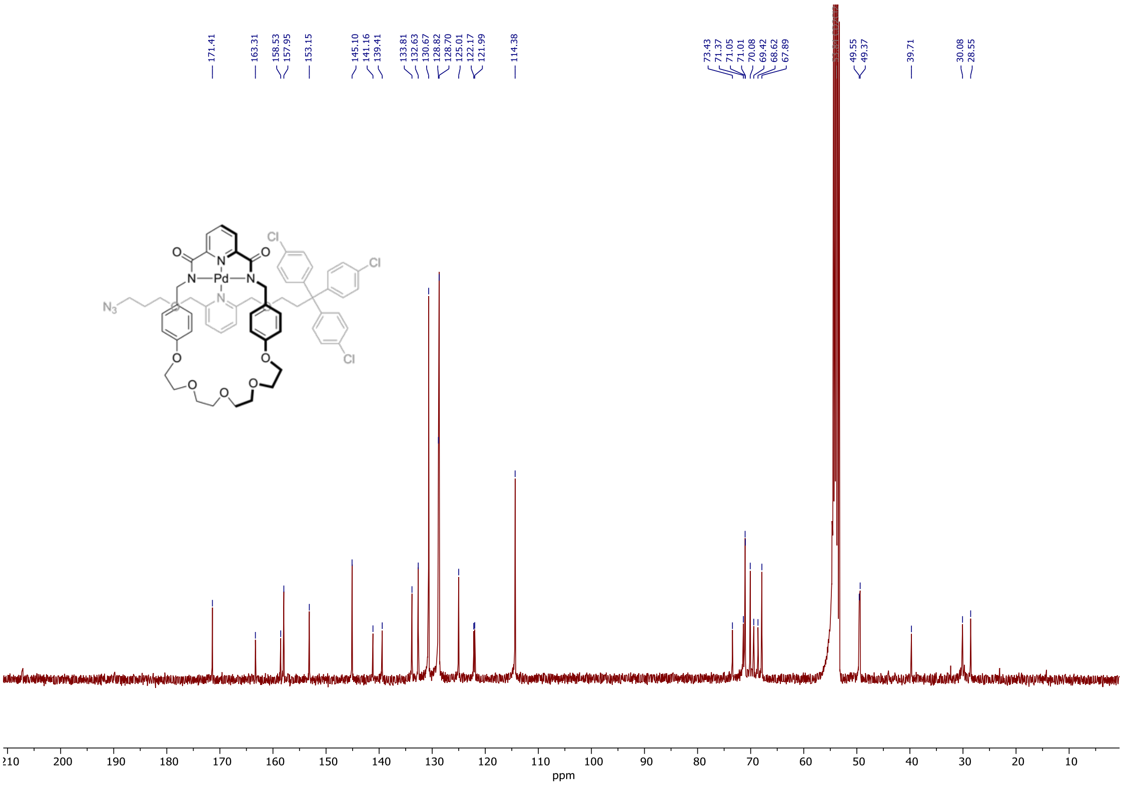

Spectrum S28. ^{13}C NMR (101 MHz, DCM-d_2, 298 K) of compound **S19**

7.1.15 Spectra of S20

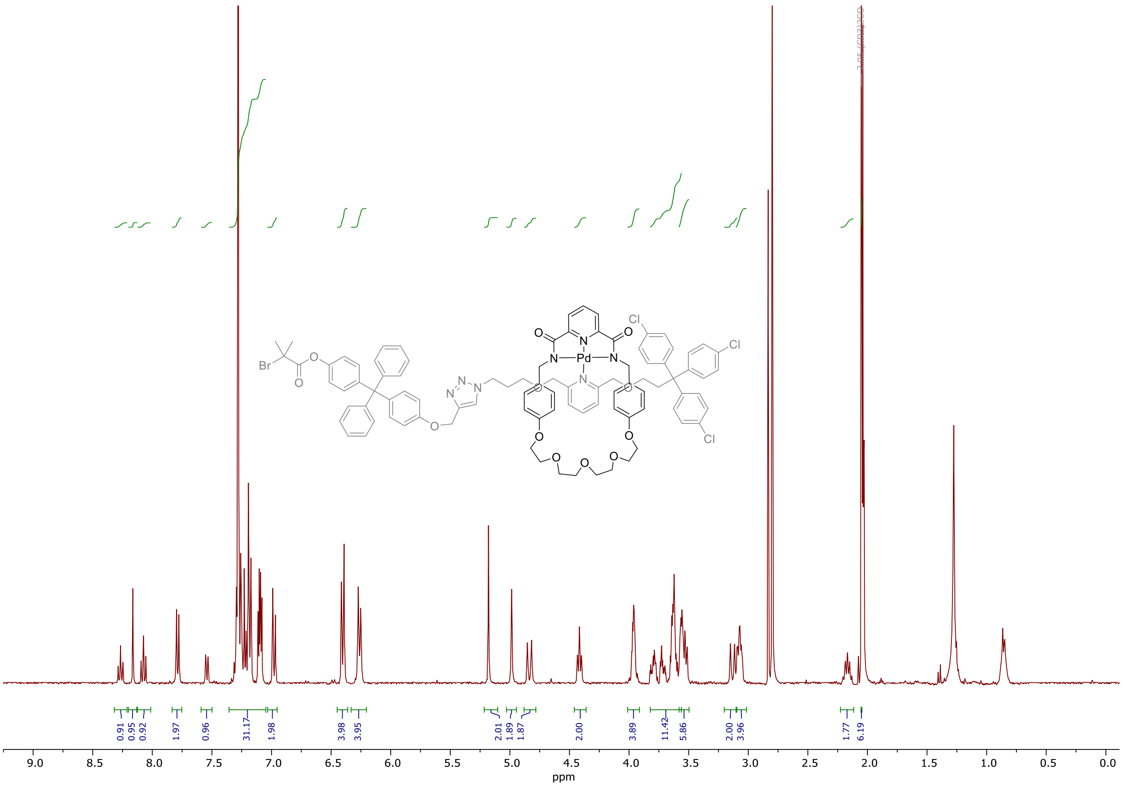

Spectrum S29. ^{1}H NMR (400 MHz, acetone-d_6, 298 K) of compound **S20**

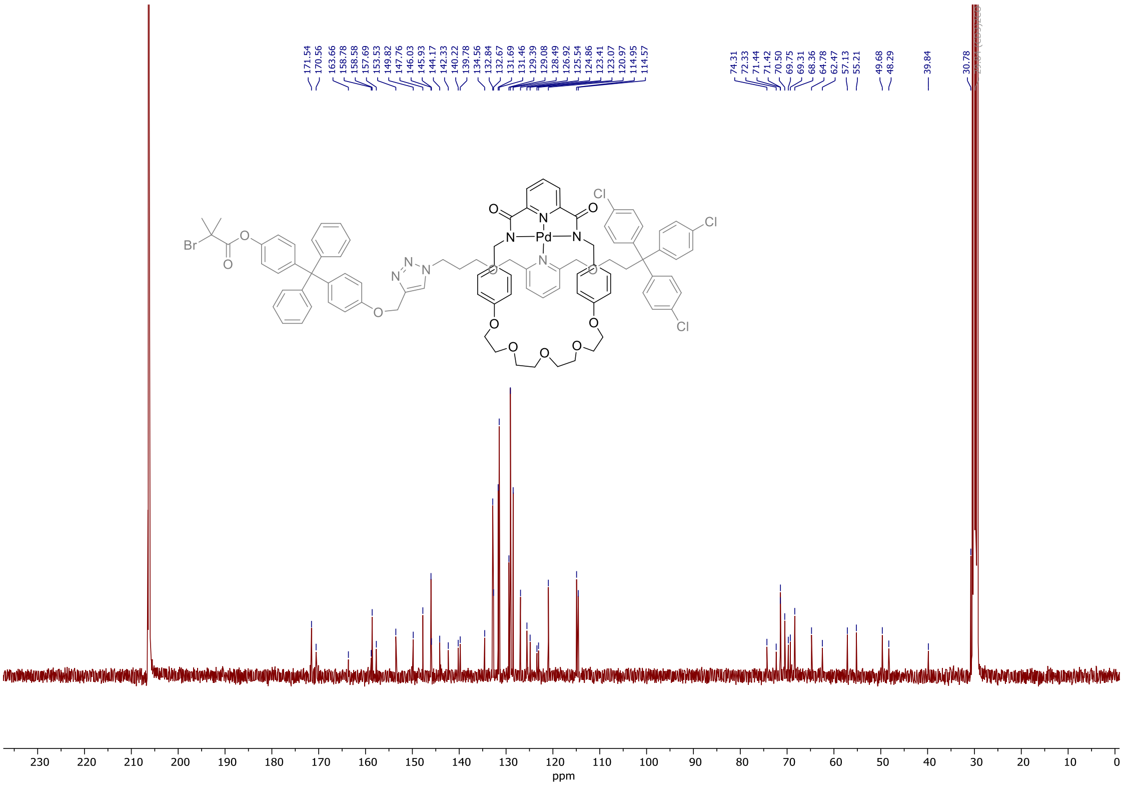

Spectrum S30. ^{13}C NMR (101 MHz, acetone-d_6, 298 K) of compound **S20**

7.1.16 Spectra of S21

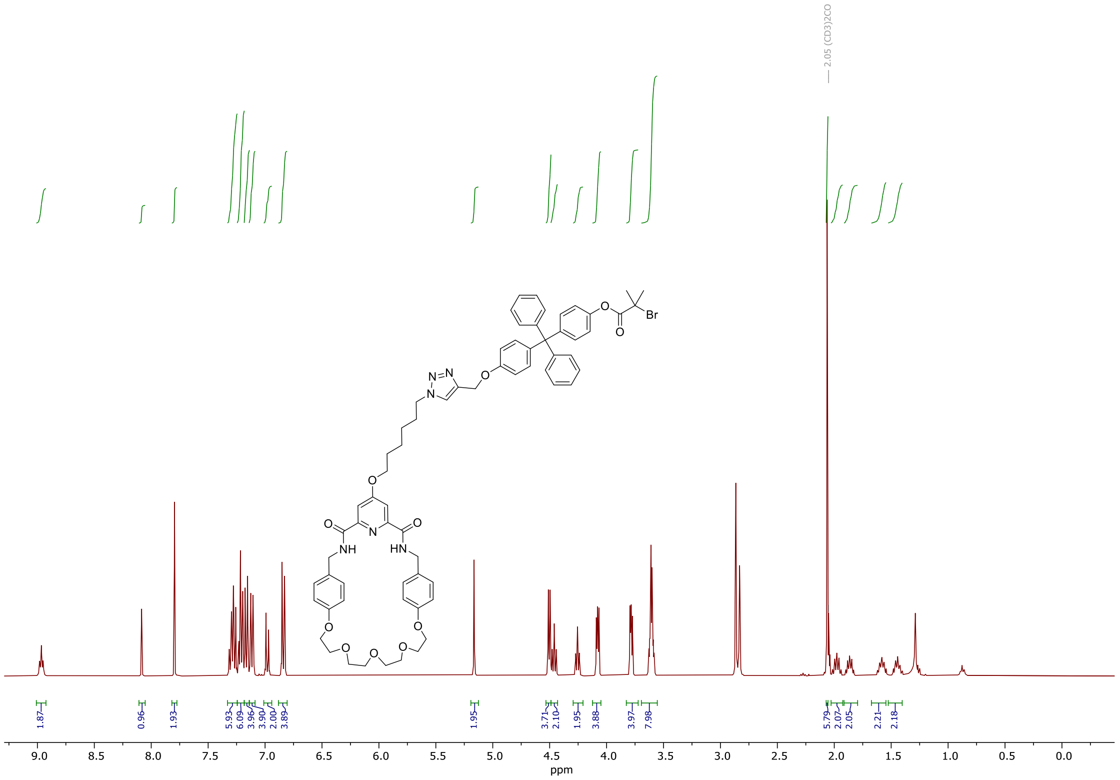

Spectrum S31. ^{1}H NMR (400 MHz, acetone-d$_6$, 298 K) of compound **S21**

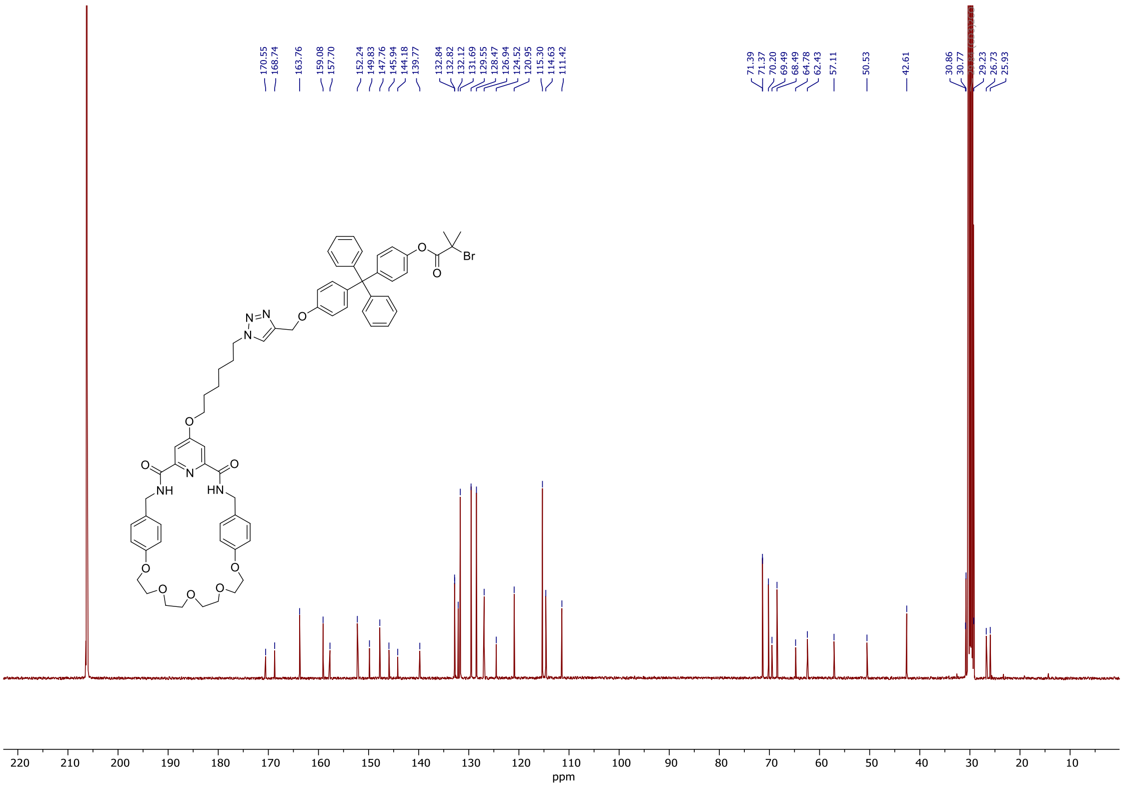

Spectrum S32. ^{13}C NMR (101 MHz, acetone-d$_6$, 298 K) of compound **S21**

7.1.17 Spectra of S22

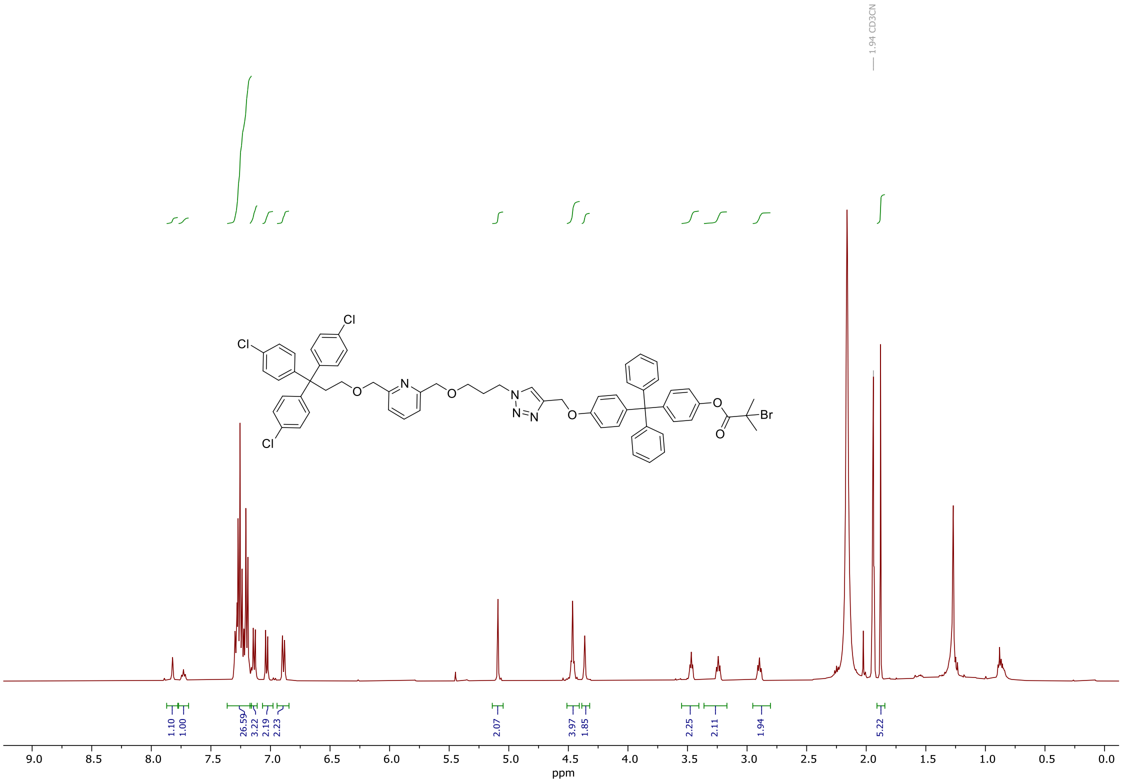

Spectrum S33. ^{1}H NMR (400 MHz, MeCN-d_3, 298 K) of compound **S22**

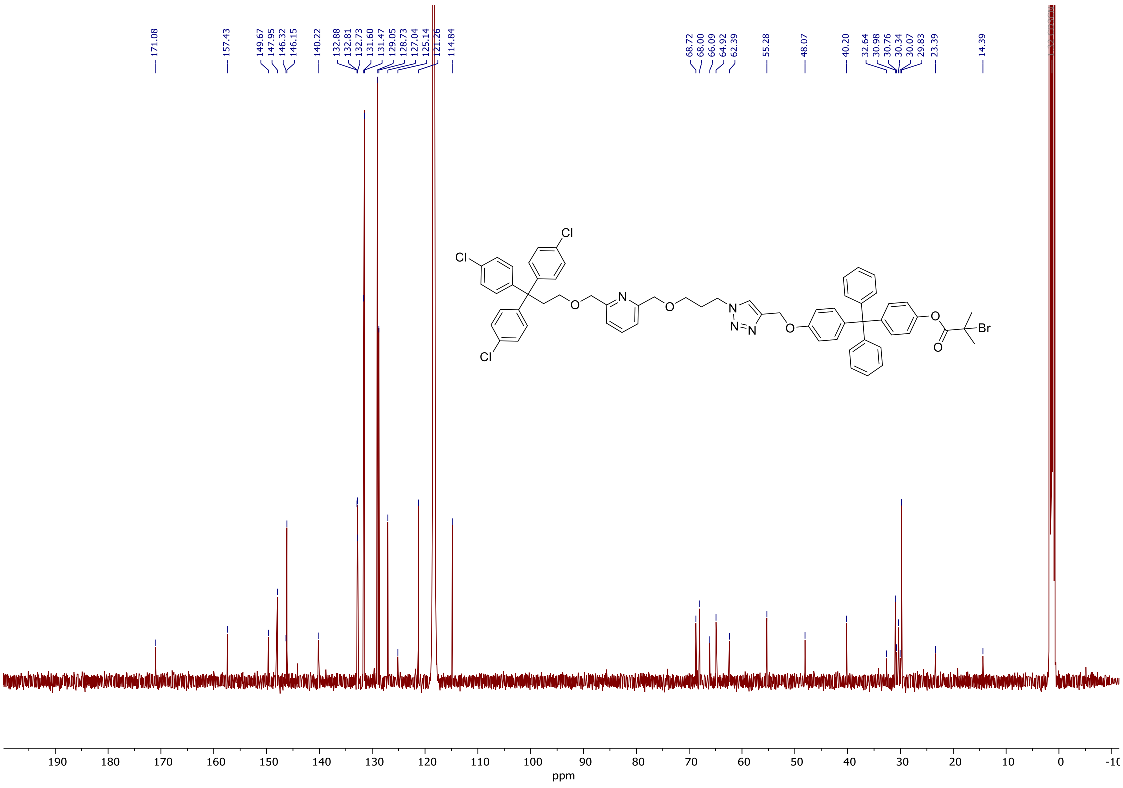

Spectrum S34. ^{13}C NMR (101 MHz, MeCN-d_3, 298 K) of compound **S22**

7.2 Polymer NMR spectra

7.2.1 Spectra of 1_{Pd}

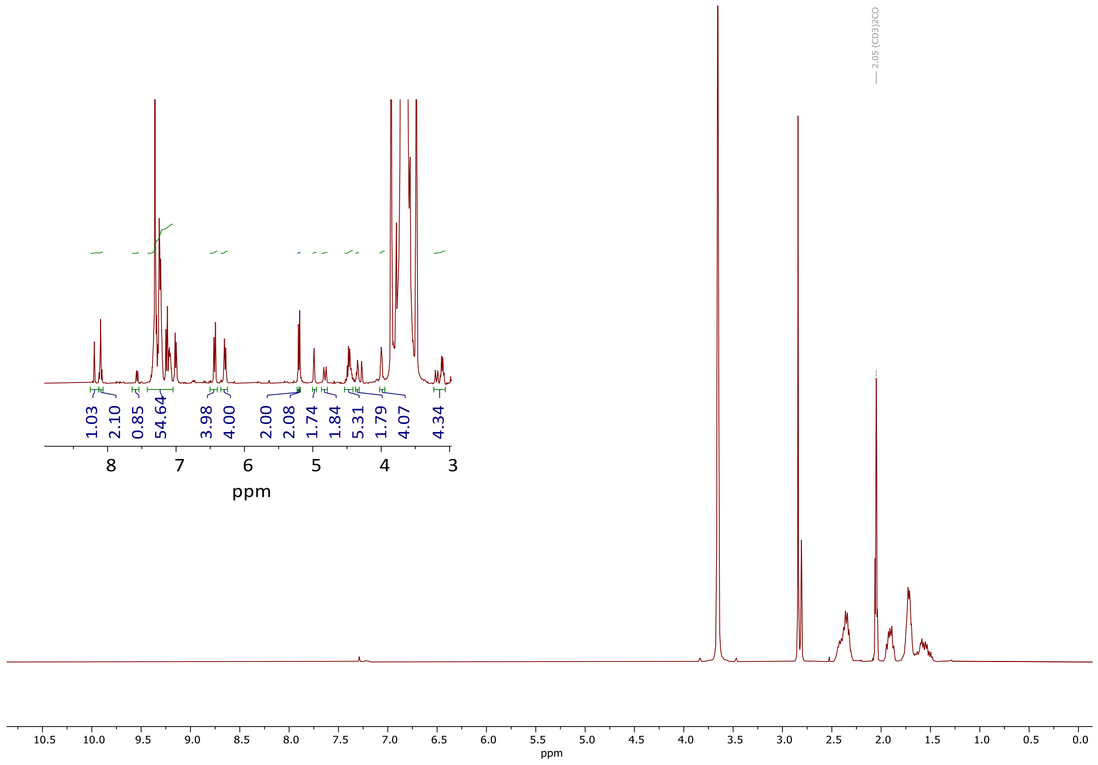

Spectrum S35. ^{1}H NMR (400 MHz, acetone-d$_6$, 298 K) of compound $\mathbf{1_{Pd}}$

7.2.2 Spectra of 1_H

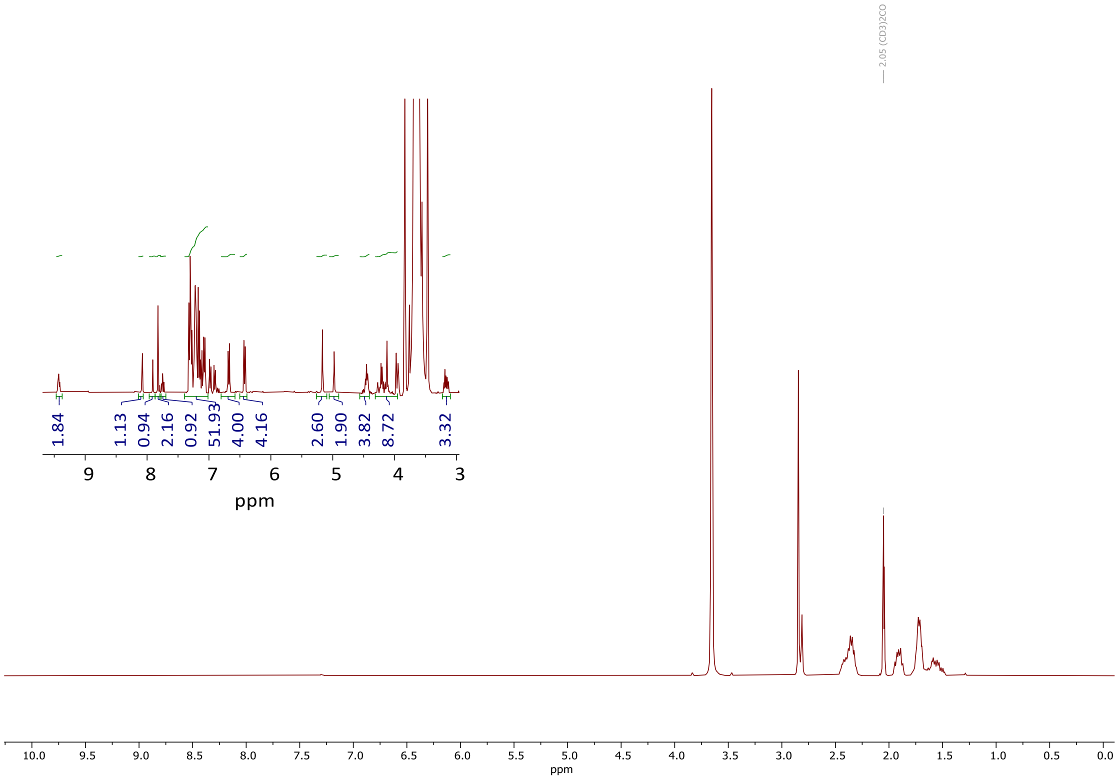

Spectrum S36. ^{1}H NMR (400 MHz, acetone-d$_6$, 298 K) of compound $\mathbf{1_H}$

7.2.3 Spectra of S23

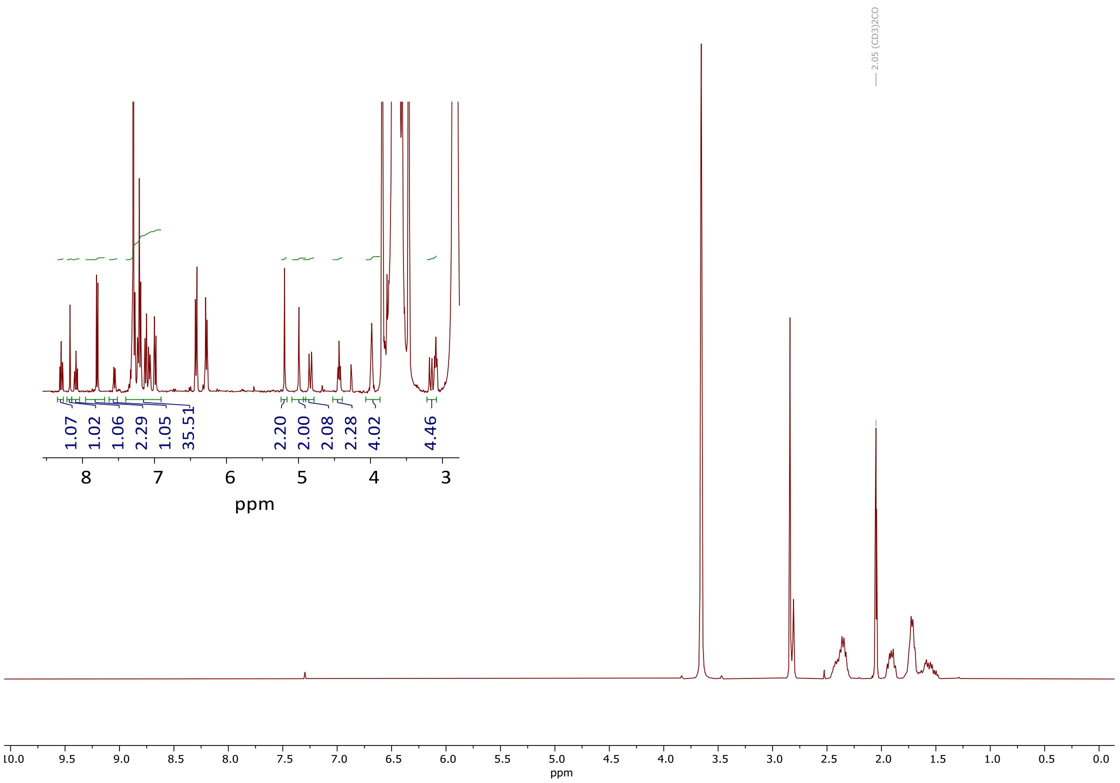

Spectrum S37. ^{1}H NMR (400 MHz, acetone-d_6, 298 K) of compound **S23**

7.2.4 Spectra of 2_H

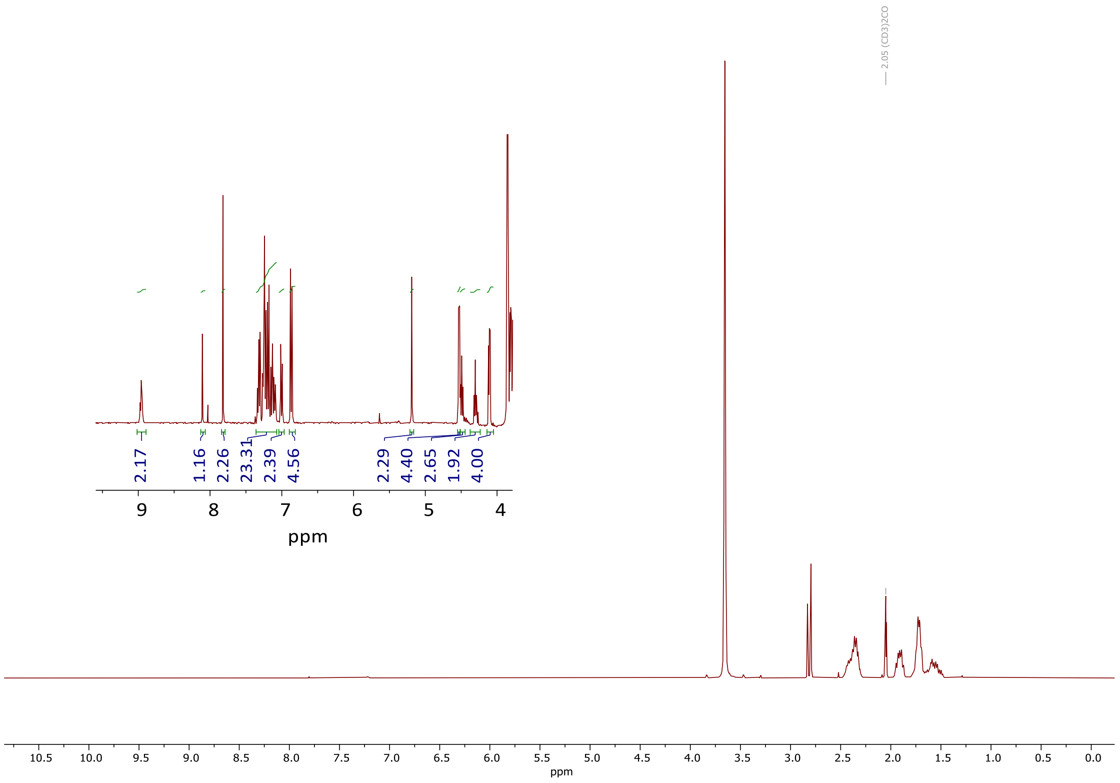

Spectrum S38. ^{1}H NMR (400 MHz, acetone-d_6, 298 K) of compound **2_H**

7.2.5 Spectra of 6

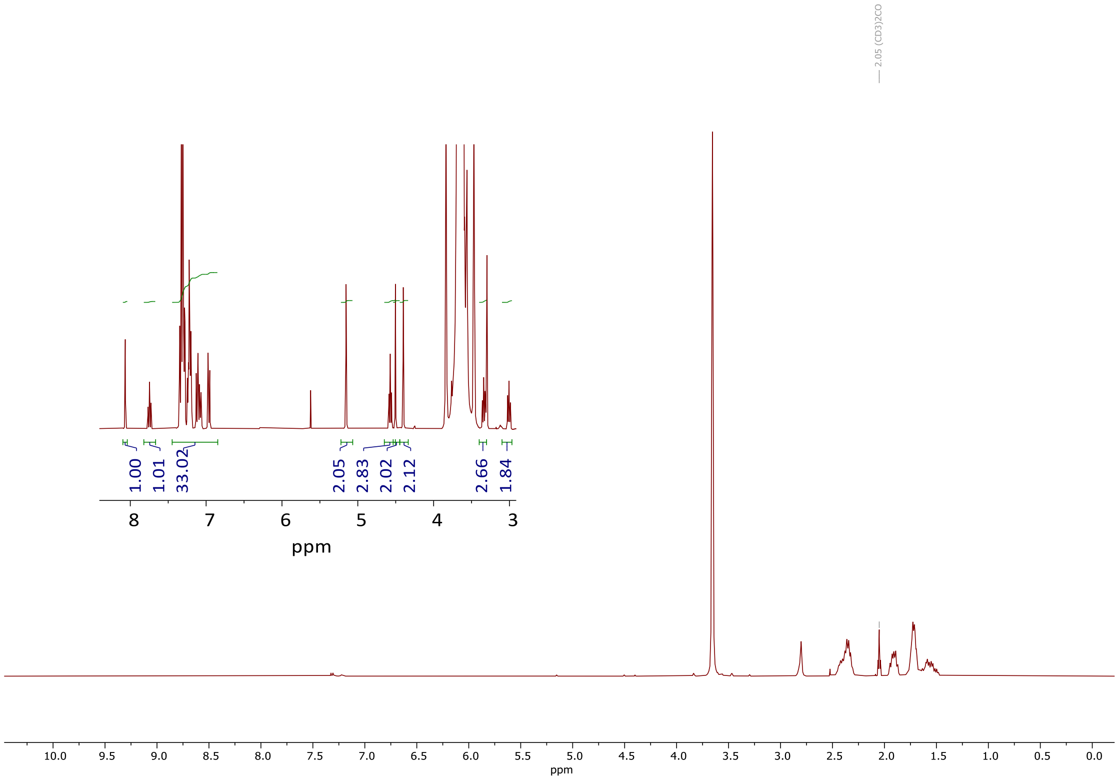

Spectrum S39. ^{1}H NMR (400 MHz, acetone-d_6, 298 K) of compound **6**

7.3 Post sonication NMR spectra

7.3.1 Post-sonication spectra of 1_{Pd} (run 1)

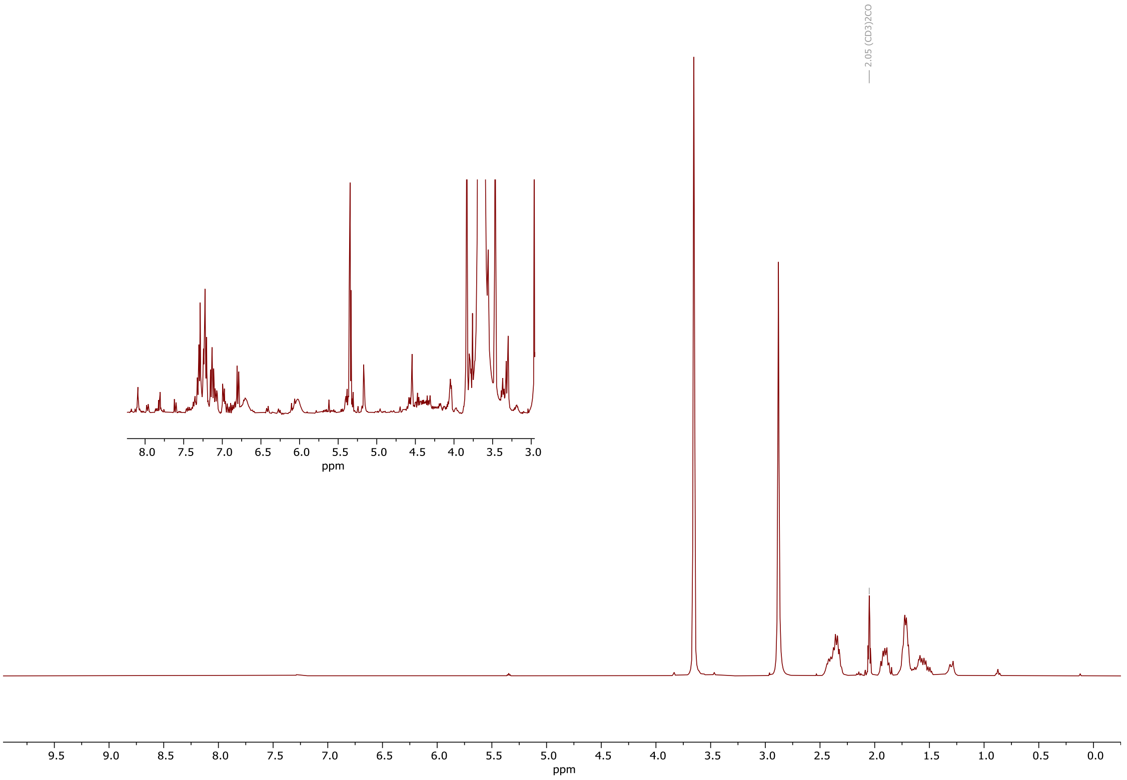

Spectrum S40. ^{1}H NMR (400 MHz, acetone-d_6, 298 K) of $\mathbf{1_{Pd}}$ post-sonication before methanol washing

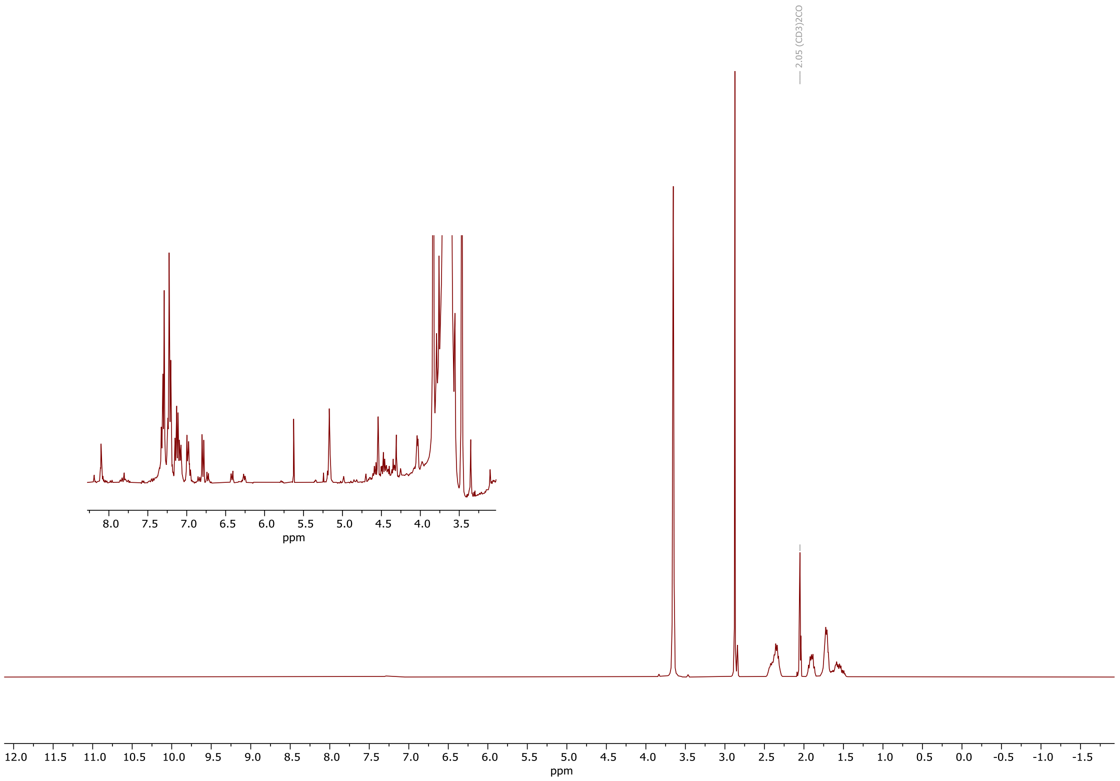

Spectrum S41. ^{1}H NMR (400 MHz, acetone-d_6, 298 K) of $\mathbf{1_{Pd}}$ post-sonication after methanol washing

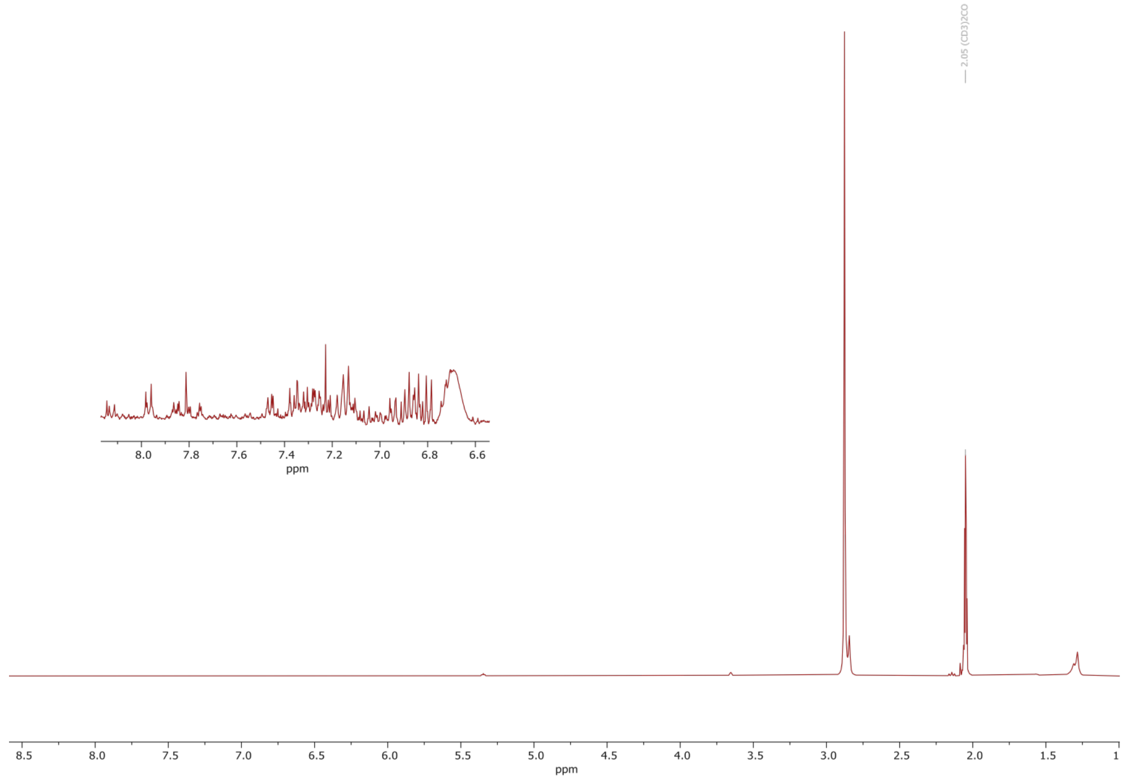

Spectrum S42. ^{1}H NMR (400 MHz, acetone-d_6, 298 K) of concentrated methanol washings.

7.3.1 Post-sonication spectra of 1_{Pd} (run 2)

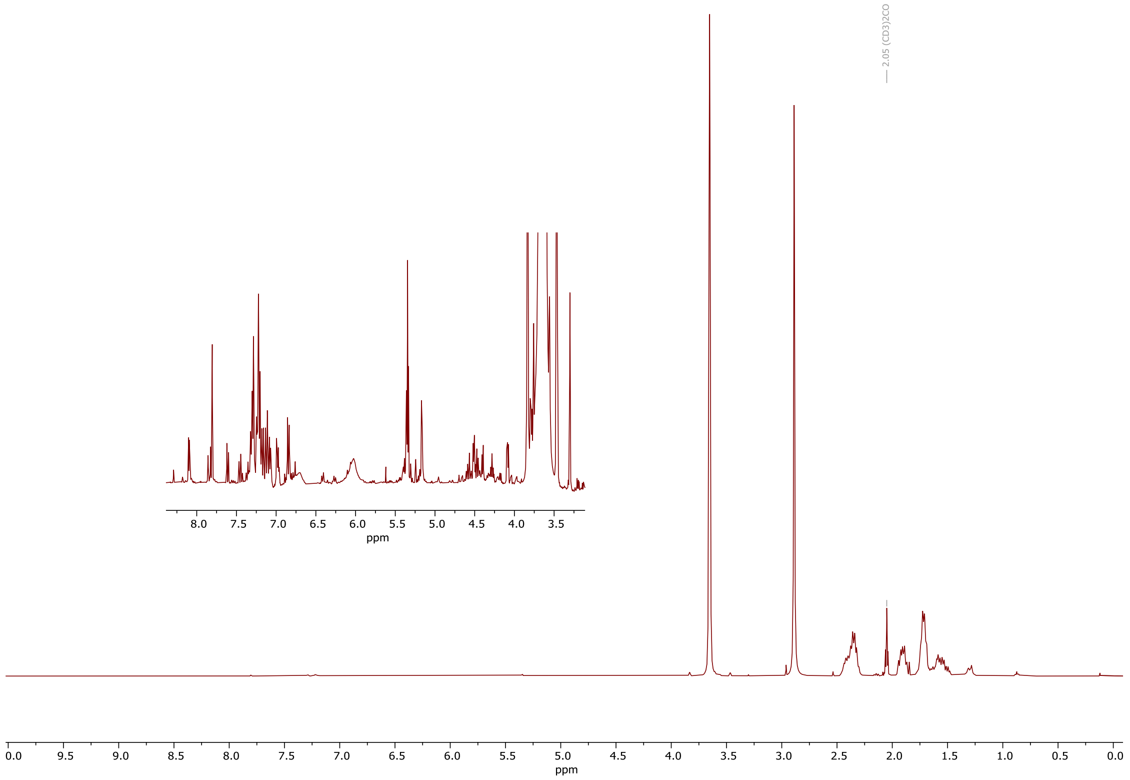

Spectrum S43. ^{1}H NMR (400 MHz, acetone-d_6, 298 K) of $\mathbf{1_{Pd}}$ post-sonication before methanol washing

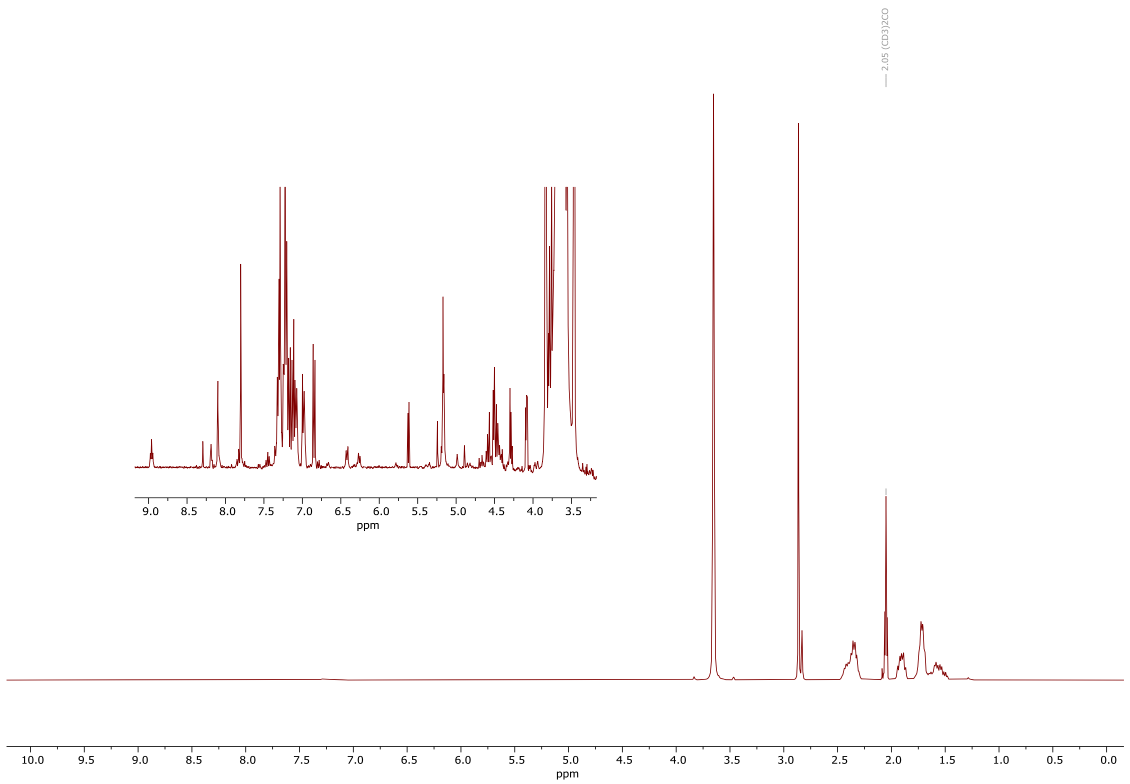

Spectrum S44. ^{1}H NMR (400 MHz, acetone-d_6, 298 K) of $\mathbf{1_{Pd}}$ post-sonication after methanol washing

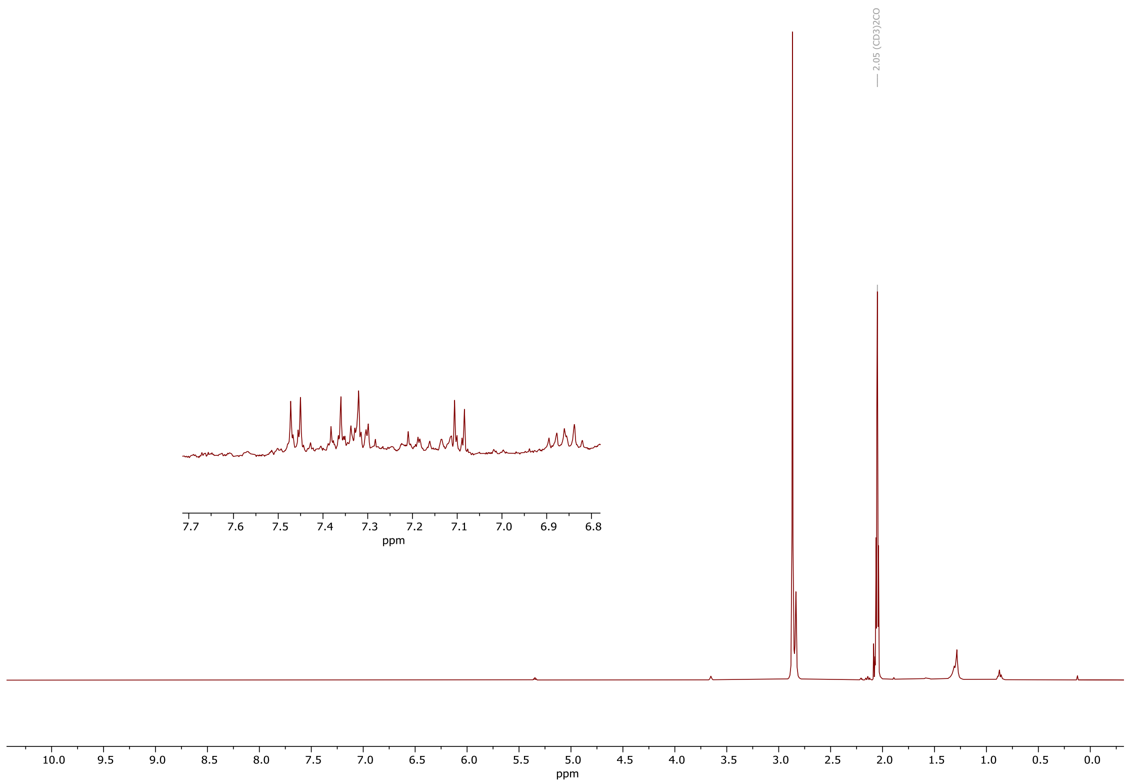

Spectrum S45. ^{1}H NMR (400 MHz, acetone-d_6, 298 K) of $\mathbf{1_{Pd}}$ post-sonication concentrated methanol washings

7.3.2 Post-sonication spectra of 1_{Pd} (run 3)

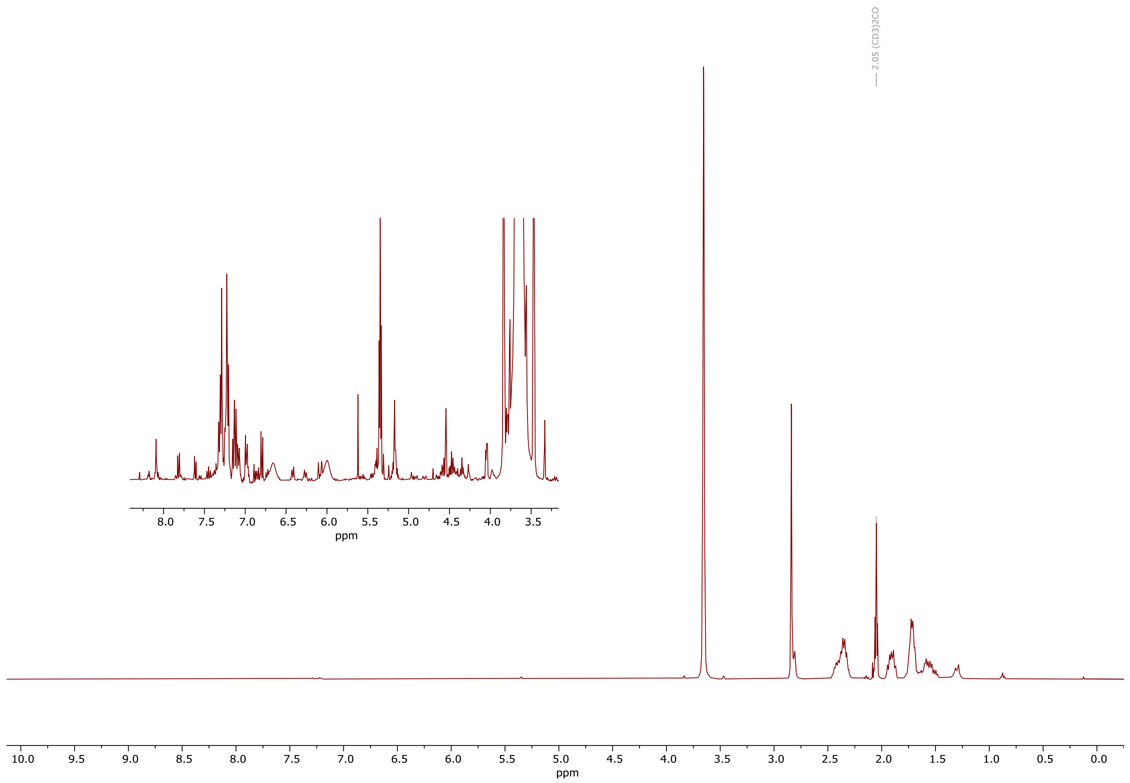

Spectrum S46. ^{1}H NMR (400 MHz, acetone-d$_6$, 298 K) of $\mathbf{1_{Pd}}$ post-sonication before methanol washing

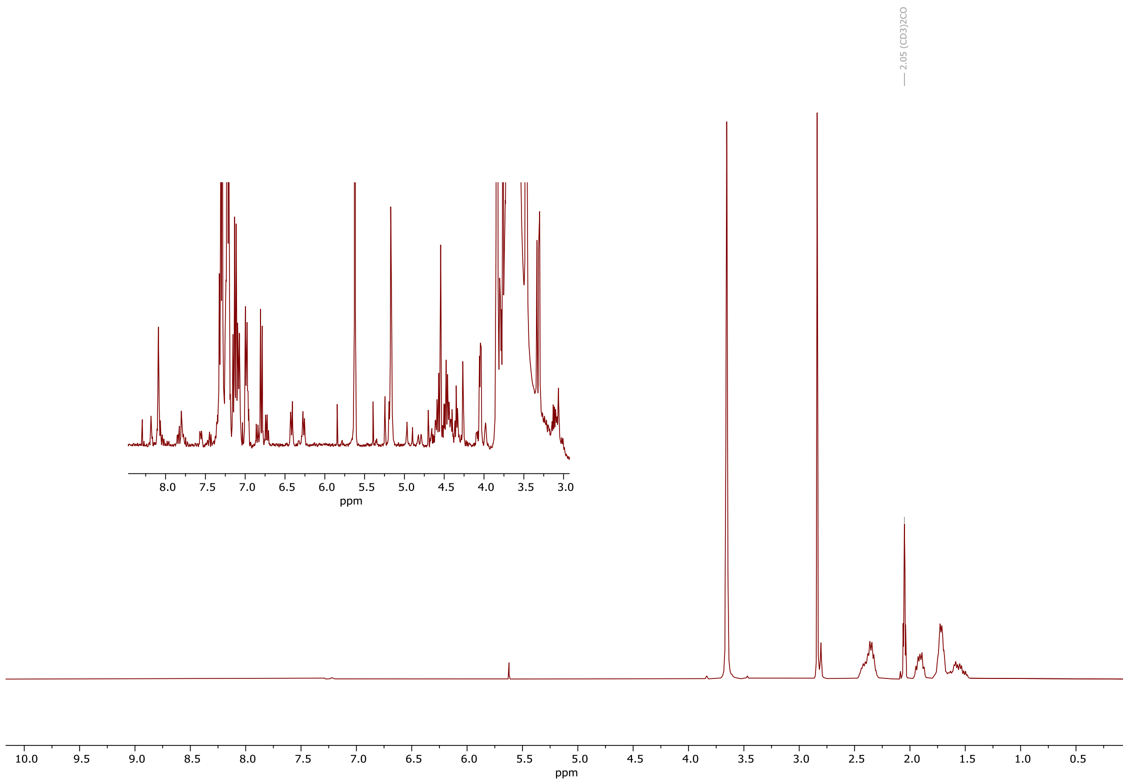

Spectrum S47. ^{1}H NMR (400 MHz, acetone-d$_6$, 298 K) of $\mathbf{1_{Pd}}$ post-sonication after methanol washing

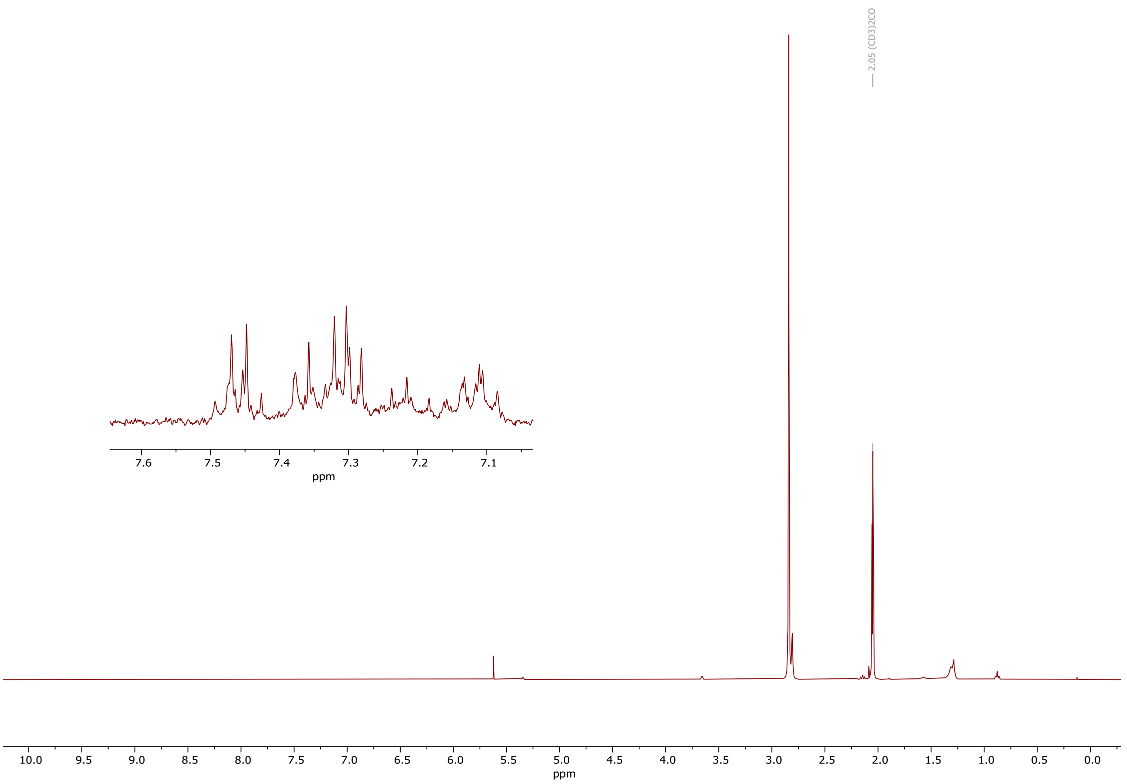

Spectrum S48. ^{1}H NMR (400 MHz, acetone-d_6, 298 K) of $\mathbf{1_{Pd}}$ post-sonication concentrated methanol washings

7.3.3 Post-sonication spectra of 1_H (run 1)

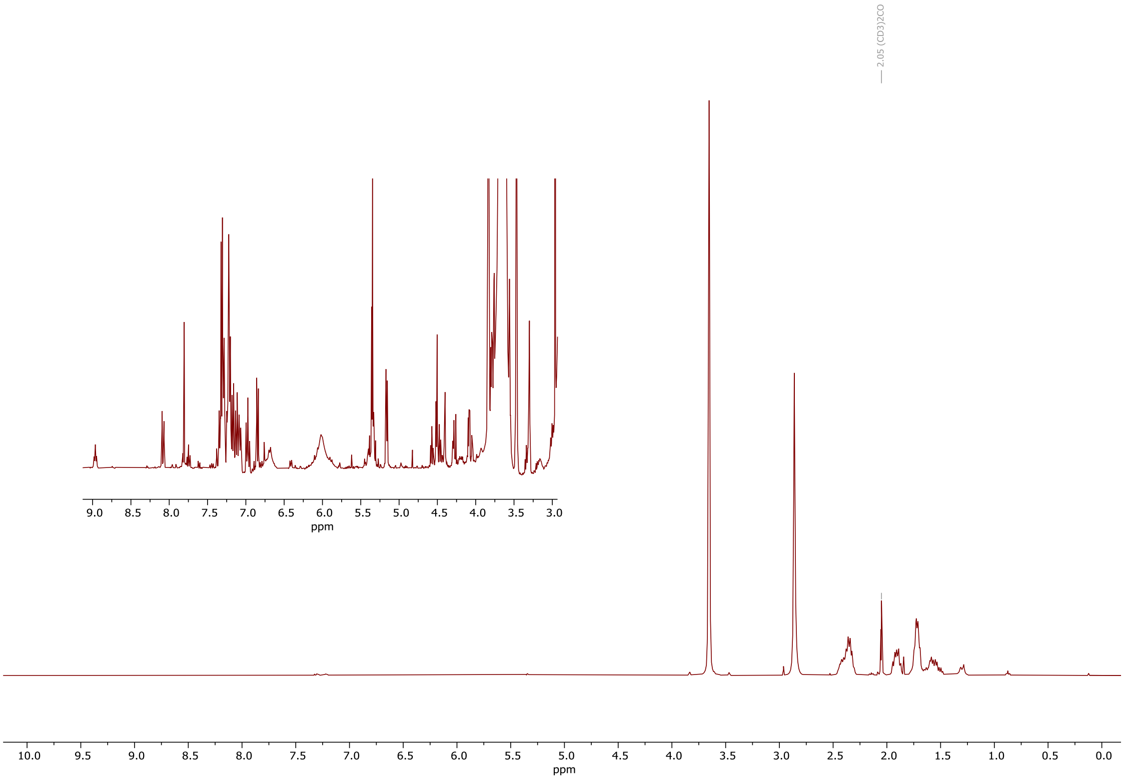

Spectrum S49. ^{1}H NMR (400 MHz, acetone-d_6, 298 K) of $\mathbf{1_H}$ post-sonication before methanol washing

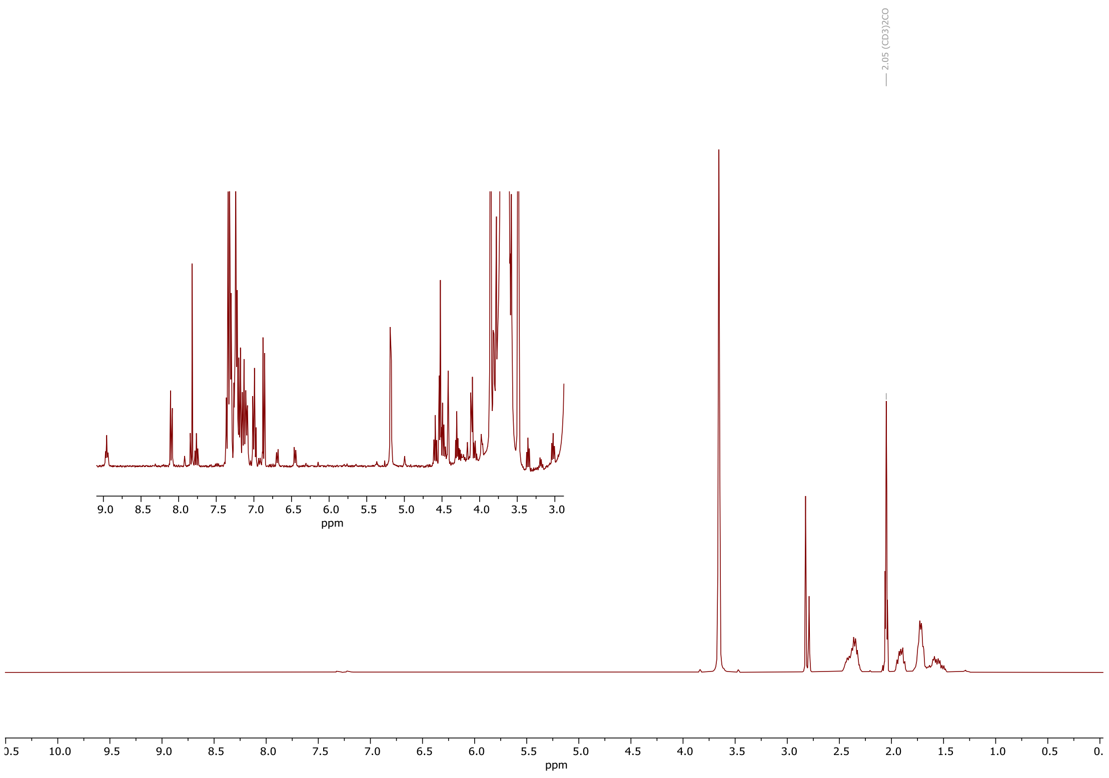

Spectrum S50. ^{1}H NMR (400 MHz, acetone-d$_6$, 298 K) of **1$_H$** post-sonication after methanol washing

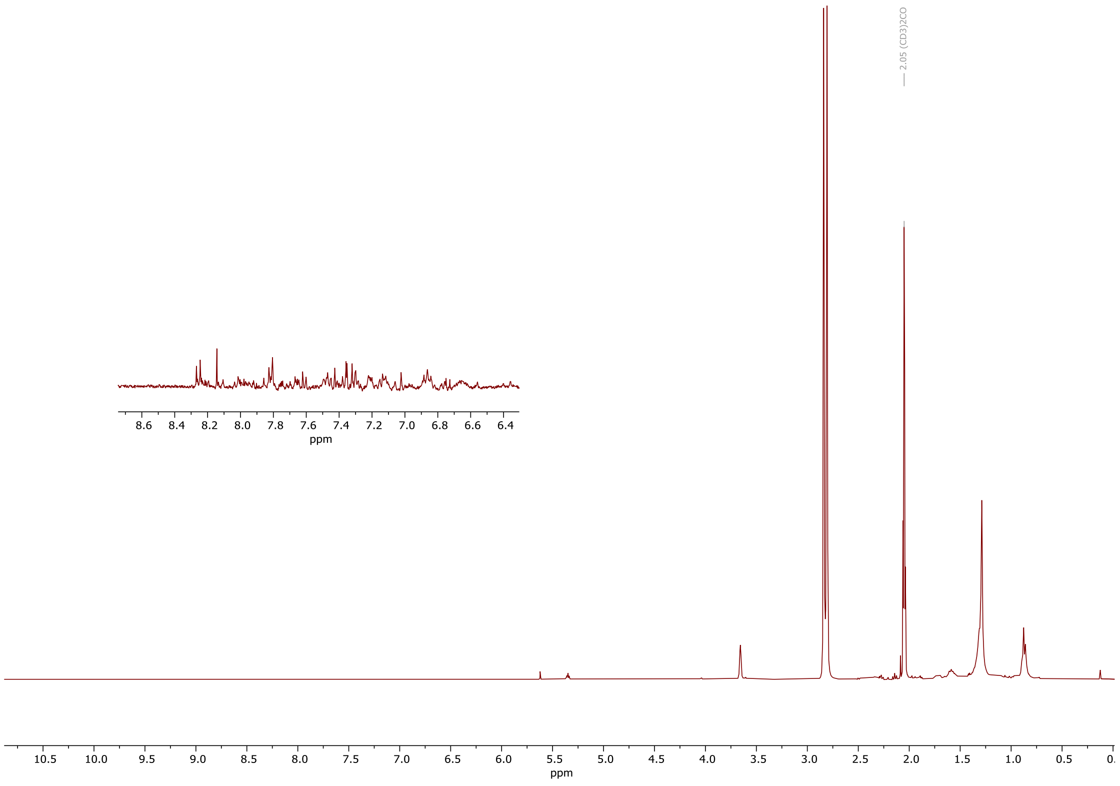

Spectrum S51. ^{1}H NMR (400 MHz, acetone-d$_6$, 298 K) of concentrated methanol washings

7.3.1 Post-sonication spectra of 1_H (run 2)

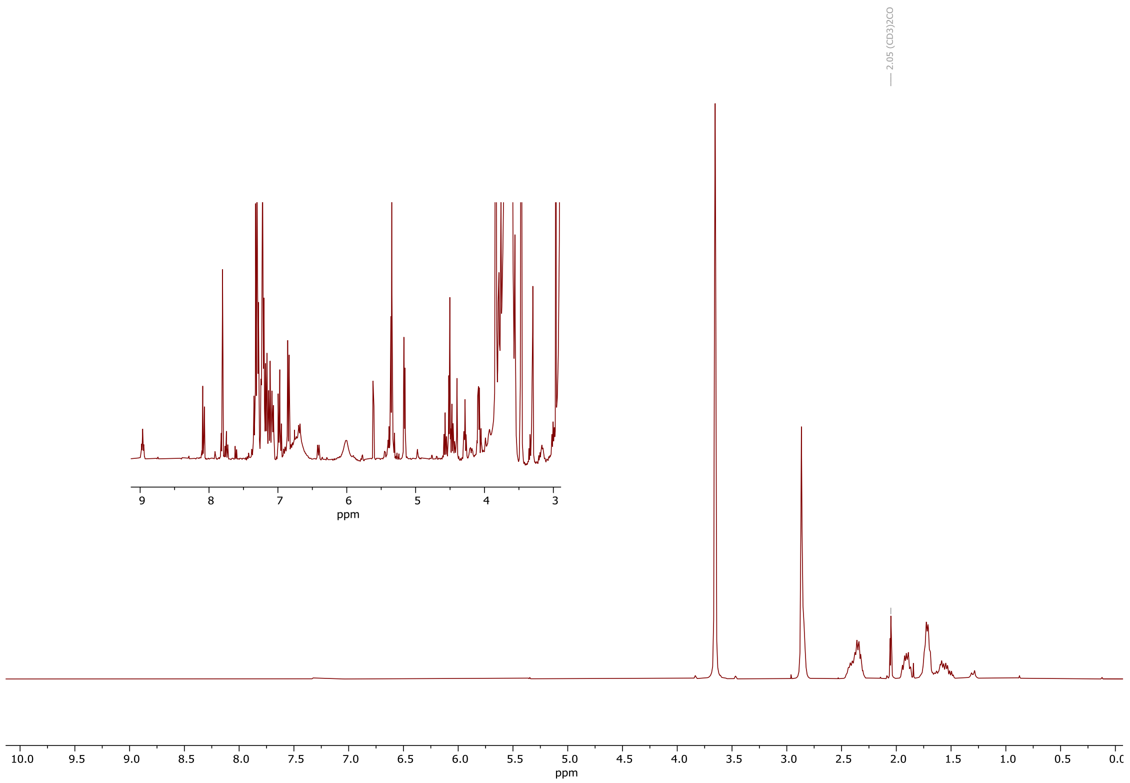

Spectrum S52. ^{1}H NMR (400 MHz, acetone-d_6, 298 K) of $\mathbf{1_H}$ post-sonication before methanol washing

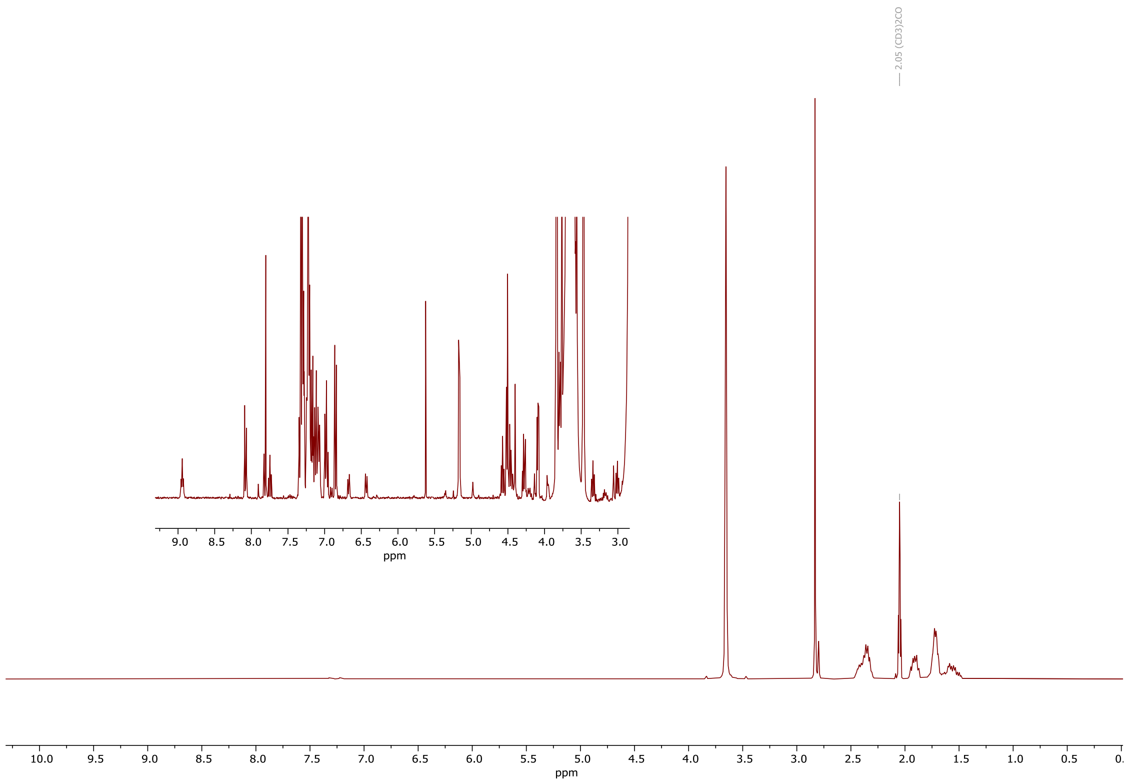

Spectrum S53. ^{1}H NMR (400 MHz, acetone-d_6, 298 K) of $\mathbf{1_H}$ post-sonication after methanol washing

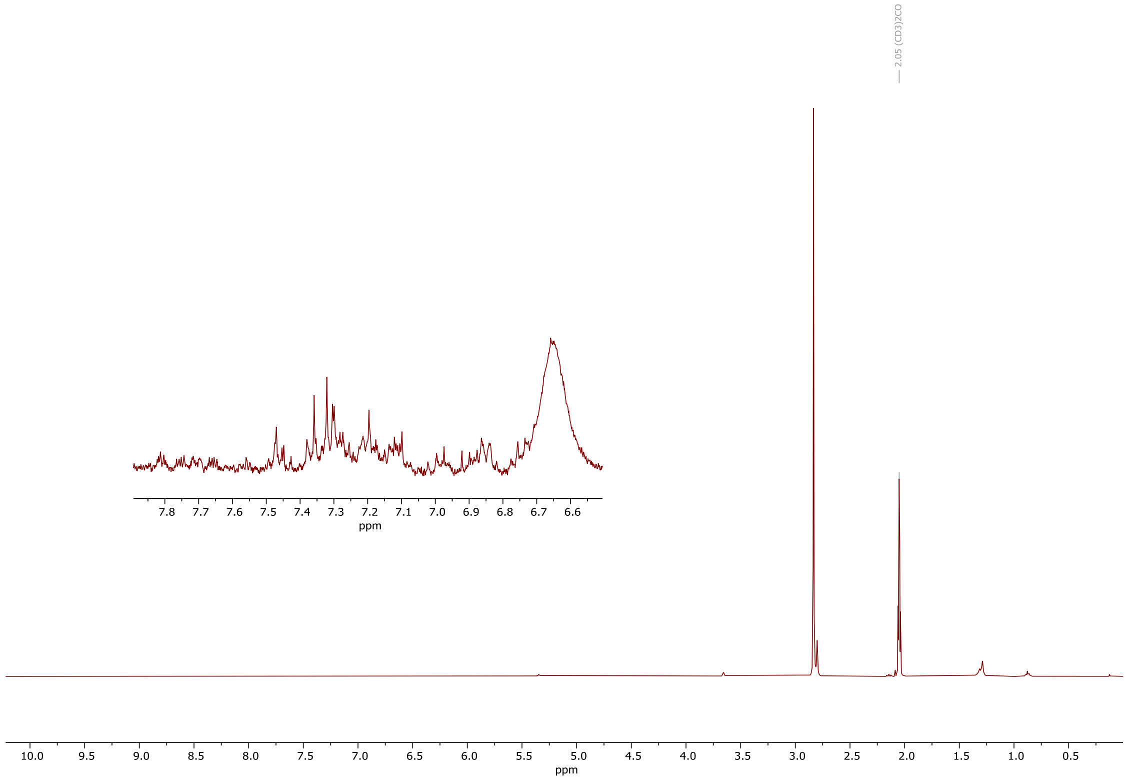

Spectrum S54. ^{1}H NMR (400 MHz, acetone-d_6, 298 K) of concentrated methanol washings

7.3.1 Post-sonication spectra of 1_H (run 3)

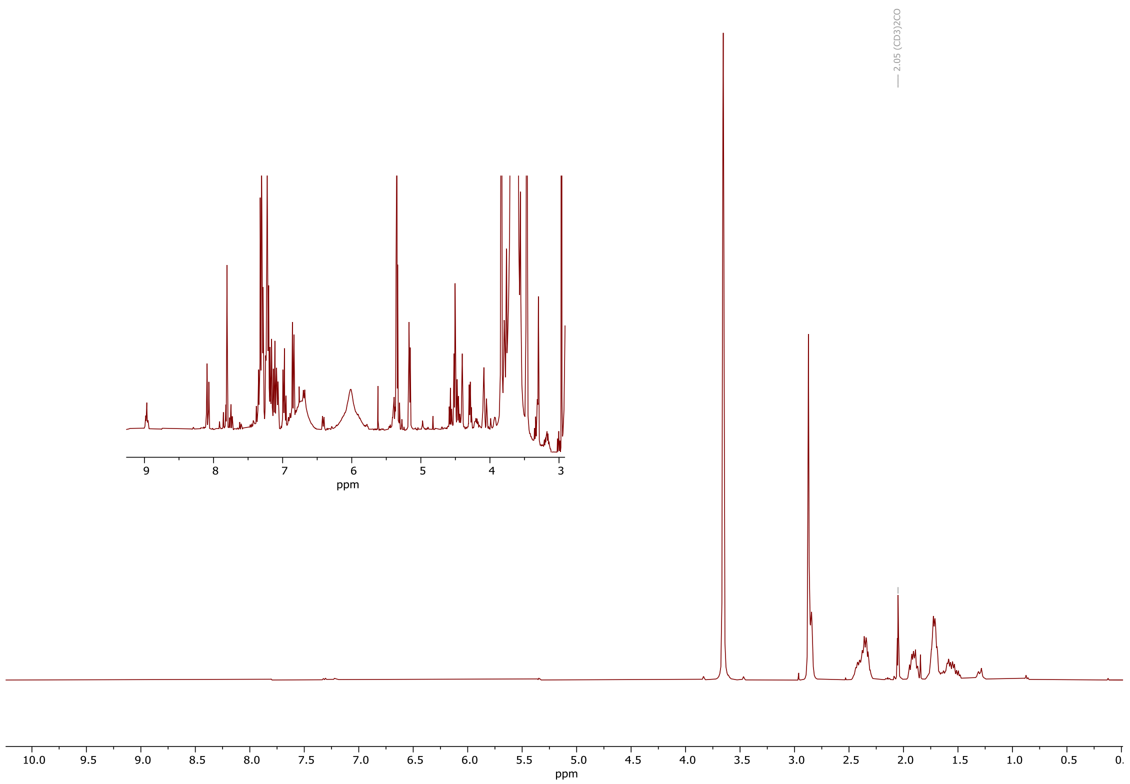

Spectrum S55. ^{1}H NMR (400 MHz, acetone-d$_6$, 298 K) of $\mathbf{1_H}$ post-sonication before methanol washing

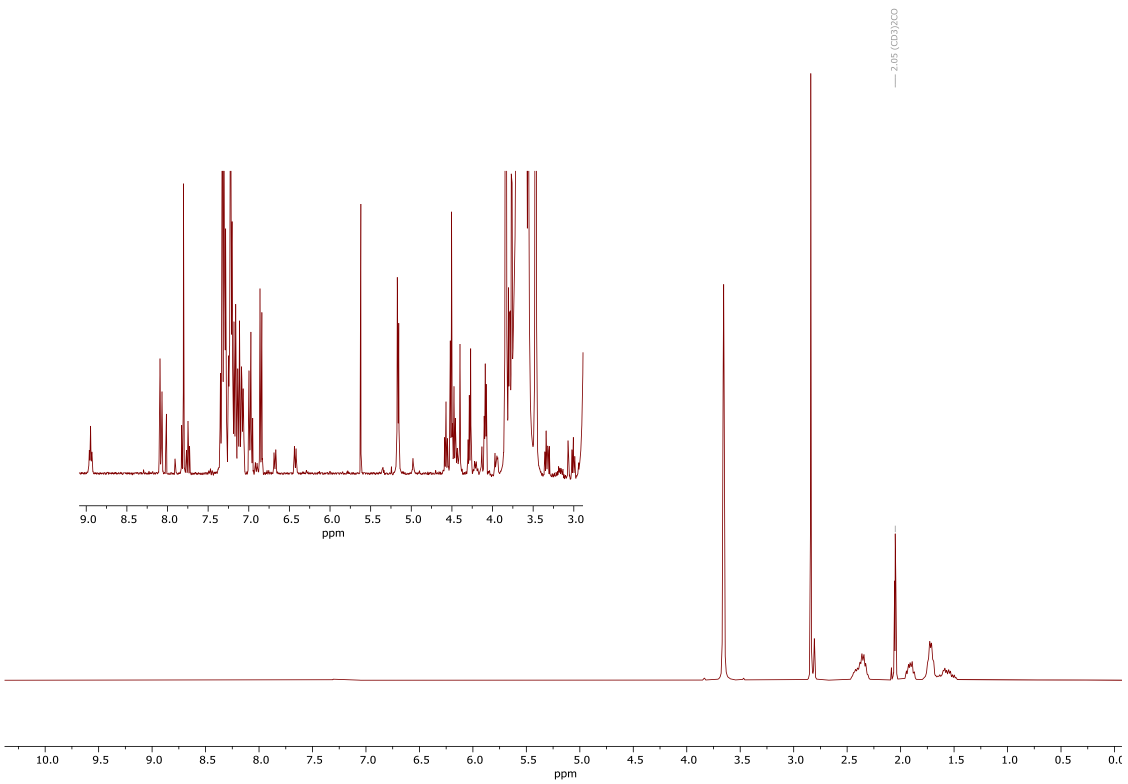

Spectrum S56. ^{1}H NMR (400 MHz, acetone-d$_6$, 298 K) of $\mathbf{1_H}$ post-sonication after methanol washing

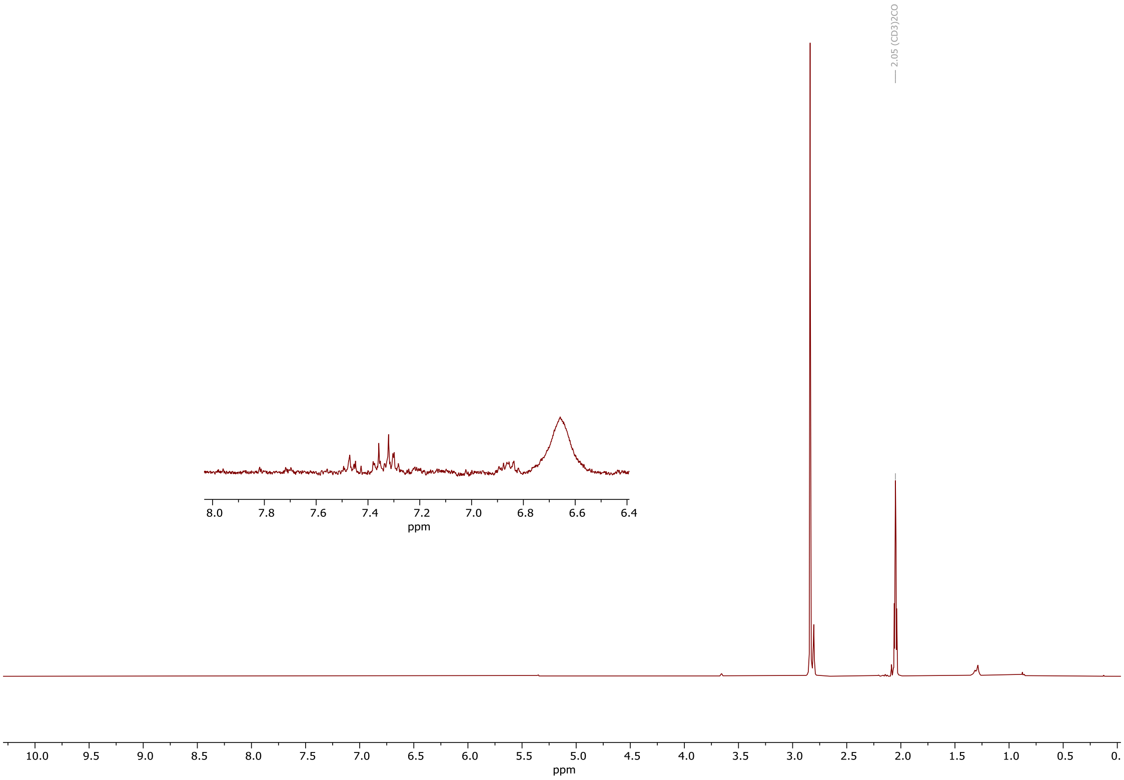

Spectrum S57. ^{1}H NMR (400 MHz, acetone-d_6, 298 K) of concentrated methanol washings

7.3.2 Post-sonication spectra of S23 (run 1)

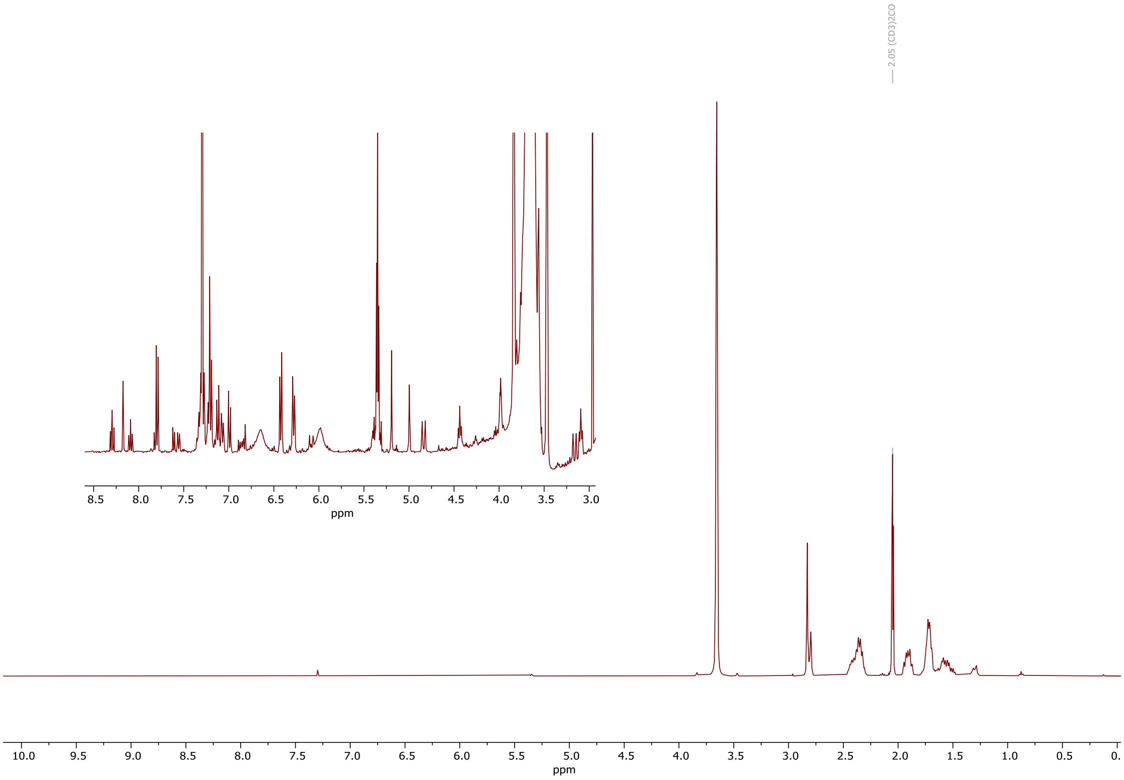

Spectrum S58. ^{1}H NMR (400 MHz, acetone-d_6, 298 K) of **S23** post-sonication before methanol washing

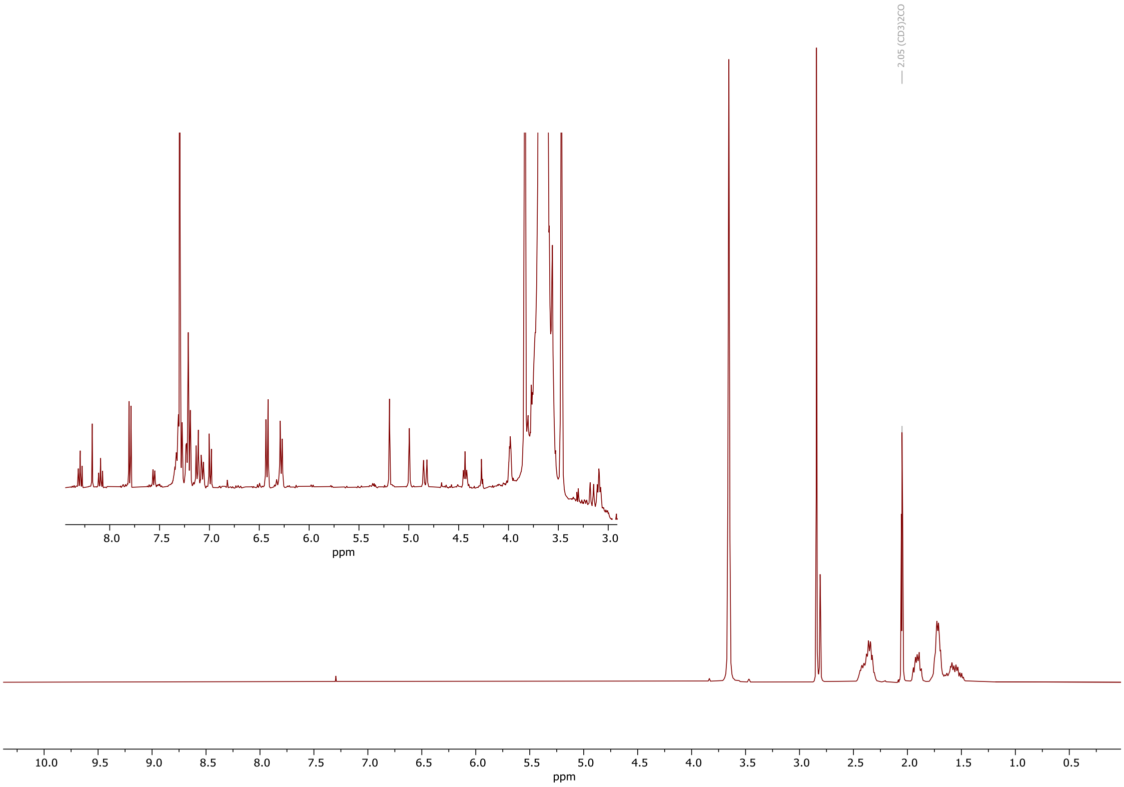

Spectrum S59. ^{1}H NMR (400 MHz, acetone-d$_6$, 298 K) of **S23** post-sonication after methanol washing

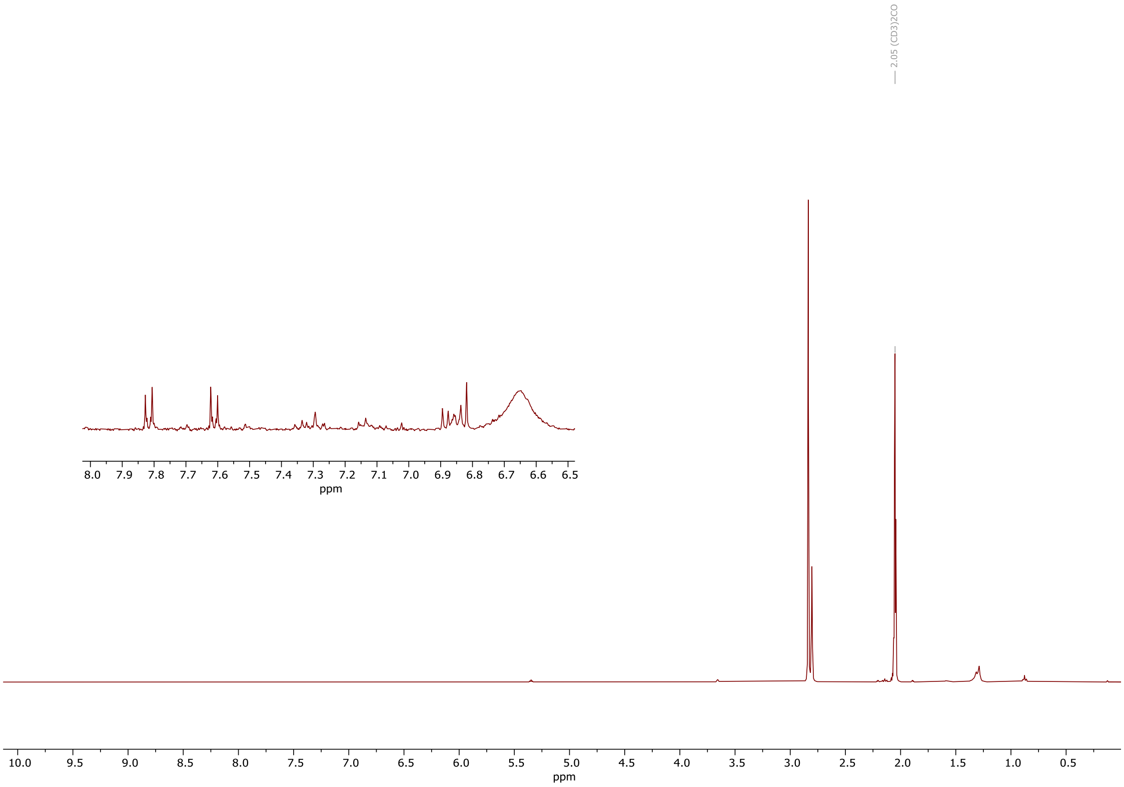

Spectrum S60. ^{1}H NMR (400 MHz, acetone-d$_6$, 298 K) of concentrated methanol washings from **S23** sonication

7.3.1 Post-sonication spectra of S23 (run 2)

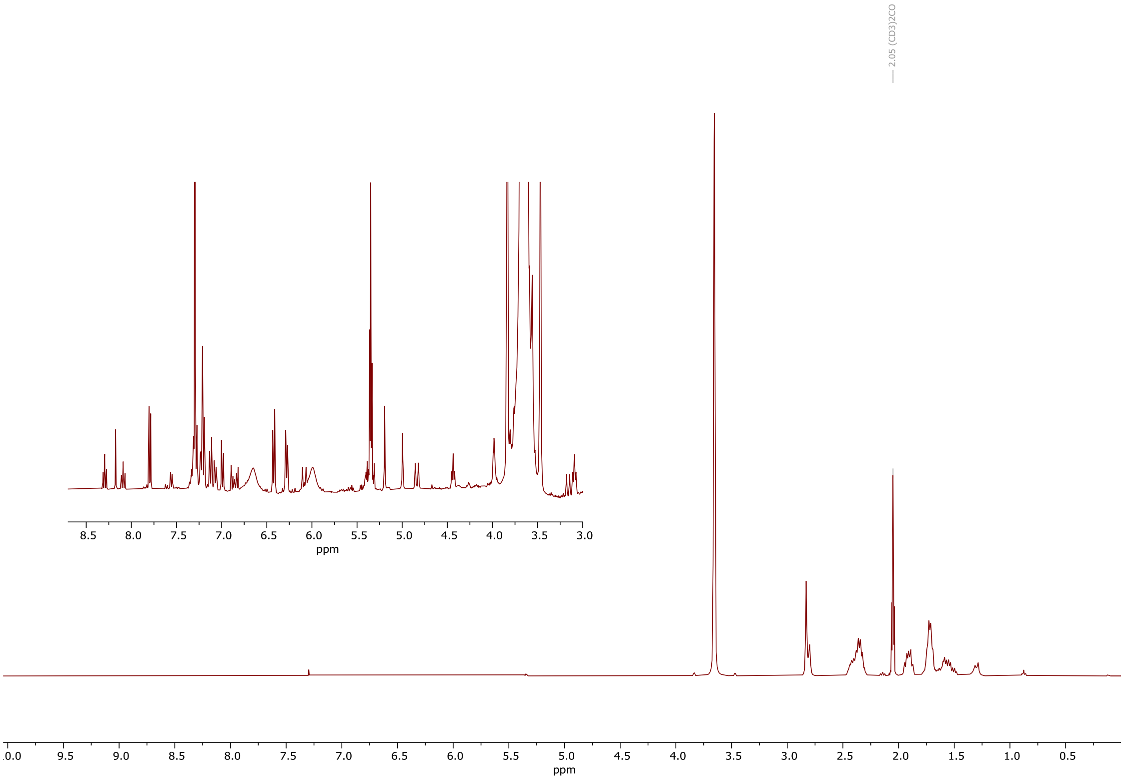

Spectrum S61. ^{1}H NMR (400 MHz, acetone-d$_6$, 298 K) of **S23** post-sonication before methanol washing

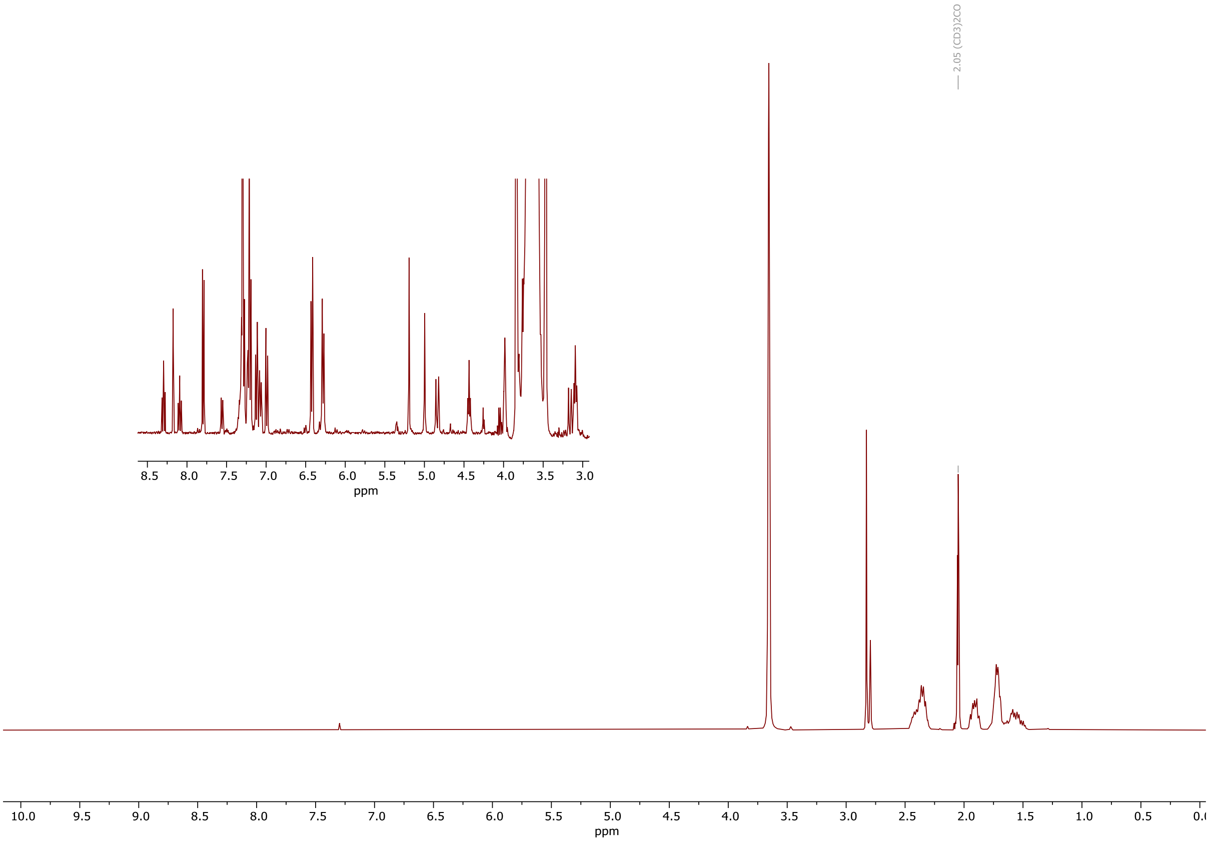

Spectrum S62. ^{1}H NMR (400 MHz, acetone-d$_6$, 298 K) of **S23** post sonication and methanol washing

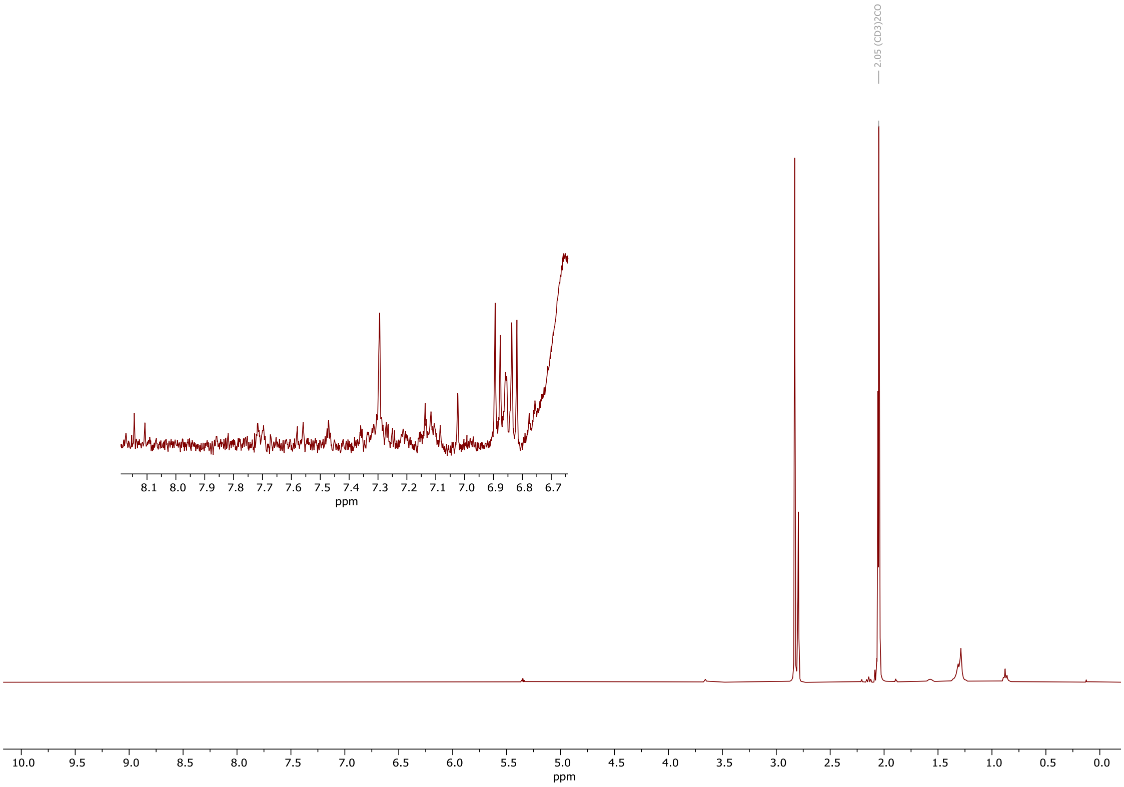

Spectrum S63. ^{1}H NMR (400 MHz, acetone-d_6, 298 K) of **S23** concentrated methanol washings

7.3.1 Post-sonication spectra of S24 (run 3)

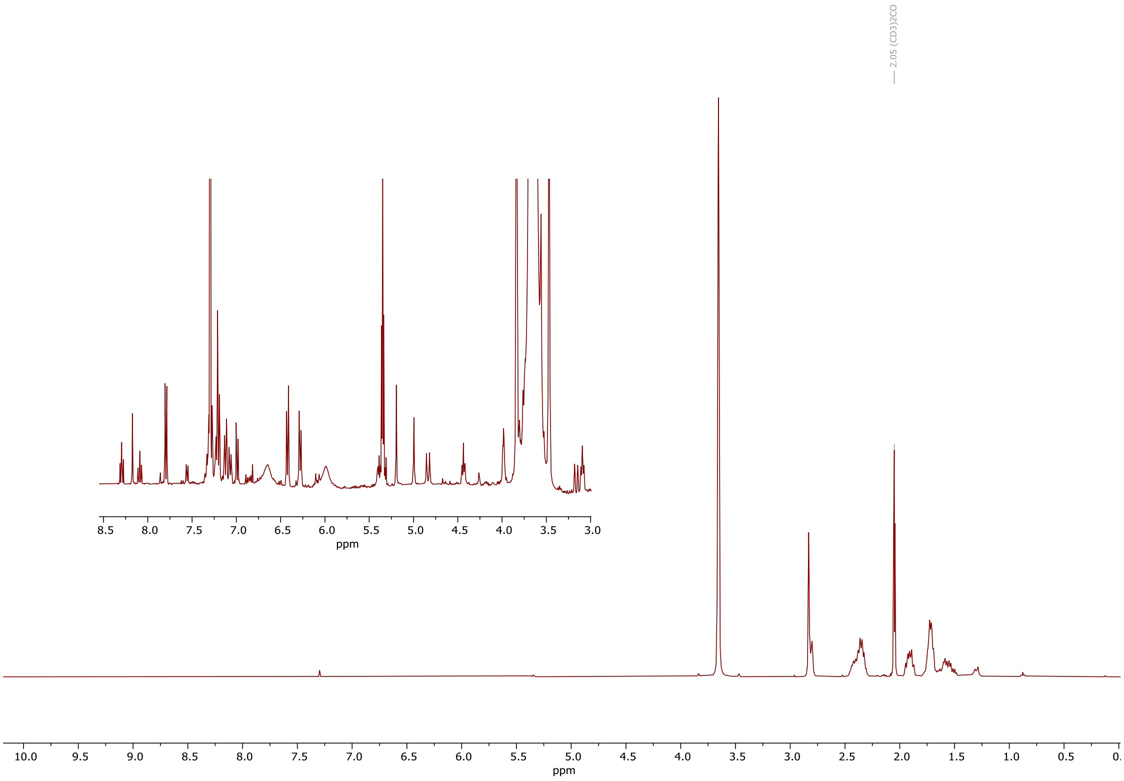

Spectrum S64. ^{1}H NMR (400 MHz, acetone-d_6, 298 K) of **S23** post-sonication before methanol washing

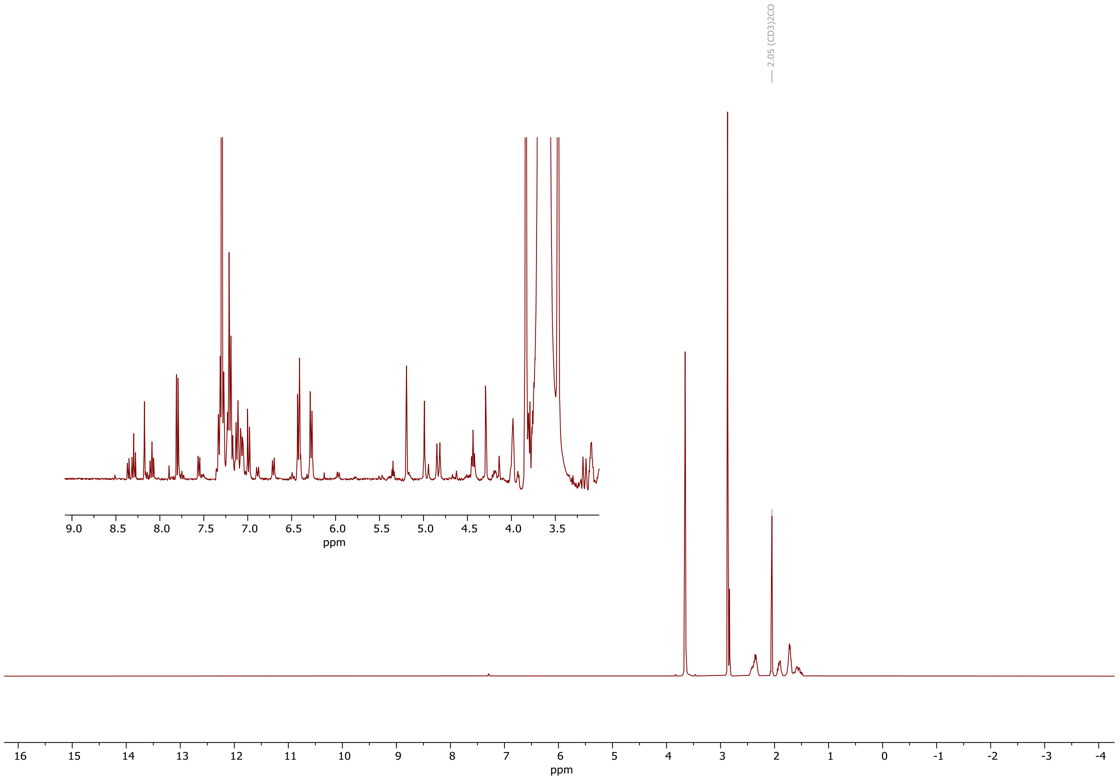

Spectrum S65. ^{1}H NMR (400 MHz, acetone-d_6, 298 K) of **S23** post sonication and methanol washing

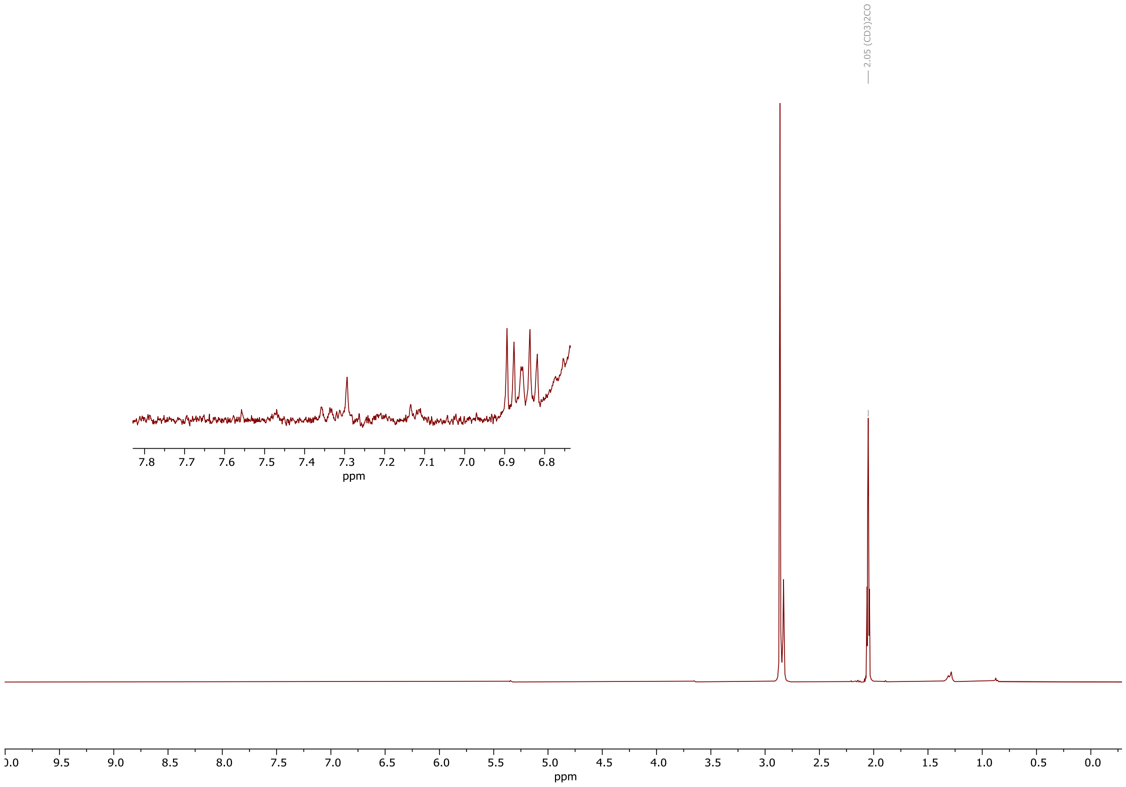

Spectrum S66. ^{1}H NMR (400 MHz, acetone-d_6, 298 K) of **S23** concentrated methanol washings

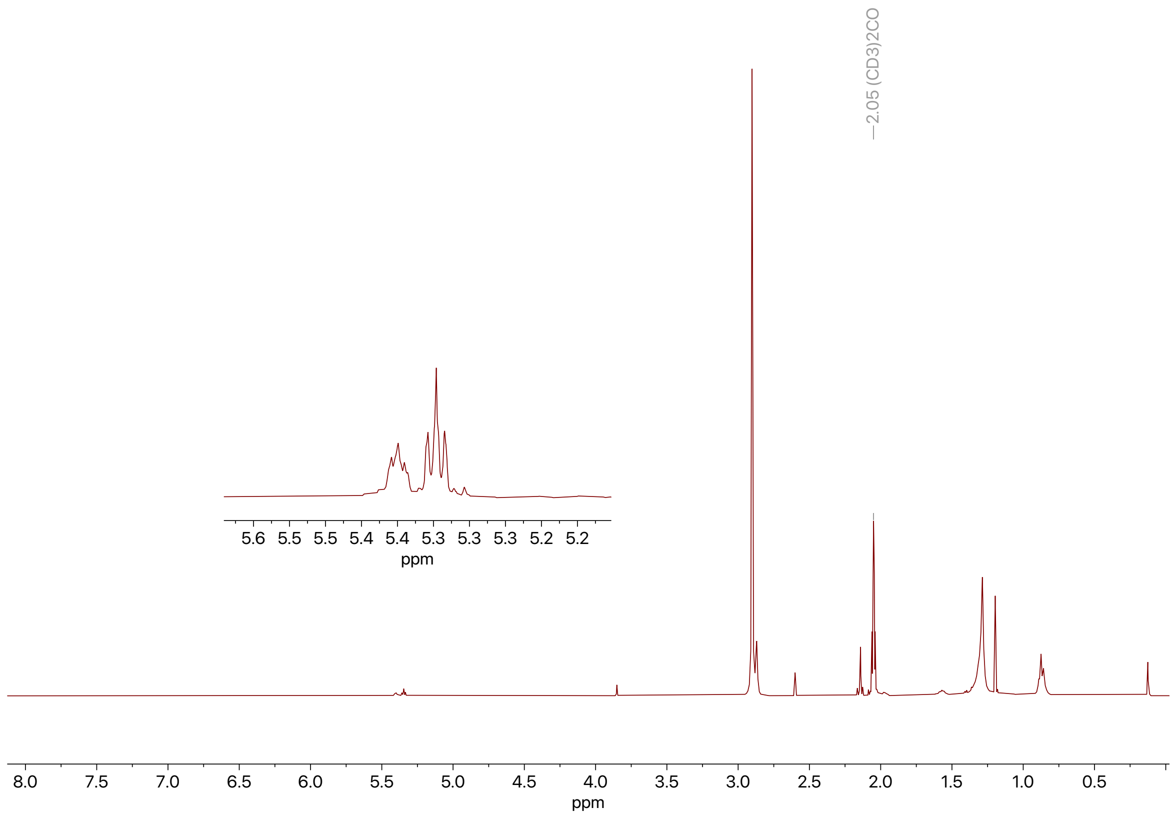

Spectrum S67. ^{1}H NMR (400 MHz, acetone-d_6, 298 K) of concentrated solution after soaking syringe in acetone/DCM for 2 hours.

8 Isotopic patterns of rotaxanes S14, S17, and S20

8.1 Isotopic distribution of S14

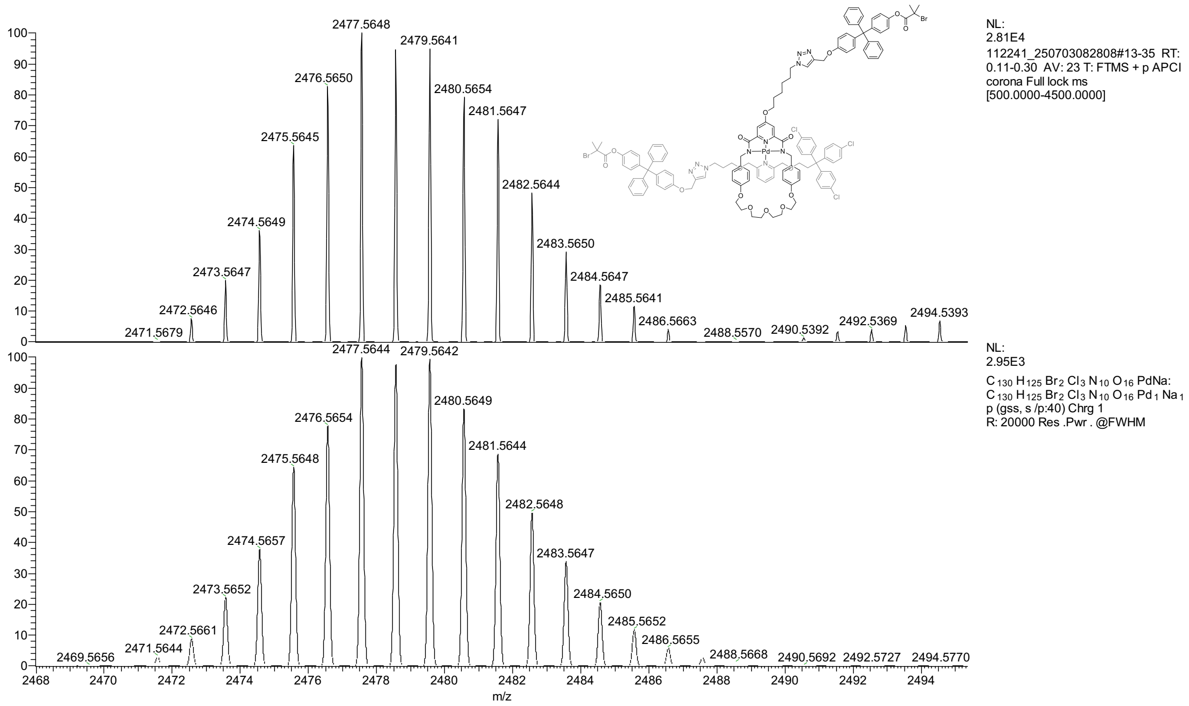

8.2 Isotopic distribution of S17

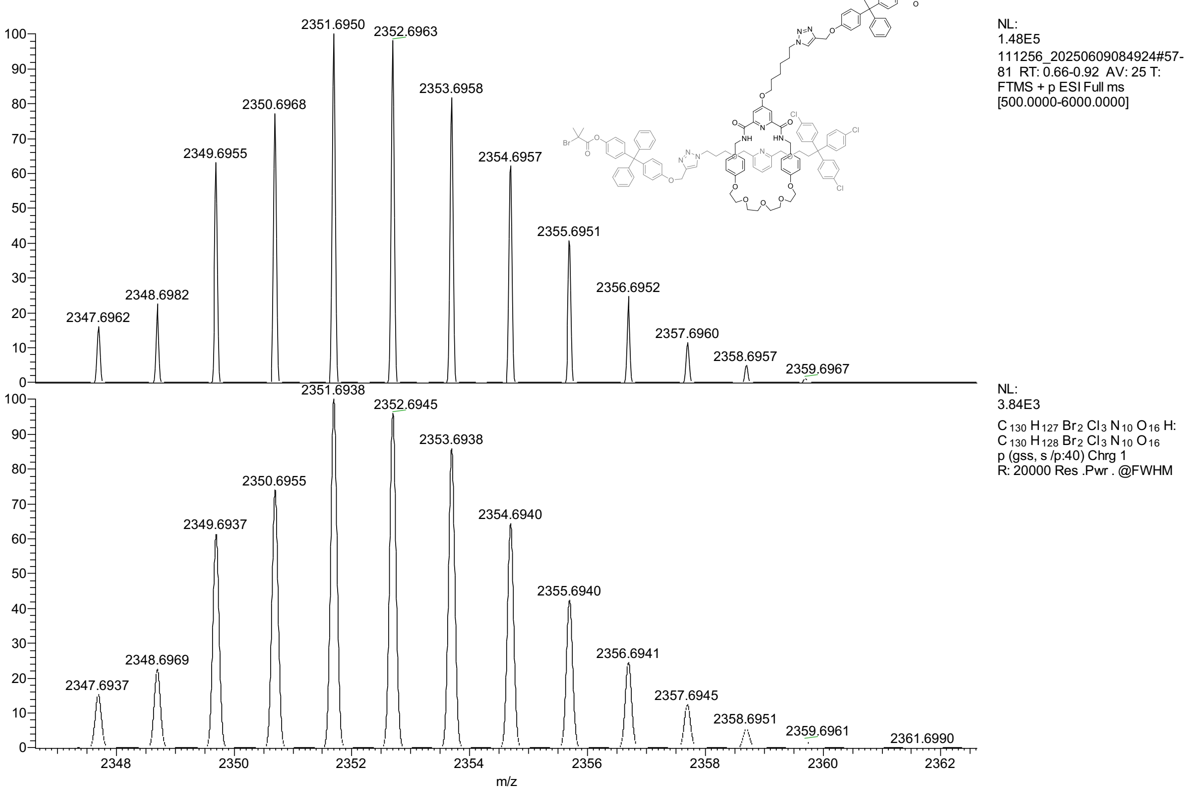

8.3 Isotopic pattern of S20

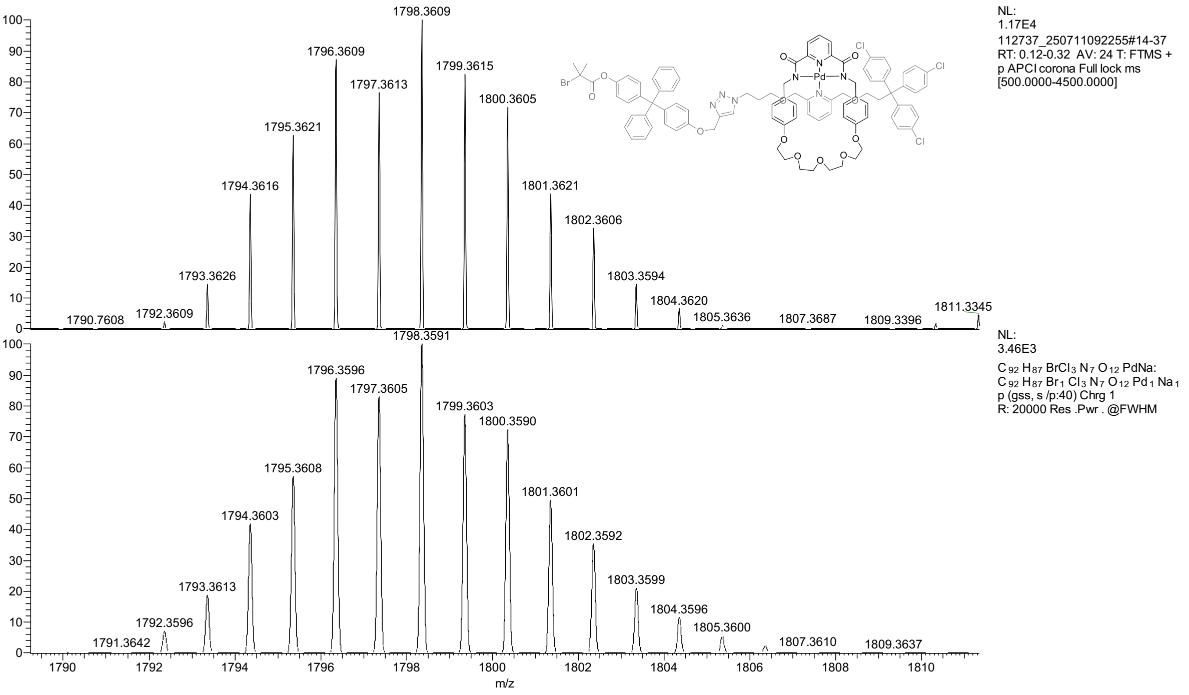

9 References

[1] C. G. Neochoritis, J. Atmaj, A. Twarda-Clapa, E. Surmiak, L. Skalniak, L.-M. Köhler, D. Muszak, K. Kurpiewska, J. Kalinowska-Tłuścik, B. Beck, T. A. Holak, A. Dömling, "Hitting on the move: Targeting intrinsically disordered protein states of the MDM2-p53 interaction" *Eur. J. Med. Chem.* **2019**, *182*, 111588.

[2] F. Coutrot, E. Busseron, "Controlling the Chair Conformation of a Mannopyranose in a Large-Amplitude [2]Rotaxane Molecular Machine" *Chem. – A Eur. J.* **2009**, *15*, 5186–5190.

[3] A. K. Sharma, J. Malineni, S. Box, S. Ghiassinejad, E. van Ruymbeke, C.-A. Fustin, "Synthetic platform for mono-functionalised tridentate macrocycles as key precursors of mechanically-linked macromolecular systems" *Org. Chem. Front.* **2021**, *8*, 2383–2392.

[4] S. Hladysh, D. Václavková, D. Vrbata, D. Bondarev, D. Havlíček, J. Svoboda, J. Zedník, J. Vohlídal, "Synthesis and characterization of metallo-supramolecular polymers from thiophene-based unimers bearing pybox ligands" *RSC Adv.* **2017**, *7*, 10718–10728.

[5] Y. Furusho, T. Matsuyama, T. Takata, T. Moriuchi, T. Hirao, "Synthesis of novel interlocked systems utilizing a palladium complex with 2,6-pyridinedicarboxamide-based tridentate macrocyclic ligand" *Tetrahedron Lett.* **2004**, *45*, 9593–9597.

[6] C. R. Hickenboth, J. S. Moore, S. R. White, N. R. Sottos, J. Baudry, S. R. Wilson, "Biasing reaction pathways with mechanical force" *Nature* **2007**, *446*, 423–427.

[7] M. K. Beyer, "The mechanical strength of a covalent bond calculated by density functional theory" *J. Chem. Phys.* **2000**, *112*, 7307–7312.

[8] J. Ribas-Arino, M. Shiga, D. Marx, "Understanding Covalent Mechanochemistry" *Angew. Chemie Int. Ed.* **2009**, *48*, 4190–4193.
